# Four methylation-driven genes detected by linear discriminant analysis model from early-stage colorectal cancer and their methylation levels in cell-free DNA

**DOI:** 10.3389/fonc.2022.949244

**Published:** 2022-09-05

**Authors:** Lei Zhan, Changjian Sun, Yu Zhang, Yue Zhang, Yuzhe Jia, Xiaoyan Wang, Feifei Li, Donglin Li, Shen Wang, Tao Yu, Jingdong Zhang, Deyang Li

**Affiliations:** ^1^ Medical Oncology Department of Gastrointestinal Cancer, Liaoning Cancer Hospital and Institute, Cancer Hospital of China Medical University, Shenyang, China; ^2^ Clinical Laboratory, Air Force Hospital of Northern Theater, PLA, Shenyang, China; ^3^ Orthopedics Department, Air Force Hospital of Northern Theater, PLA, Shenyang, China; ^4^ Department of Ultrasound and Special Diagnosis, Air Force Hospital of Northern Theater, PLA, Shenyang, China; ^5^ Nursing Department, Air Force Medical Center, PLA, Beijing, China

**Keywords:** DNA Methylation, linear discriminant analysis, carcinogenesis, cell-free DNA - cfDNA, colorectal cancer

## Abstract

The process of colorectal cancer (CRC) formation is considered a typical model of multistage carcinogenesis in which aberrant DNA methylation plays an important role. In this study, 752 methylation-driven genes (MDGs) were identified by the *MethylMix* package based on methylation and gene expression data of CRC in The Cancer Genome Atlas (TCGA). Iterative recursive feature elimination (iRFE) based on linear discriminant analysis (LDA) was used to determine the minimum MDGs (iRFE MDGs), which could distinguish between cancer and cancer-adjacent tissues. Further analysis indicated that the changes in methylation levels of the four iRFE MDGs, ADHFE1-Cluster1, CNRIP1-Cluster1, MAFB, and TNS4, occurred in adenoma tissues, while changes did not occur until stage IV in cell-free DNA. Furthermore, the methylation levels of iRFE MDGs were correlated with the genes involved in the reprogramming process of somatic cells to pluripotent stem cells, which is considered the common signature of cancer cells and embryonic stem cells. The above results indicated that the four iRFE MDGs may play roles in the early stage of colorectal carcinogenesis and highlighted the complicated relationship between tissue DNA and cell-free DNA (cfDNA).

## Introduction

Colorectal cancer (CRC) is the third most prevalent cancer and the third most common cause of cancer-related death worldwide (1). Sufficient research evidence has demonstrated that the occurrence and progression of CRC are a typical multi-stage process that originates with a localized adenoma and then progresses to an intra-mucosal carcinoma, to an invasive lesion, and finally to metastatic cancer (2–4), in which DNA methylation plays important roles (5). DNA methylation can participate in the occurrence and progression of multiple cancers (6, 7), including CRC (8, 9), by modulating genomic functions, especially the expression of oncogenes and tumor suppressor genes (10). For example, hypermethylation of promoter regions of *MLH1* gene results in its silencing and accumulation of DNA mutations; the CpG island methylator phenotype (CIMP), defined as the number of positive methylation markers found at locations of certain genes (11, 12) or even the whole genome (13), can be first observed in early stages of tumorigenesis (9, 12, 14, 15). In addition, epigenetic alterations are also found in early adenoma polyps (16), supporting their essential role in the early stage of oncogenesis. Some gene-specific studies have demonstrated the relationship between increased methylation levels of specific gene promoters and tumor grade or stage (17, 18).

Recent studies have indicated that integrated analyses of DNA methylation and gene expression could better reveal the regulatory function of DNA methylation and effectively predict the prognosis of patients (19–21). The prognostic models of CRC based on methylation-driven genes (MDGs) detected by R package *MethylMix* from DNA methylation and gene expression data have been reported (22–25). However, the above studies mainly focused on the prognosis values of DNA methylation, whereas the roles of gene methylation in colorectal carcinogenesis have not been analyzed based on integrated multi-omics data. The multiple platforms utilized within The Cancer Genome Atlas (TCGA) make it possible to analyze integrated data from multiple sources to identify specific abnormalities most likely to contribute to oncogenic processes.

In the present study, by performing a combined multi-omics analysis based on DNA methylation and gene expression data from the TCGA-COAD dataset, 752 MDGs were called by the *MethylMix* R package (**Table S1**). Iterative recursive feature elimination (iRFE) based on linear discriminant analysis (LDA) was performed to determine the minimal panel of MDGs, which contained four methylation clusters (**Table 1**). Further analysis indicated that the methylation levels of iRFE MDGs changed from the early stage of carcinogenesis. Furthermore, we tried to explore the possible functions of the four MDGs by single-gene Gene Set Enrichment Analysis (GSEA), to provide clues for functional verifications and mechanistic studies in the future.

**Table 1:** Information on iRFE MDGs.

Probe ID	Methylation cluster	Gene symbol
cg01988129	***ADHFE1—Cluster1***	***ADHFE1***
cg08090772	***ADHFE1—Cluster1***	***ADHFE1***
cg07080358	CNRIP1—Cluster1	*CNRIP1*
cg02497758	MAFB	*MAFB*
cg08696192	TNS4	*TNS4*

iRFE, iterative recursive feature elimination; MDGs, methylation-driven genes; bold italics indicated the cluster which contains 2 methylation sites.

The aberrantly methylated genes that play a role in carcinogenesis may be detected in cell-free DNA (cfDNA) because of the release of tumoral DNA in the vascular compartment (26). Hence, MDGs’ methylation levels can potentially be biomarkers for the early diagnosis of CRC. Previous studies have identified some methylated biomarkers for the diagnosis of CRC, such as MYO1-G (27), SEPT9, and SHOX2 (28). Previous studies have built models based on cfDNA methylation to improve the early detection of CRC (29, 30). Based on the MDGs whose methylation levels altered in the early stage of colorectal carcinogenesis, we attempted to construct a model that could catch CRC before it starts.

## Materials and methods

### Data acquisition and preprocessing

The DNA methylation data, gene expression data, and corresponding clinical information of TCGA-COAD samples were automatically downloaded and preprocessed by the R package MethylMix (31, 32). The methylation data of tissue and corresponding sample information of GSE101764 (31), GSE131013 (32), GSE48684 (33), GSE166212 (34), GSE77954 (35) and GSE139404 (36) were downloaded from Gene Expression Omnibus (GEO) (https://www.ncbi.nlm.nih.gov/geo/browse/?view=series). The methylation levels of MDGs (see below) were extracted from the seven datasets of tissue as “MDGs data” for further analysis. CfDNA dataset GSE149438 (37) was downloaded from GEO, and the methylation levels of iRFE MDGs (see below) were extracted from raw data. The probes containing NA value(s) were removed. Principal component analysis (PCA) was performed with the *prcomp* function to detect outliers from all eight methylation datasets. The methylation level of a cluster was defined as the average methylation level of the sites in this cluster. The usages and other information of the eight datasets are summarized in **Table S2**. The ethics committee approval for this study was not necessary because the data were obtained from TCGA and GEO.

### Identification of DNA methylation-driven genes and enrichment analyses

The *MethylMix* package based on R 4.1.1 was used to comprehensively analyze COAD’s integrated DNA methylation and gene expression data from TCGA (38, 39). The methylation clusters that were considered as the clusters in MDGs by the *MethylMix* package were extracted from the analysis results. Mixture models of MDGs were plotted with the *MethylMix_PlotModel* function of the *MethylMix* package. The averaged methylation level of all MDGs in the same gene was regarded as the methylation level of this gene (MDGs). GO and Kyoto Encyclopedia of Genes and Genomes (KEGG) enrichment analyses of MDGs were performed with R package *clusterProfiler* (40). GSEA of the methylation difference of all genes was performed with *clusterProfiler*, too.

### Establishment, testing, and validation of linear discriminant analysis model based on methylation-driven genes

The randomly selected 70% samples from TCGA-COAD methylation data of MDGs were used to establish the LDA model by the *lda* function of the *MASS* R package. The classification performance of the LDA model was tested with the other 30% samples of TCGA-COAD methylation data and was validated with independent datasets GSE101764, GSE131013, and GSE48684 by receiver operating characteristic (ROC) curves. In addition, randomly selected methylation clusters from the TCGA-COAD dataset were used to establish LDA models, which were validated with the above independent datasets by ROC curves. The random selection and LDA model construction were repeated 300 times in the train set and validated 100 times by ROC curves in datasets GSE101764, GSE131013, and GSE48684.

### Recursive feature elimination and iterative recursive feature elimination

RFE was performed by *rfe* function from the *caret* R package to obtain the most contributing methylation clusters for distinguishing between cancer and cancer-adjacent tissues based on the TCGA-COAD dataset of MDGs. The *rfe* function was executed 100 times and obtained 100 sets of the most contributing methylation clusters. Only the clusters that were contained in the above 50 sets were regarded as “RFE MDGs”.

The methylation data of the above RFE MDGs were inputted into the *rfe* function again to obtain the second-generation RFE MDGs, and the cycle repeats until the number of RFE MDGs did not decrease anymore. The final RFE MDGs produced by this progress, which was defined as an “iterative RFE” process, were regarded as “iRFE MDGs”. The iRFE MDGs were used to establish a final LDA model (iRFE LDA model), which was tested with part of TCGA-COAD samples and validated with independent datasets. The iRFE LDA model was also used to distinguish between normal and adenoma tissues of GSE48684, GSE77954, and GSE166212, between low- and high-grade adenoma tissues of GSE139404, as well as the cfDNA from healthy normal and CRC patients with different stages.

### Correlation analysis between methylation levels of iterative recursive feature elimination methylation-driven genes

Correlations between the methylation levels of iRFE MDGs were plotted with the *ggpairs* function of the *GGally* R package. In addition, correlation coefficients were calculated between the methylation levels of randomly selected MDGs. This random selection and correlation coefficient calculation process was repeated 100 times in the data of TCGA-COAD, GSE101764, GSE131013, and GSE48684. The heatmap that visualizes the correlation coefficients between the methylation levels of randomly selected MDGs was plotted with the *pheatmap* function from the *pheatmap* R package.

### Single-gene gene set enrichment analysis

The correlation coefficients between the methylation levels of the four iRFE MDGs and all the other methylation clusters were calculated respectively. Single-gene GSEA of these four genes was performed with *GSEA* function of R package *clusterProfiler* based on the sorted correlation coefficients to determine whether prior defined gene sets showed statistically significant enrichment. The gene sets with the top 10 normalized enrichment score (NES) values were plotted with the *gseaplot2* function of the R package *clusterProfiler*.

### Statistical analysis

The *p*-values in enrichment analysis and GSEA were calculated with the R package *ClusterProfiler*. One-tailed one-sample t-test was performed manually with R. The *p*-values of comparisons in boxplots were calculated with R package *ggpubr*. Correlation analysis was performed with R package *ggpairs*. A value of *p* < 0.01 was considered statistically significant.

## Results

### Methylation-driven gene identification and enrichment analysis

**Figure 1:**
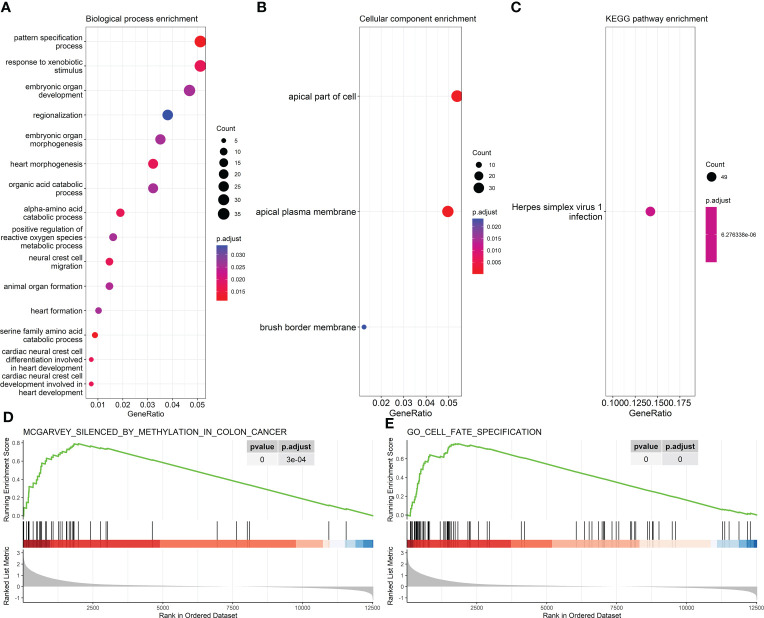
Enrichment results of 761 methylation-driven genes (MDGs). **(A, B)** GO enrichment analysis. **(A)** Biological process. **(B)** Cellular component. **(C)** KEGG pathway enrichment analysis. **(D, E)** Representative results of GSEA. GO, Gene Ontology; KEGG, Kyoto Encyclopedia of Genes and Genomes; GSEA, Gene Set Enrichment Analysis.

A total of 752 MDGs, involving 1,107 methylation sites that could be combined with 761 methylation clusters, were identified by the *MethylMix* R package based on TCGA-COAD methylation and gene expression datasets (**Table S1**; bold italics indicated the cluster which contains above 1 methylation site). The outliers were not detected in the methylation data of MDGs from samples of all eight datasets by PCA (**Figure S1**). GO and KEGG enrichment analyses indicated that the MDGs significantly enriched in the process of cell morphogenesis and cell polarity (**Figures 1A–C**). GSEA identified the gene set silenced by methylation in colon cancer cell line HCT116 (**Figure 1D**), which was reported in a previous study (41). In addition, MDGs were enriched in the gene set related to cell fate specification (**Figure 1E**).

### Construction, testing, and validation of linear discriminant analysis model based on 752 methylation-driven genes

**Figure 2:**
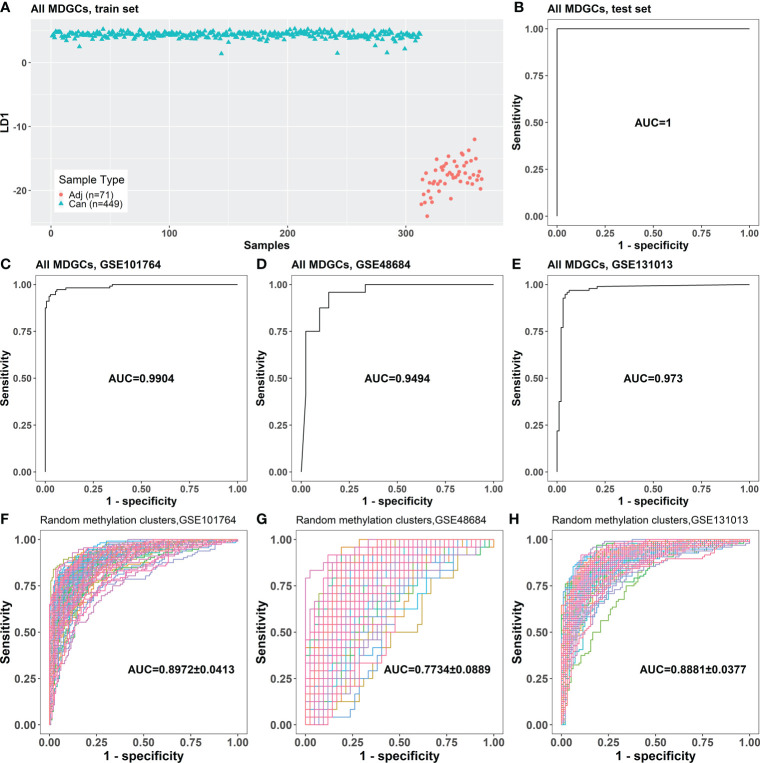
The classification performance of the LDA model based on 752 MDGs. **(A)** The LDA model established with the samples in the train set. **(B)** The classification performance of the LDA model on the test set. **(C–E)** The classification performances of the LDA model for cancer and cancer-adjacent samples from GSE101764, GSE48684, and GSE131013. **(F–H)** The classification performances of the LDA models based on randomly selected 761 genes on GSE101764, GSE48684, and GSE131013 datasets (100 repeats). Adj, cancer-adjacent tissues; Can, cancer tissues; LDA, linear discriminant analysis; MDGs, methylation-driven genes.

The samples in the train set could be correctly divided into cancer and cancer-adjacent tissues in the LDA model based on 752 MDGs (**Figure 2A**). This LDA model could distinguish between cancer and cancer-adjacent tissues in the test set (area under the curve (AUC) = 1; **Figure 2B**). Moreover, the LDA model based on the 752 MDGs could also distinguish between cancer and cancer-adjacent tissues in independent validation sets GSE101764, GSE48684, GSE131013 (AUC = 0.9904, 0.9494, and 0.973, respectively; **Figures 2C–E**), in which the AUCs were significantly larger than those of the LDA models based on randomly selected methylation clusters (*p* = 0.013, 0.025, and 0.013, respectively; one-tailed one-sample t-test; **Figures 2F–H**).

### The screening of methylation-driven genes by iterative recursive feature elimination

**Figure 3:**
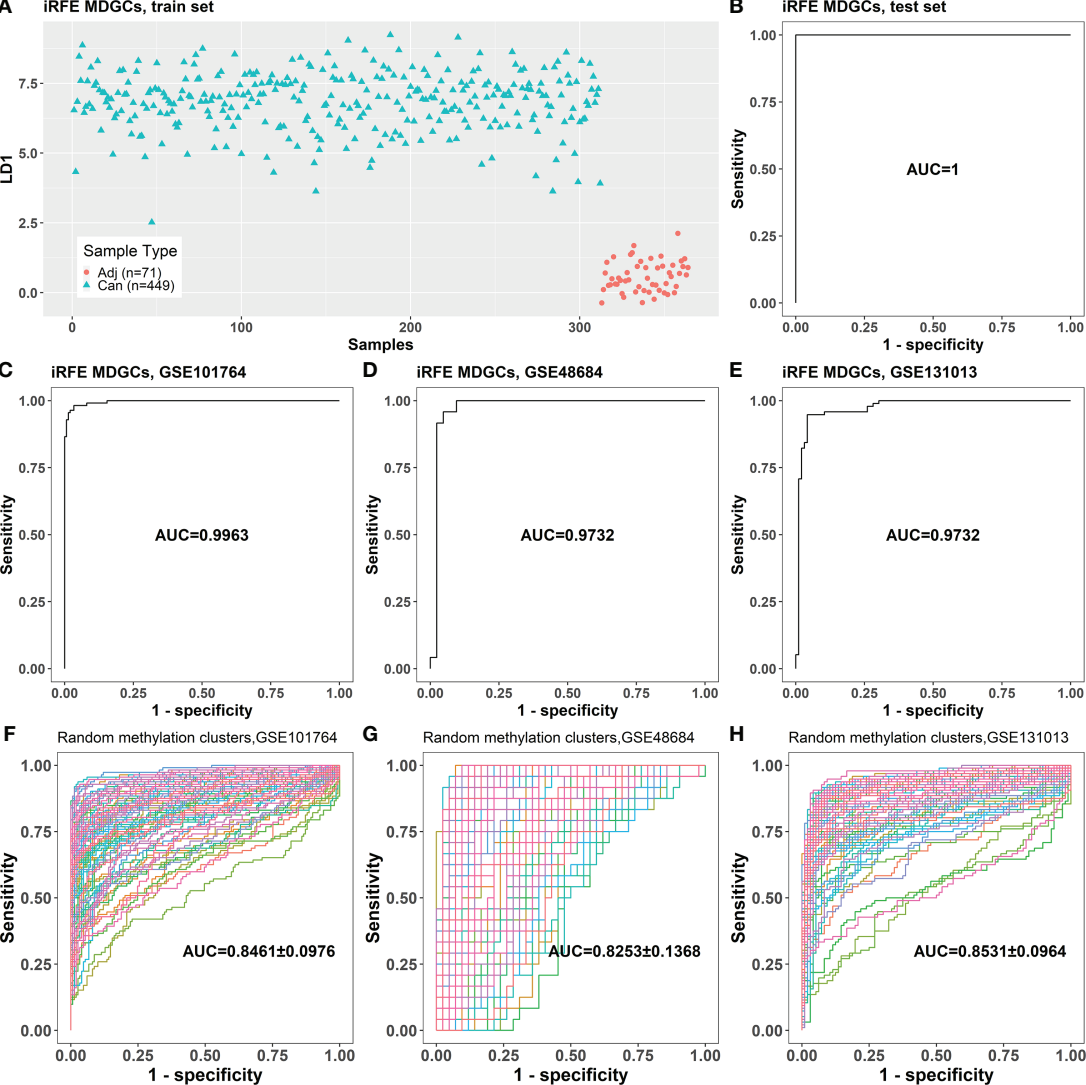
The classification performance of the LDA model based on the four iRFE MDGs. **(A)** The LDA model established with the samples in the train set. **(B)** The classification performance of the LDA model on the test set. **(C–E)** The classification performances of the LDA model for cancer and cancer-adjacent samples from GSE101764, GSE48684, and GSE131013. **(F–H)** The classification performances of the LDA models based on randomly selected four genes from MDGs on GSE101764, GSE48684, and GSE131013 datasets (100 repeats). Adj, cancer-adjacent tissues; Can, cancer tissues; LDA, linear discriminant analysis; iRFE, iterative recursive feature elimination; MDGs, methylation-driven genes.

RFE was performed iteratively to screen the minimum panel of MDGs that could distinguish between cancer and cancer-adjacent tissues. The methylation cluster contained in the final panel, ADHFE1-Cluster1, CNRIP1-Cluster1, MAFB, and TNS4, was defined as “iRFE MDGs” (**Table 1**). The β mixture models and correlation plots of iRFE MDGs are displayed in **Figure S2**. The train set could be divided into cancer and cancer-adjacent tissues correctly in the LDA model based on iRFE MDGs (**Figure 3A**). This iRFE LDA model could distinguish between cancer and cancer-adjacent tissues in the test set (AUC = 1; **Figure 3B**) and independent validation sets GSE101764, GSE48684, and GSE131013 (AUC = 0.9963, 0.9732, and 0.9732, respectively; **Figures 3C–E**), in which the AUCs tended to be larger than those of the LDA models based on randomly selected MDGs (*p* = 0.064, 0.14, and 0.11, respectively; one-tailed one-sample t-test; **Figures 3F–H**). Since the methylation levels of all MDGs were significantly different between cancer and cancer-adjacent tissues, it is expected that the AUCs of the iRFE models were not significant, although they tended to be larger than those of LDA models based on randomly selected MDGs.

### The alteration of methylation levels of iterative recursive feature elimination methylation-driven genes occurred in the early stage of colorectal carcinogenesis

**Figure 4:**
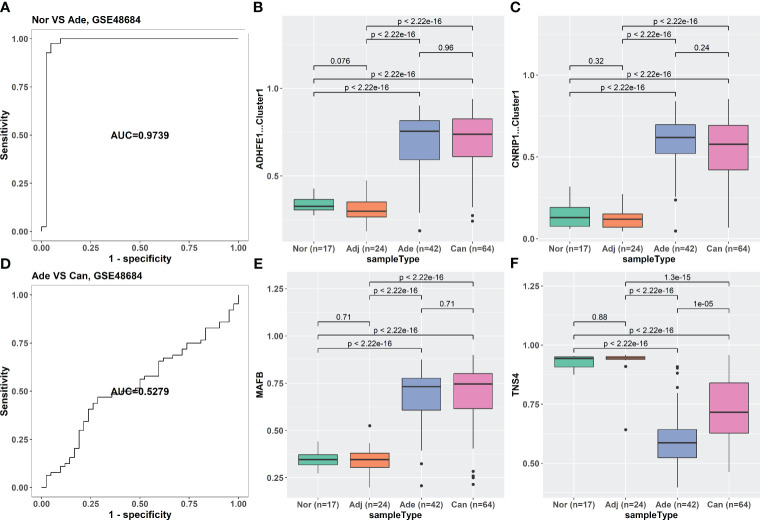
The classification ability and methylation levels of the four iRFE MDGs in GSE48684. **(A)** The classification performance of the LDA model for normal and adenoma samples. **(D)** The classification performance of the LDA model for adenoma and cancer tissues. **(B, C, E, F)** The methylation levels of the four iRFE MDGs in different tissues. **(B)** ADHFE1-Cluster1. **(C)** CNRIP1-Cluster1. **(E)** MAFB. **(F)** TNS4. Nor, healthy normal tissues; Adj, cancer-adjacent tissues; Ade, adenoma tissues; Can, cancer tissues; iRFE, iterative recursive feature elimination; MDGs, methylation-driven genes; LDA, linear discriminant analysis.

**Figure 5:**
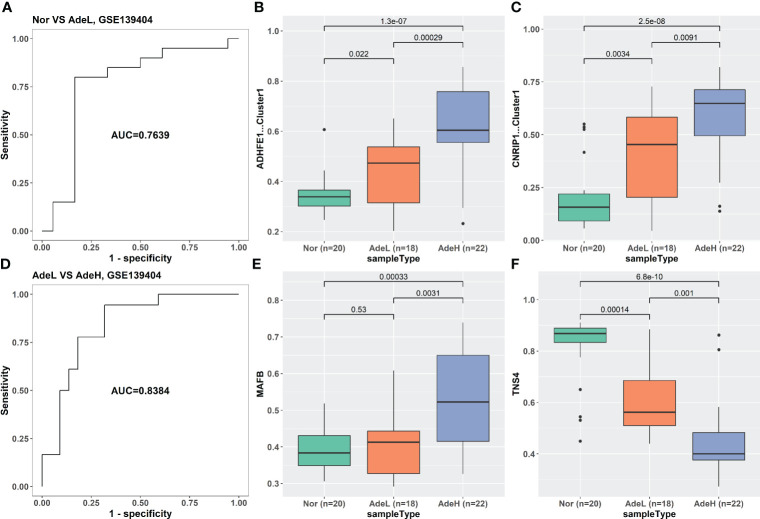
The classification ability and methylation levels of the four iRFE MDGs in dataset GSE139404. **(A)** The classification performance of the LDA model for normal and low-grade adenoma samples. **(D)** The classification performance of the LDA model for low- and high-grade adenoma tissues. **(B, C, E, F)** The methylation levels of the four iRFE MDGs in different tissues. **(B)** ADHFE1-Cluster1. **(C)** CNRIP1-Cluster1. **(E)** MAFB. **(F)** TNS4. Nor, healthy normal tissues; AdeL, low-grade adenoma tissues; AdeH, high-grade adenoma tissues; iRFE, iterative recursive feature elimination; MDGs, methylation-driven genes; LDA, linear discriminant analysis.

The iRFE LDA model could distinguish not only between cancer and cancer-adjacent tissues but also between cancer and healthy normal tissues (**Figures S3A, C**). However, healthy normal and cancer-adjacent tissues could not be distinguished by the iRFE LDA model (**Figures S3B, D**). Correspondingly, the methylation levels of the four iRFE MDGs were not significantly different between healthy normal and cancer-adjacent samples (**Figures S3E–H**). Based on these results, we regarded healthy normal and cancer-adjacent tissues as homogeneous normal tissues in subsequent analysis. The iRFE LDA model was used to distinguish between normal and adenoma samples and between adenoma and cancer samples to explore the stage in which the methylation levels of iRFE MDGs changed. The distinguished results indicated that the change began from adenoma, namely, the early stage of colorectal carcinogenesis (**Figures 4A, D**, **S4A, D**, **S5A, D**). Correspondingly, the differences in methylation levels of iRFE MDGs were greater and more significant between normal tissue and adenoma than between adenoma and cancer (**Figures 4B, C, E, F**, **S4B, C, E, F**, **S5B, C, E** and **F**). Further analysis based on low- and high-grade adenoma showed that the iRFE LDA model could distinguish between normal and low-grade adenoma, and between low- and high-grade adenoma, although the performances were poorer than between normal and cancer tissues (AUC = 0.7639, and 0.8384, respectively; **Figures 5A, D**). Correspondingly, MDGs’ methylation levels changed in low-grade and high-grade adenoma compared with normal tissues (**Figures 5B, C, E, F**). All these results indicated that the methylation levels of iRFE MDGs changed during the complete process from normal tissue to adenoma, in other words, the early stage of colorectal carcinogenesis.

The results of the classification of clinical phenotypes with both linear (LDA, **Figure S6**) and non-linear (k-nearest neighbor (KNN); **Figure S7**) models based on four iRFE MDGs indicated that although the four MDGs could distinguish normal and adenoma, they were not associated with tumor phenotypes, which was consistent with the observation that the four iRFE MDGs played roles only in the early stage of carcinogenesis. The results of the prognostic analysis (**Tables S3–S6**) further reinforced this observation.

### The methylation levels of iterative recursive feature elimination methylation-driven genes were significantly correlated with each other

**Figure 6:**
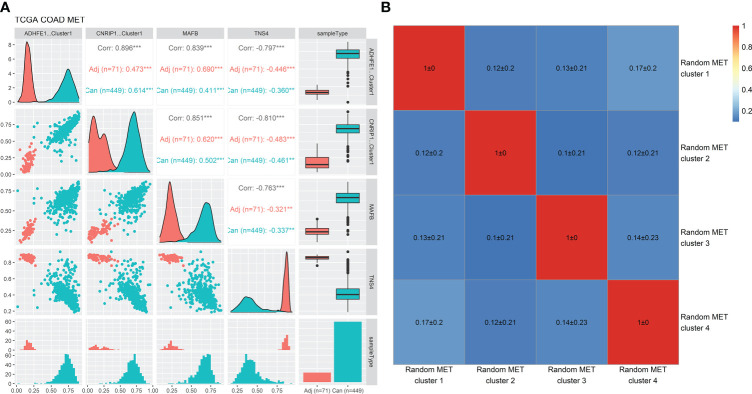
Correlation analysis of the methylation levels of the four iRFE MDGs and randomly selected MDGs in the TCGA-COAD dataset. **(A)** Correlation plots and distribution of the methylation levels of the four iRFE MDGs. **(B)** Averaged correlation coefficients of methylation levels of randomly selected MDGs (100 repeats). iRFE, iterative recursive feature elimination; MDGs, methylation-driven genes. **P < 0.01; ***P < 0.001.

The methylation levels of the four iRFE MDGs were significantly correlated with each other in both cancer and cancer-adjacent samples (**Figure 6A**). Correspondingly, the expression levels of the four iRFE MDGs were also significantly correlated (**Figure S8**). To confirm the specification of this correlation, the correlation coefficients of the methylation levels of four randomly selected MDGs were calculated. Averaged correlation coefficients of 100 repeats were displayed with a heatmap, as shown in **Figure 6B**. Similar results were obtained from independent datasets GSE101764, GSE131013, and GSE48684, as shown in **Figures S9–S11**.

### Single-gene Gene Set Enrichment Analysis of the four iterative recursive feature elimination methylation-driven genes

**Figure 7:**
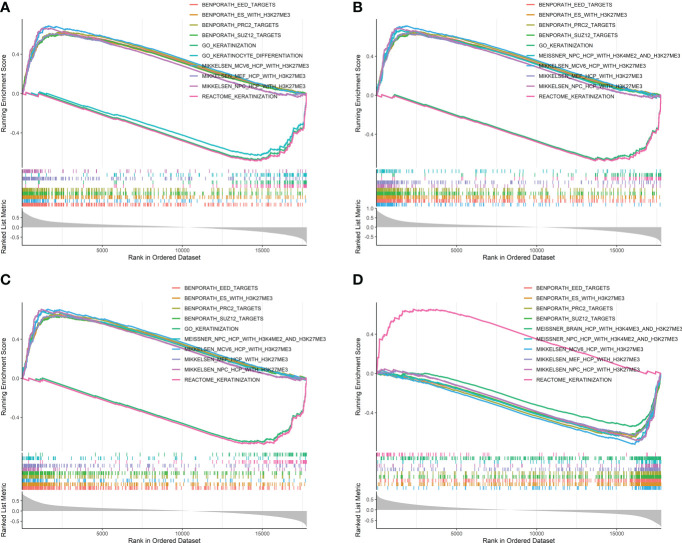
Single-gene GSEA results based on the correlation coefficients between the methylation levels of the four iRFE MDGs and those of other genes in the TCGA-COAD dataset. The gene sets with the top 10 NES values are shown. **(A)** *ADHFE1*. **(B)** *CNRIP1*. **(C)** *MAFB*. **(D)** *TNS4*. NES, normalized enrichment score; GSEA, Gene Set Enrichment Analysis; iRFE, iterative recursive feature elimination; MDGs, methylation-driven genes.

Single-gene GSEA based on methylation levels revealed that the genes whose methylation levels were significantly positively correlated with ADHFE1-Cluster1, CNRIP1-Cluster1, and MAFB were all significantly enriched in gene sets MIKKELSEN_MCV6_HCP_WITH_H3K27ME3, BENPORATH_EED_TARGETS, BENPORATH_PRC2_TARGETS, BENPORATH_ES_WITH_H3K27ME3, BENPORATH_SUZ12_TARGETS, MIKKELSEN_MEF_HCP_WITH_H3K27ME3, and MIKKELSEN_NPC_HCP_WITH_H3K27ME3, which are involved in the reprogramming process of a somatic cell to pluripotent stem cell (42) and are considered as the common signatures of cancer cell and embryonic stem (ES) cell (43). The genes significantly enriched in the REACTOME_KERATINIZATION gene set tended to be positively correlated with the methylation level of TNS4 (**Figure 7**). Similar results were obtained from independent datasets GSE101764, GSE131013, and GSE48684, as shown in **Figures S12–S14**. Full GSEA results (data not shown) indicated that significantly enriched gene sets focused on cell differentiation and development, cell fate specification, and antimicrobial humoral immune response.

### The methylation levels of iterative recursive feature elimination methylation-driven genes changed in cell-free DNA of stage IV colorectal cancer patients

**Figure 8:**
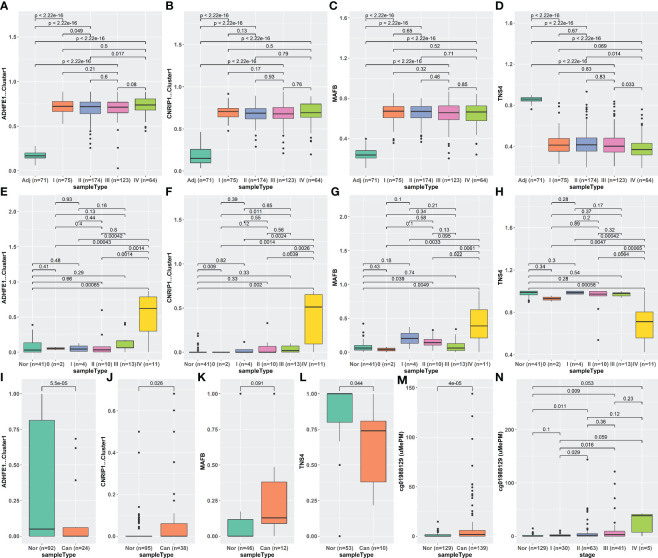
Methylation levels of iRFE MDGs of different stages in tissue and cfDNA. **(A–D)** Tissue DNA from TCGA-COAD dataset. **(E–H)** CfDNA from GSE149438 dataset. **(I–L)** CfDNA from CFEA dataset. **(M, N)** cfDNA from GSE124000 dataset. iRFE, iterative recursive feature elimination; MDGs, methylation-driven genes; cfDNA, cell-free DNA; CFEA, Cell-Free Epigenome Atlas.

Considering the classification performance of the iRFE LDA model for cancer and normal tissues, we expected to distinguish the cfDNA of CRC patients from healthy normal people by the LDA model constructed with COAD methylation data based on the four iRFE MDGs. Unexpectedly, however, although the methylation levels were significantly different between cfDNA in patients and healthy people (**Figures S15A–D**) in GSE149438, the efficiency of classification was not ideal (**Figure S15E**). To further explore the character of iRFE MDGs in cfDNA, we compared their methylation levels in cfDNA of healthy normal people and CRC patients with different stages. The methylation levels were not significantly different between normal people and stage 0–III patients (**Figures S15F–I, K–N**); the LDA model could not distinguish normal people from stage 0–III patients (**Figures S15J, O**). Only the cfDNA of stage IV patients could be distinguished from that of healthy normal people based on the methylation levels of iRFE MDGs (**Figures S15P–T**). These results indicated that, as emphasized in **Figures 8E–H**, the methylation levels of iRFE MDGs did not change until stage IV in cfDNA of CRC patients, although they had changed from stage I (or even from adenoma) in tissues (**Figures 8A–D**). To verify this result, we recruited another two datasets of cfDNA: the data of CRC patients and healthy normal people from Cell-Free Epigenome Atlas (CFEA, http://www.bio-data.cn/) (44) and GSE124600 (45). CFEA (http://www.bio-data.cn/) is a database containing methylation data of cell-free DNA in human diseases. We extracted the methylation data from 176 healthy normal people and 38 CRC patients, in which only a small part sample contained the methylation level data of all four MDGs. Dataset GSE124600 contained 129 samples from healthy normal people, 139 samples from CRC patients, and only one iRFE methylation site, cg01988129, a site in ADHFE1-Cluster1. The methylation levels of iRFE MDGs in cfDNA of cancer patients were significantly different from those of healthy people, as shown in **Figures 8I–L** (CFEA) and **Figure 8M** (GSE124600), which had a similar tendency to the result of GSE149438 (**Figures S15A–D**). When considering the tumor stage, the result of the data in GSE124600 (**Figure 8N**) had a similar tendency to the result of GSE149438 (**Figures 8E–H**), although the statistical significance was not identical, which might be influenced by the sample number.

## Discussion

In the present study, our primary aim was to screen the MDGs contributing to CRC carcinogenesis to deepen the understanding of this multi-stage process. Therefore, we screened 761 methylation clusters located at 752 CRC-related MDGs based on TCGA-COAD methylation and gene expression data by the *MethylMix* R package. Four methylation clusters, containing five methylation sites, located at four MDGs (*ADHFE1*, *CNRIP1*, *MAFB*, and *TNS4*) were screened by iRFE based on the LDA model and were used to construct a new LDA model (iRFE LDA model). The validation results based on independent cohorts indicated that the iRFE LDA model was robust and reliable. In addition, we attempted to distinguish CRC patients from healthy normal people based on cfDNA with the methylation levels of the four iRFE MDGs.

In previous studies, all four genes (ADHFE1, CNRIP1, MAFB, and TNS4) have been reported as cancer-related genes. *ADHFE1* gene encodes hydroxy acid-oxoacid transhydrogenase (EC 1.1.99.24), which is responsible for the oxidation of 4-hydroxybutyrate in mammalian tissues (46). *ADHFE1* has been linked with some cancers, including CRC (36, 47, 48). Fan et al. described the increased methylation level of *ADHFE1* in adenoma tissues compared with normal tissues (36), which is consistent with our results. In the present study, our results demonstrate that the methylation level of *ADHFE1* increases progressively in normal tissues and low- and high-grade adenoma tissues, while does not increase in cancer compared with adenoma, which suggests that the increased methylation level of *ADHFE1* plays a role only in the early stage of CRC carcinogenesis.

*CNRIP1* encodes a protein that interacts with the C-terminal tail of cannabinoid receptor 1 (CNR1), which can suppress CNR1-mediated tonic inhibition of voltage-gated calcium channels (49). Hypermethylation of *CNRIP1* has been observed in some cancer types. It is important to note that several studies have focused on the hypermethylation of a small panel of genes as a biomarker for colorectal cancer, including *CNRIP1* (50–52). Bodil Oster et al. reported that 15 selected genes are hypermethylated in adenomas and carcinomas, including *ADHFE1* and *CNRIP1* (50). In the present study, *ADHFE1* and *CNRIP1* have elevated methylation levels and decreased gene expression levels in adenoma and cancer compared with normal tissues, which is consistent with the results of Bodil Oster et al. This consistency suggests the reliability of our results.

The protein encoded by *MAFB* is a basic leucine zipper (bZIP) transcription factor that plays an important role in the regulation of lineage-specific hematopoiesis (53), which can repress ETS1-mediated transcription of erythroid-specific genes in myeloid cells (54). Correspondingly, *MAFB* is mainly linked with hematological tumors in previous studies. The only report about *MAFB* in CRC, for all we know, indicates that SUMOylated MAFB promotes CRC tumorigenesis through cell cycle regulation (55). The role of aberrant methylation of *MAFB* in carcinogenesis has not been reported. Therefore, we might discover a new methylation site (and a new gene perhaps) that participates in the early stage of colorectal carcinogenesis.

*TNS4* (tensin 4, also known as C-terminal tensin-like, Cten) is a member of the tensin protein family that comprises Tensin 1, Tensin 2, Tensin 3, and Cten/Tensin 4. Tensins 1–3 have extensive sequence and structural homology, including an actin-binding domain, a Src homology 2 (SH2) domain, and a phosphotyrosine binding (PTB) domain in common, which localizes to focal adhesion. TNS4 contains SH2 and PTB domains but lacks the actin-binding domain, which results in an inability to bind to the actin cytoskeleton and is thought to play a critical role in cellular processes such as cell motility (56). Correspondingly, *TNS4* plays an important role in the invasion and motility of cancer cells, including CRC (57–63). The only report about the role of *TNS4* in CRC carcinogenesis indicates that its upregulation promotes the tumorigenicity of colon cancer through β-catenin (64). Just like *MAFB*, no previous studies have investigated the roles of aberrant methylation of *TNS4* in colorectal carcinogenesis. The present study suggests that hypomethylation in *TNS4* is a new methylation site that played a role in the early stage of CRC carcinogenesis. Therefore, our study provides a foundation to explore the function of these two genes from methylation status. Apart from that, given the roles of *TNS4* in cell mobility and migration, the hypomethylation of this gene in adenoma and cancer tissues implies that colorectal cells might tend to migrate in the very early stage of carcinogenesis, rather than after malignant transformation.

Although all four MDGs have been reported in some cancers including CRC, the relationship of them, to our knowledge, has not been paid attention to. We have noticed the collinearity of the methylation levels of the four MDGs, which means that we can construct an LDA model based on three or fewer MDGs. Since the collinearity does not influence the classification performance, we did not try to remove any of them. In addition, considering that the functions of the four MDGs are not directly related, it is very interesting that their methylation levels are highly correlated with each other, especially in the situation where the methylation levels of the other MDGs are not correlated. This correlation suggests that there might be some underlying relationships among the four MDGs. Moreover, it might also imply that colorectal carcinogenesis is such a complicated process that it is impossible to be driven by a single gene or pathway. The results of single-gene GSEA indicate that the genes whose methylation levels are correlated with the four iRFE MDGs are enriched in the gene sets involved in the reprogramming process of a somatic cell to pluripotent stem cell (42) and are considered the common signatures of cancer cell and ES cell (43), which implies that the four MDGs promote colorectal carcinogenesis by participating in the dedifferentiation of the colorectal cell. These results may provide a direction for further experimental exploration of the roles of the aberrant methylation levels of the four MDGs in colorectal carcinogenesis.

Although the methylation levels of the four iRFE MDGs have been aberrant from the early stage of carcinogenesis in tissues, they do not change in cfDNA until stage IV. This contrast is confusing, especially considering the fact that several previous studies have constructed early diagnosis models based on the aberrant methylation in cfDNA (29, 65, 66). We acknowledge that this might be a deficiency of our model, which might be caused by several possible reasons:

1. CfDNA is released by apoptotic and necrotic cells (67), which dramatically rises in stage IV cancer tissues. As a result, although some genes have increased methylation levels in tumor tissues, few of them are released into the blood from tissue cells before stage IV. However, this reason cannot explain the fact that some methylated sites in cfDNA of CRC patients are specific and significantly different from those of healthy normal people (68, 69).
2. CfDNA is actively secreted in the form of exosomes or extracellular vesicles protected from blood degradation (70). DNA containing some methylated sites is secreted specifically, while the methylated sites in the present study are not released. However, the mechanism and meaning of this specific secretion of methylated genomic DNA are too inconceivable to imagine.
3. The clearance efficiency of cfDNA depends on various factors, including its association with molecular complexes that prevent rapid cfDNA degradation (71). In blood, cfDNA degradation is carried out essentially by circulating enzymes, such as DNase I, plasma factor VII-activating protease (FSAP), and factor H (72, 73), whose cleavage sites on DNA are not completely random. CfDNA with different binding proteins and sequences might be degraded with different efficiency, and the half-life of the cfDNA in the present study is too short to be detected in stages 0–III. A systematic analysis of the coverage of cfDNA on the whole genome-wide in CRC patients might be needed to evaluate the extent of degradation of cfDNA in different genomic regions in future studies. However, this reason cannot explain the increment in methylation level in cfDNA of stage IV patients and the biomarker of methylation sites in cfDNA for early diagnosis reported by previous studies.
4. The sample size of cfDNA is too small to detect the significant difference in methylation levels in cfDNA among patients with different stages.
5. The background lymphocytic DNA in plasma interferes with the detection of aberrant methylation levels. To interference, it might be necessary to apply other technologies, such as qRT-PCR or droplet digital PCR (ddPCR).
6. Considering that CRC is a typical model of multistage carcinogenesis, combining the Darwinian evolution and clonal successions, we supposed that the tumor tissue was almost certainly heterogeneous in many respects, including methylation. This might be a possible explanation for the difference in methylation changes in tumor tissue and cfDNA. A previous study indicated that a single cell-based method could clarify the heterogeneity and diversity of cancer cells in tumor tissue and blood (CTC) (74), which might help explore the heterogeneity of tumor tissue in future studies.

The results based on cfDNA indicate that the methylation levels of the four iRFE MDGs might be useful for monitoring the CRC progression, rather than the early diagnosis. This contrast highlighted the complexity of the relationship between cancer tissue DNA and cfDNA, which need to be further studied.

We acknowledge several other possible limitations in the present study:

1. While most of the results were validated with three independent datasets, the methylation levels of the four genes in low- and high-grade adenoma and cfDNA were observed in only one dataset (**Figures 5**, **8**, **S15**). Further validation with independent datasets is necessary.
2. The methylation levels of 202 samples are analyzed by HM27k methylation array, and 335 samples are analyzed by HM450k chip in the TCGA-COAD dataset. The methylation probes of HM27k and HM450k chips are intersected by the MethylMix algorithm to include more samples. However, this intersection discards most methylation probes on the HM450k chip. The 752 MDGs are screened from about 24k probes in the intersection of the HM27k and HM450k chips. MDG screening from the data produced by the HM450k chip may be necessary for more comprehensive knowledge about the roles of aberrant DNA methylation in CRC carcinogenesis.
3. Similar to the above, the whole-genome bisulfite sequencing (WGBS) and EPIC array cover more genomic regions than the HM450k array (75). Most of the validation sets used in this study are produced by HM450k array because large population data of WGBS or EPIC array are not available yet. MDG screening from the data produced by WGBS or EPIC may be necessary.
4. Although the LDA model based on the four genes suggests their roles in CRC carcinogenesis, the mechanism of their aberrant methylation of them needs to be further explored. These findings require further experimental validation in future studies.
5. None of the datasets recruited in this study mentioned that the samples were familial or sporadic. Since previous studies about the family history of malignancies indicated that CRC, even sporadic, was one of the most frequent malignancies (except for breast cancer) in the pedigrees of breast cancer patients (76, 77), the family history of sporadic CRC might be on the list of things worth studying.

## Conclusion

In conclusion, we identified the aberrantly methylated and differentially expressed MDGs with combined multi-omics analysis based on transcriptomic and DNA methylation profiles by the MethylMix algorithm, and we screened four genes to construct the LDA model, which was validated with independent datasets. Further studies indicated that methylation levels changed from the early stage of colorectal carcinogenesis. Correlation analysis and single-gene GSEA indicated that the four genes might promote CRC carcinogenesis by participating in dedifferentiation. However, the methylation levels of MDGs in cfDNA were not consistent with those in tissues, which indicated that it was inapplicable to early diagnosis of CRC. The functions and mechanism of the aberrant methylation of iRFE MDGs in colorectal carcinogenesis remain for further investigation. We hope these findings can provide theoretical references for further investigations. The viability period of cfDNA and the family history of CRC patients should be paid special attention to in future studies. Furthermore, the single-cell-based method might help to explore the heterogeneity of the cells in tumor tissues and the difference in the change in tumor tissues and cfDNA.

## Data availability statement

The original contributions presented in the study are included in the article/**Supplementary Material**. Further inquiries can be directed to the corresponding author.

## Author contributions

DYL: conception and design of the study, drafting of the manuscript, project administration, funding acquisition, and supervision. LZ: development of the analysis method, analysis and interpretation of data, drafting of the manuscript, and funding acquisition. CS: drafting and revision of the article. YuZ, YueZ, YJ, and XW: acquisition, analysis, and interpretation of the data. FL and DLL: interpretation of the data. SW and TY: acquisition of the data. JZ: revision of the article. All authors approved the submitted version.

## Funding

This work was supported by the Science and Technology Planning Project of Liaoning Province of China (grant number 2019-BS-002), the Science and Technology Planning Project of Liaoning Province of China (grant number 2021-MS-072), the National Natural Science Foundation of China (grant number 82002568), and the Science and Technology Planning Project of Shenyang (grant number 20-205-4-059).

## Conflict of interest

The authors declare that the research was conducted in the absence of any commercial or financial relationships that could be construed as a potential conflict of interest.

## Publisher’s note

All claims expressed in this article are solely those of the authors and do not necessarily represent those of their affiliated organizations, or those of the publisher, the editors and the reviewers. Any product that may be evaluated in this article, or claim that may be made by its manufacturer, is not guaranteed or endorsed by the publisher.
