# Supplementary figures and images for: Four methylation-driven genes detected by linear discriminant analysis model from early-stage colorectal cancer and their methylation levels in cell-free DNA
Source: Front Oncol. 2022 Sep 5;12:949244. doi: 10.3389/fonc.2022.949244 (PMC9491101; doi:10.3389/fonc.2022.949244)

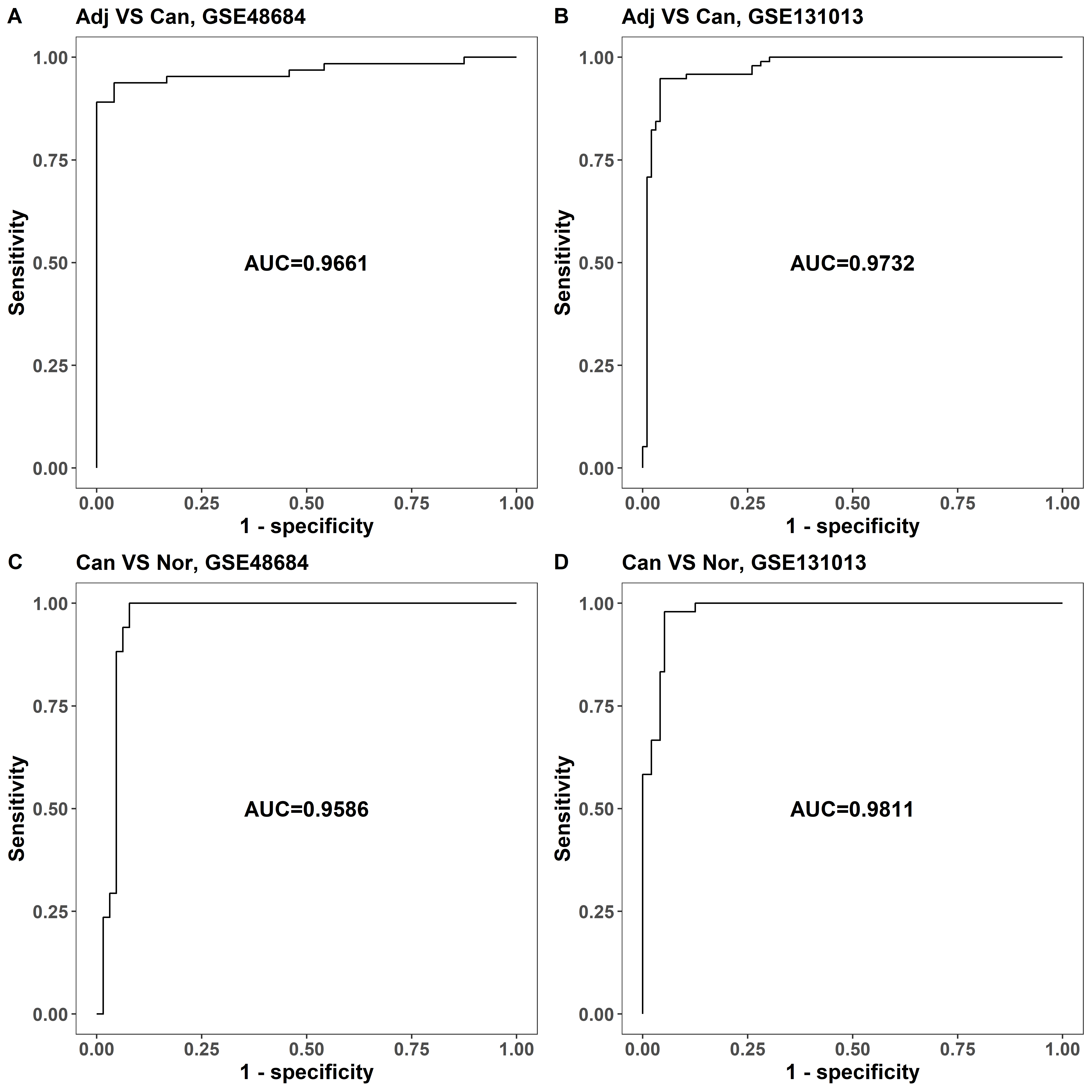

Supplement: Supplementary file 1 [file Image_1.png]

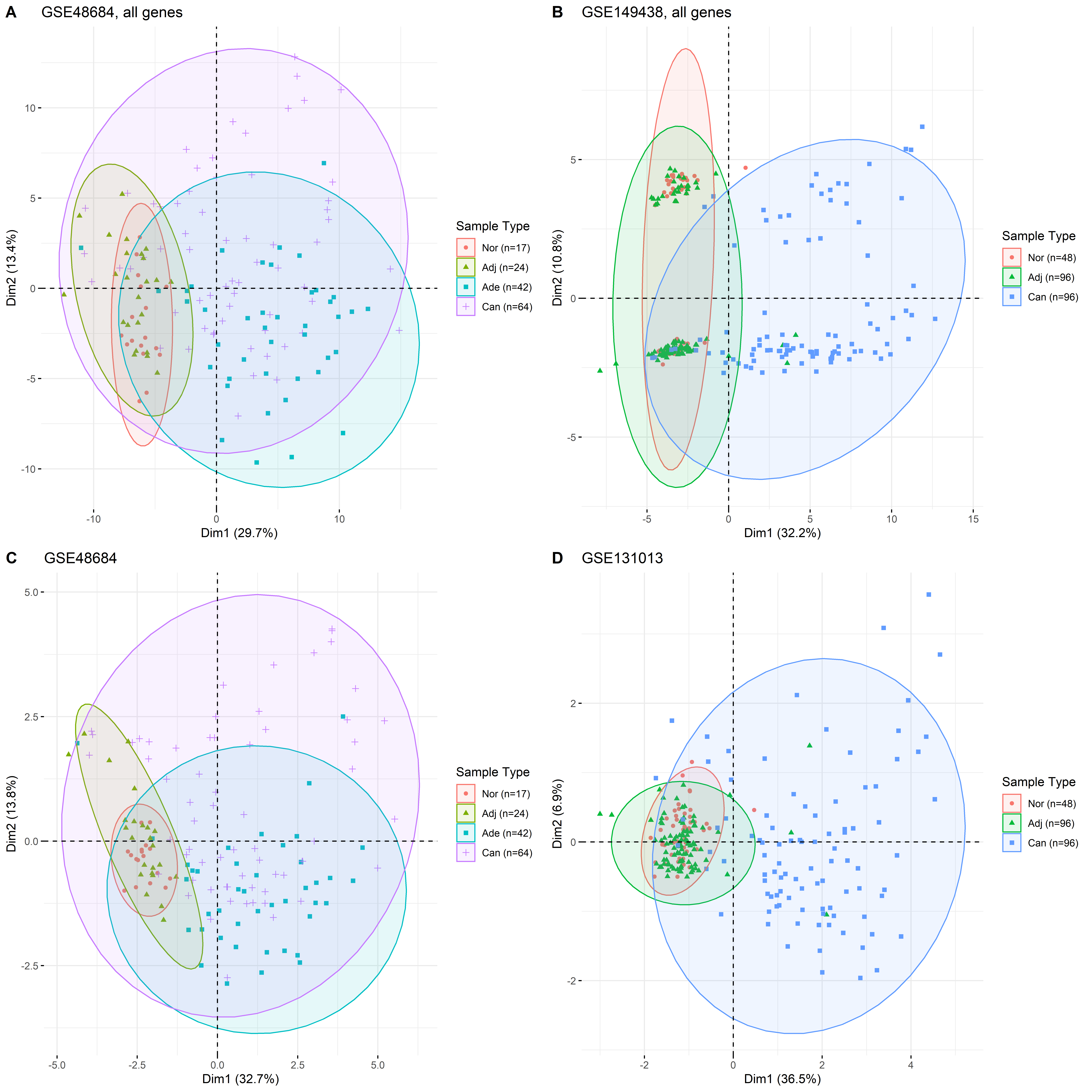

Supplement: Supplementary file 3 [file Image_3.png]

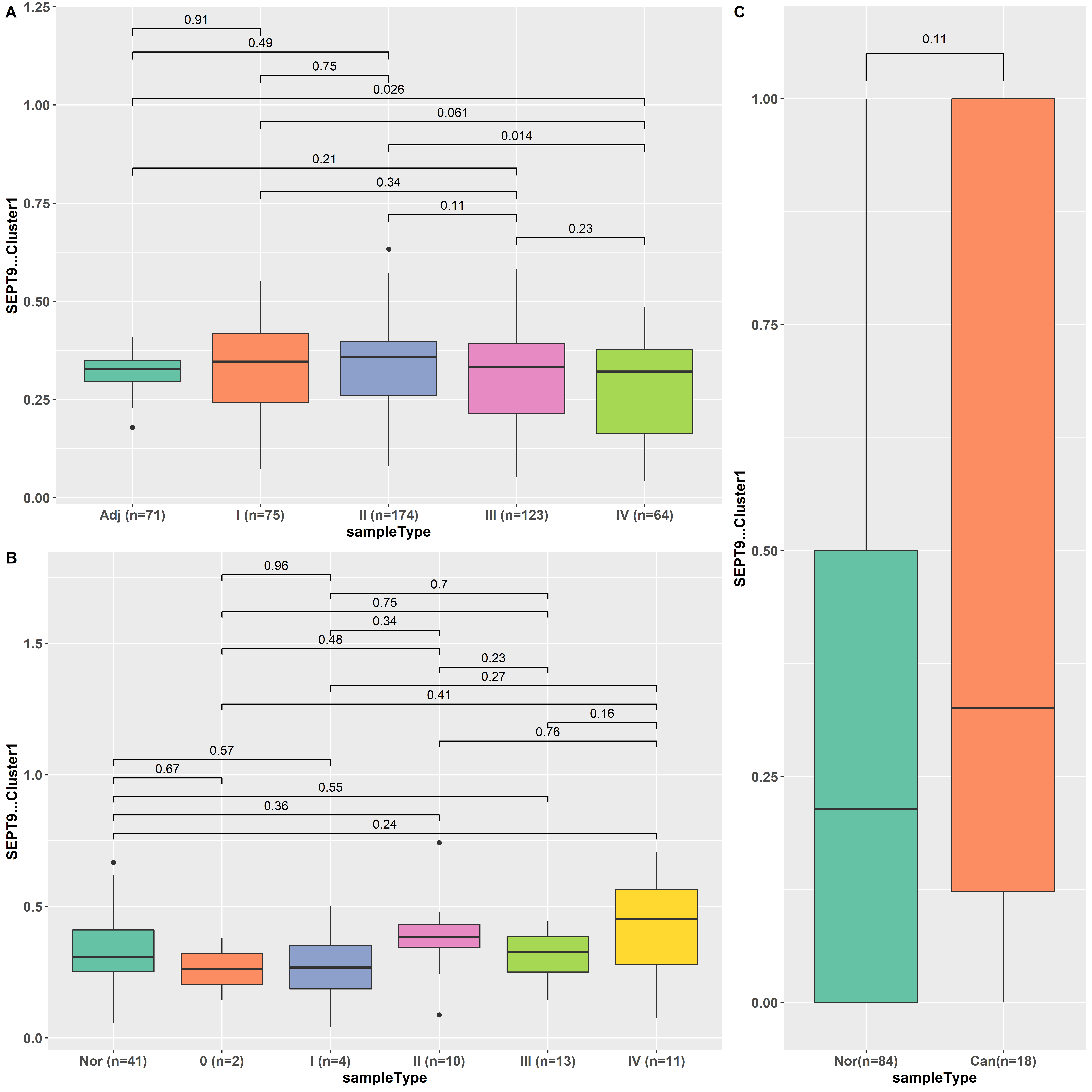

Supplement: Supplementary file 4 [file Image_4.png]

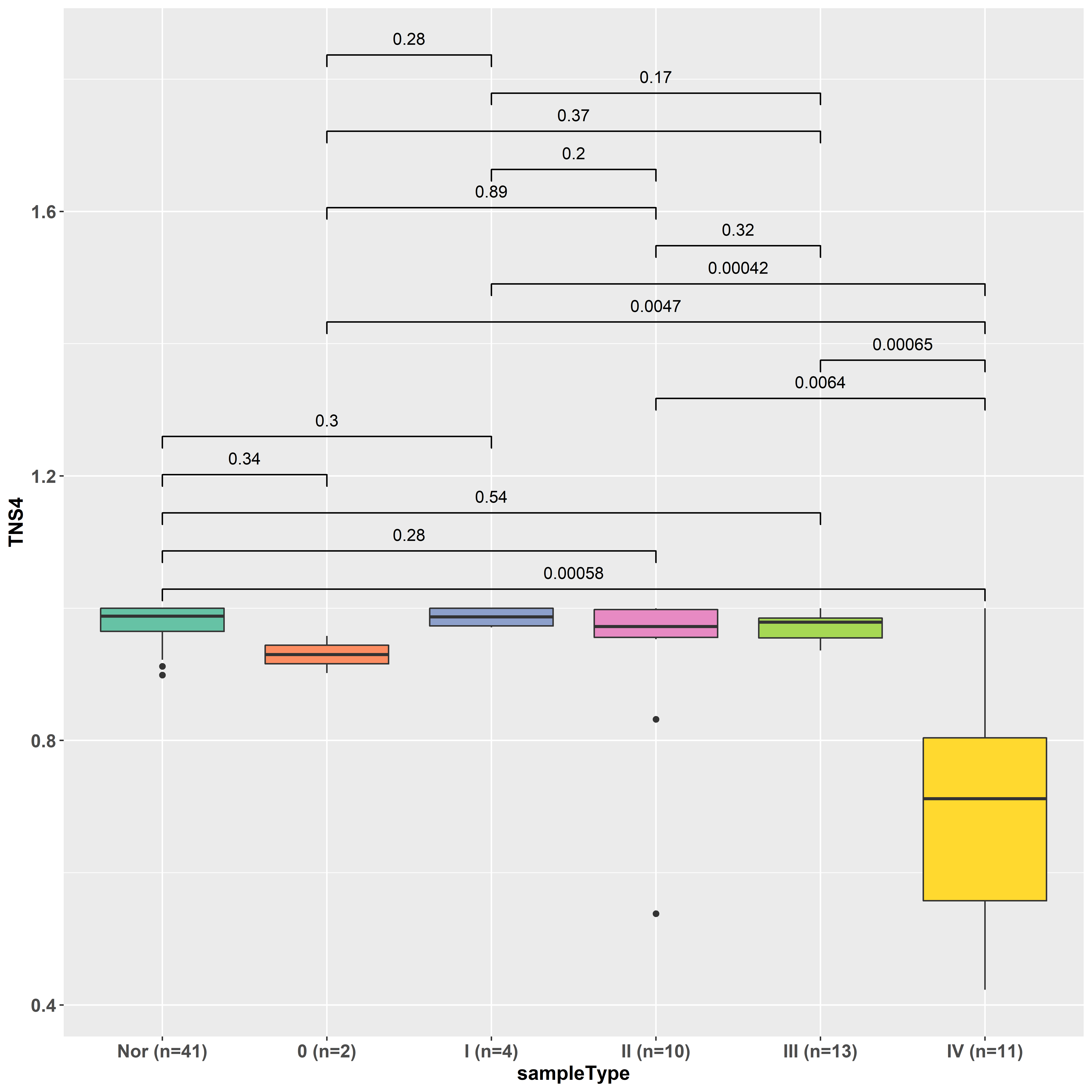

Supplement: Supplementary file 6 [file Image_6.png]

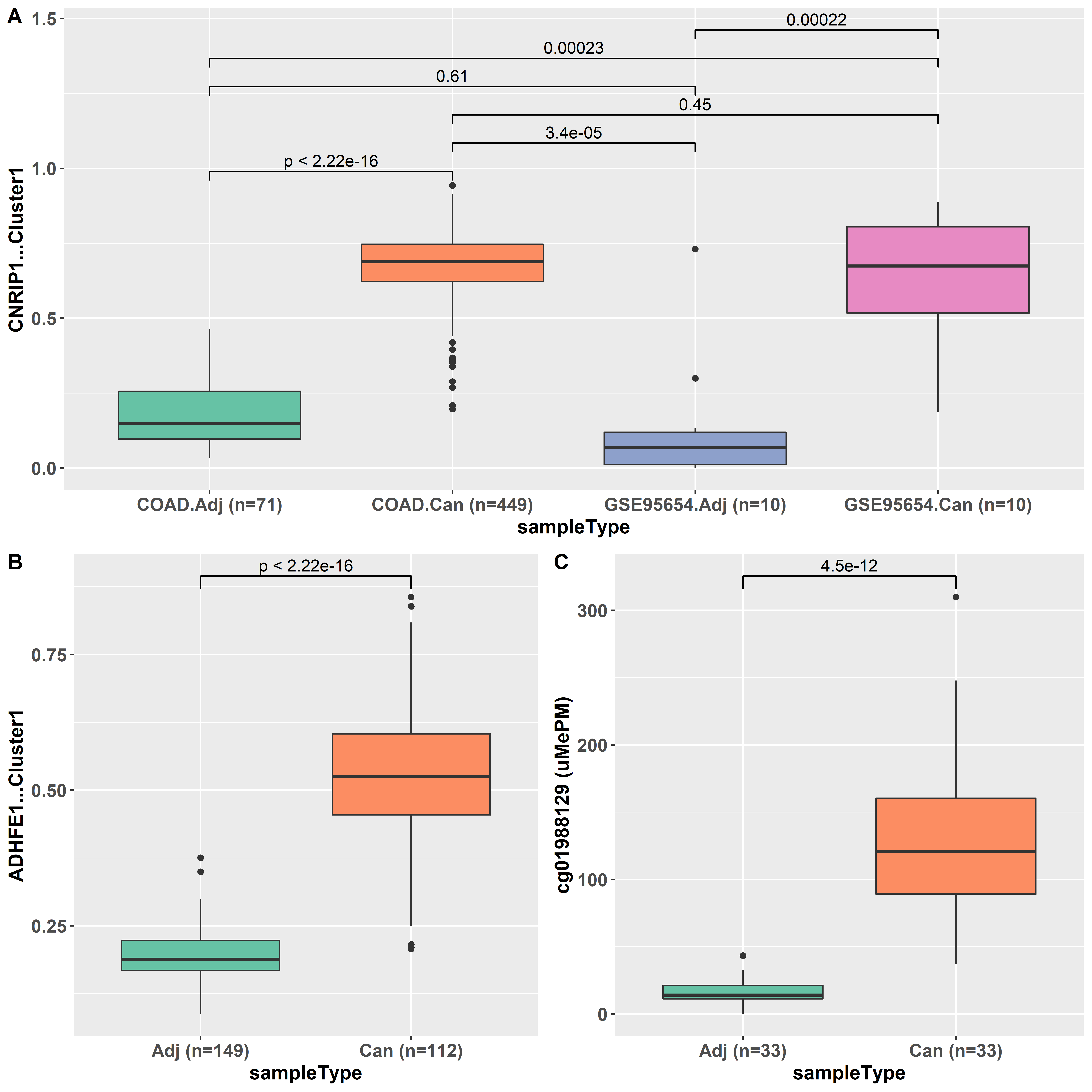

Supplement: Supplementary file 7 [file Image_7.png]

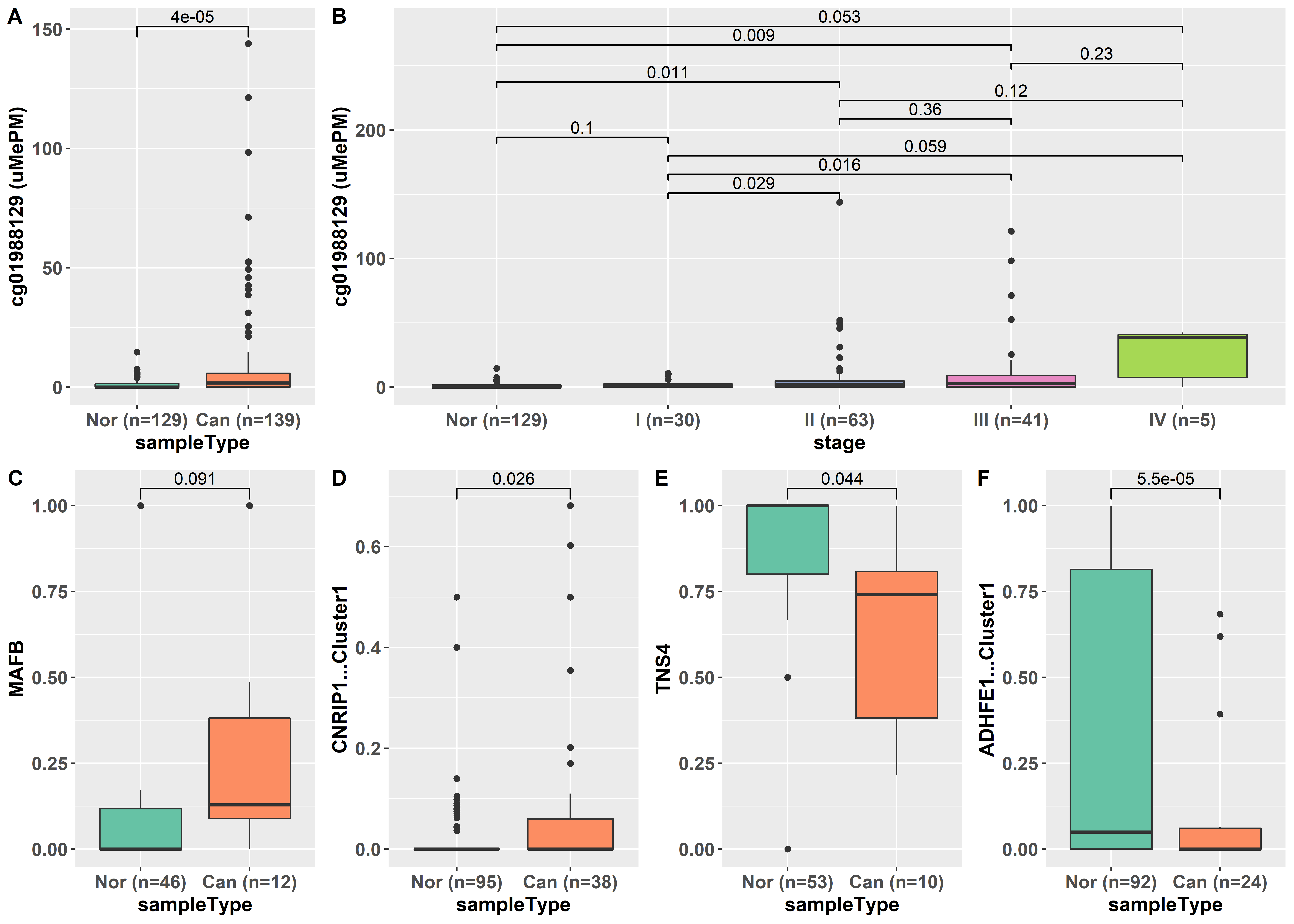

Supplement: Supplementary file 8 [file Image_8.png]
