# Supplementary material for: Four methylation-driven genes detected by linear discriminant analysis model from early-stage colorectal cancer and their methylation levels in cell-free DNA
Source: Front Oncol. 2022 Sep 5;12:949244. doi: 10.3389/fonc.2022.949244 (PMC9491101; doi:10.3389/fonc.2022.949244)
Supplement: Supplementary file 9 [file Presentation_1.pdf]

Four Methylation-driven Genes Detected by Linear Discriminant Analysis Model from Early-stage Colorectal Cancer and their methylation levels in cell-free DNA

Lei Zhan[1], Changjian Sun[2], Yu Zhang[2], Yue Zhang[2], Yuzhe Jia[2], Xiaoyan Wang[2], Feifei Li[1], Donglin Li[3], Shen Wang[4], Tao Yu[5], Jingdong Zhang[1], Deyang Li[2*]

1. Medical Oncology Department of Gastrointestinal Cancer, Liaoning Cancer Hospital & Institute, Cancer Hospital of China Medical University, Shenyang, Liaoning, 110042, China
2. Clinical lab, Air Force Hospital of Northern Theater, PLA, Shenyang, Liaoning, 110042, China
3. Orthopedics department, Air Force Hospital of Northern Theater, PLA, Shenyang, Liaoning, 110042, China
4. Department of Ultrasound and Special Diagnosis, Air Force Hospital of Northern Theater, PLA, Shenyang, Liaoning, 110042, China
5. Nursing Department, Air Force Medical Center, PLA, Beijing, 100142, China

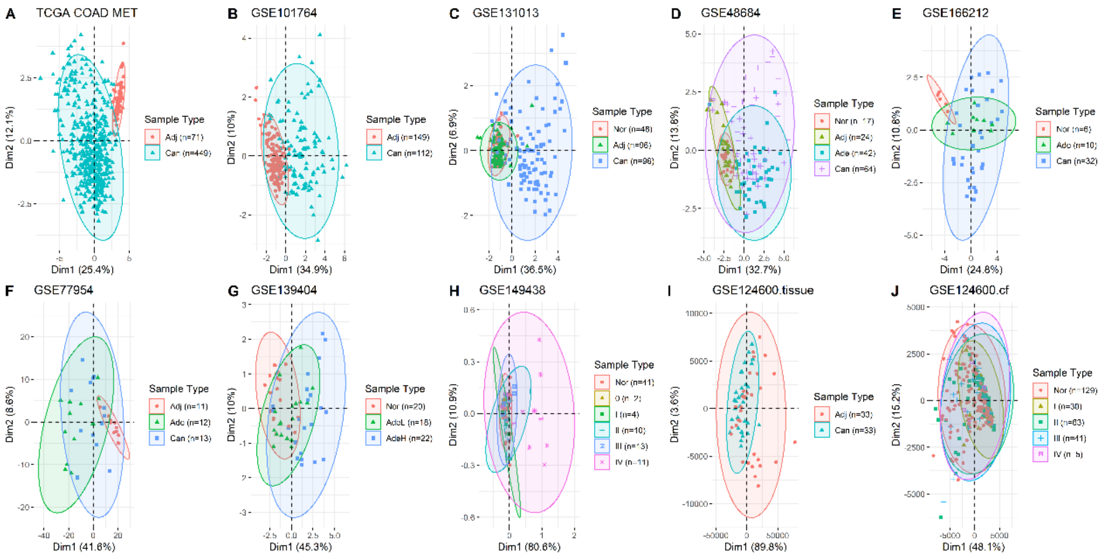

Figure S1. Principal component analysis (PCA) of methylation levels from 10 datasets. Nor, healthy normal tissues; Adj, cancer-adjacent tissues; Ade, adenoma tissues; AdeL, low-grade adenoma tissues; AdeH, high-grade adenoma tissues; Can, cancer tissues; 0-IV: stage 0-IV. There are too many NAs in CFEA to perform PCA.

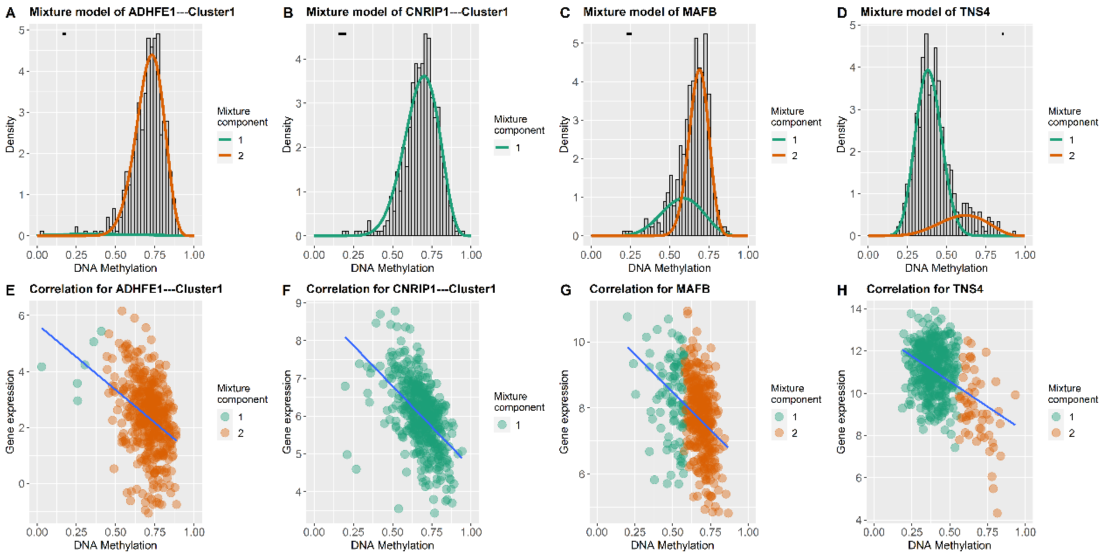

Figure S2. Overview of the 4 iRFE MDGs. **(A-D)** The β-mixture models of the 4 iRFE-MDGs. The x-axis indicates the methylation levels of samples from TCGA-COAD, the y-axis indicates the proportion at different levels, the curve indicates the peak value, and the black bar indicates the normal methylation levels. **(E-H)** Correlation between methylation and gene expression levels of the 4 iRFE MDGs.

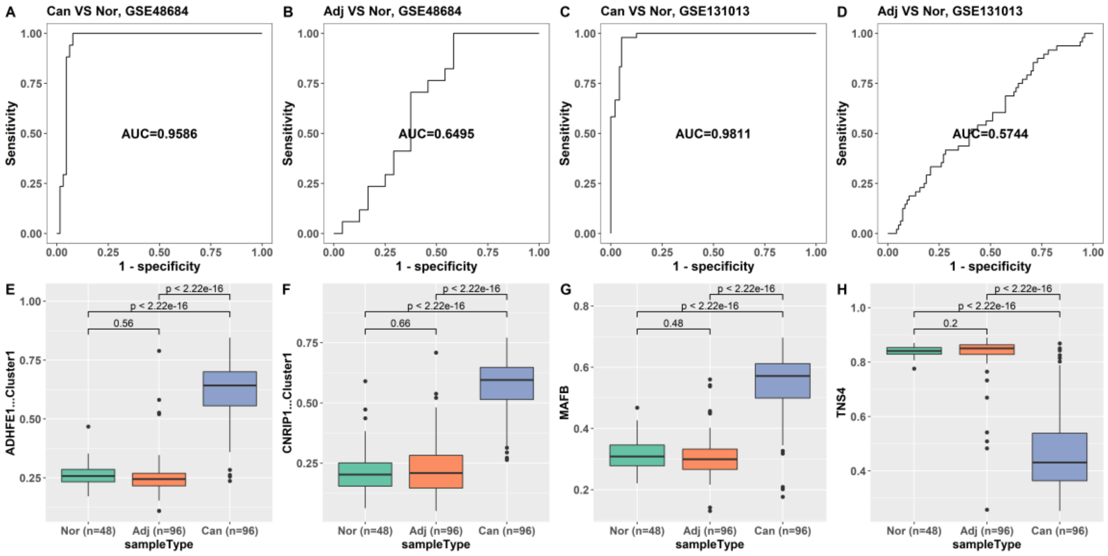

Figure S3. (A-D) Classification performance of iRFE LDA model for cancer and normal tissues **(A, C)** and cancer-adjacent and normal tissues **(B, D)** in GSE48684 and GSE131013 data sets. **(E-H)** Methylation levels of the 4 iRFE MDGCs in normal, cancer-adjacent, and cancer tissues in GSE131013 data sets. Nor, healthy normal tissues; Adj, cancer-adjacent tissues; Can, cancer tissues.

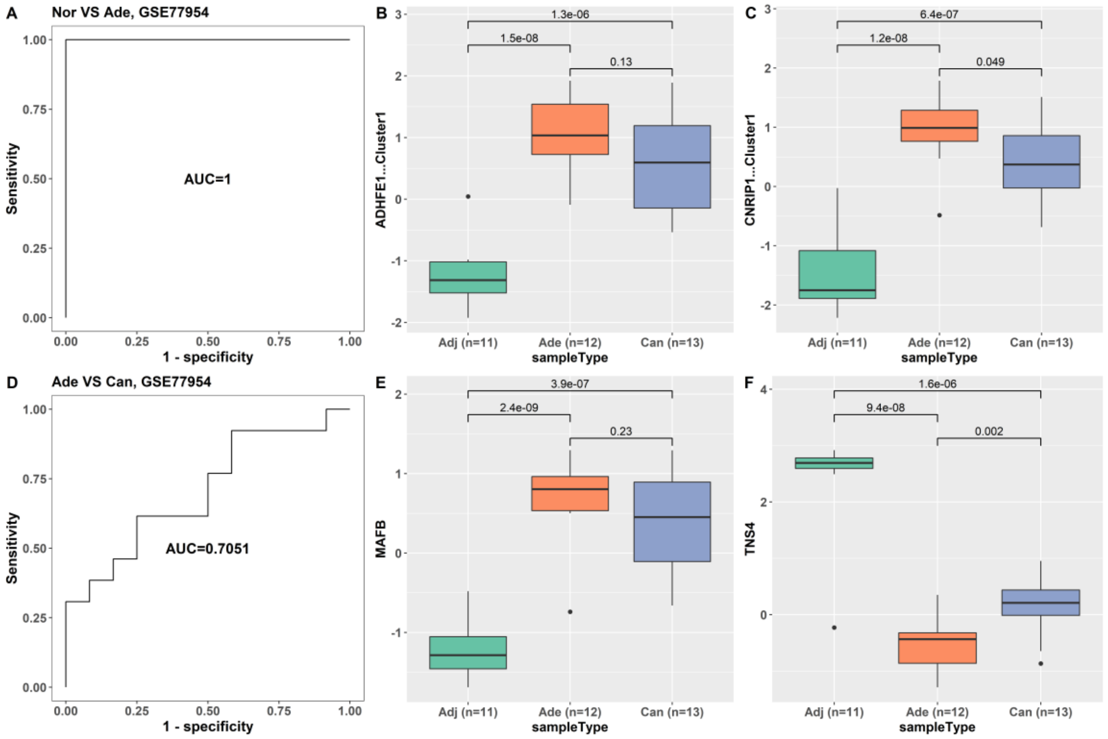

Figure S4. The classification ability and methylation levels of the 4 iRFE-MDGCs in GSE77954. **(A)** The classification performance of the LDA model for normal and adenoma samples; **(D)** The classification performance of the LDA model for adenoma and cancer tissues; **(B, C, E, F)** The methylation levels of the 4 iRFE-MDGs. **(B)** ADHFE1-Cluster1; **(C)** CNRIP1-Cluster1; **(E)** MAFB; **(F)** TNS4. Adj, cancer-adjacent tissues; Ade, adenoma tissues; Can, cancer tissues.

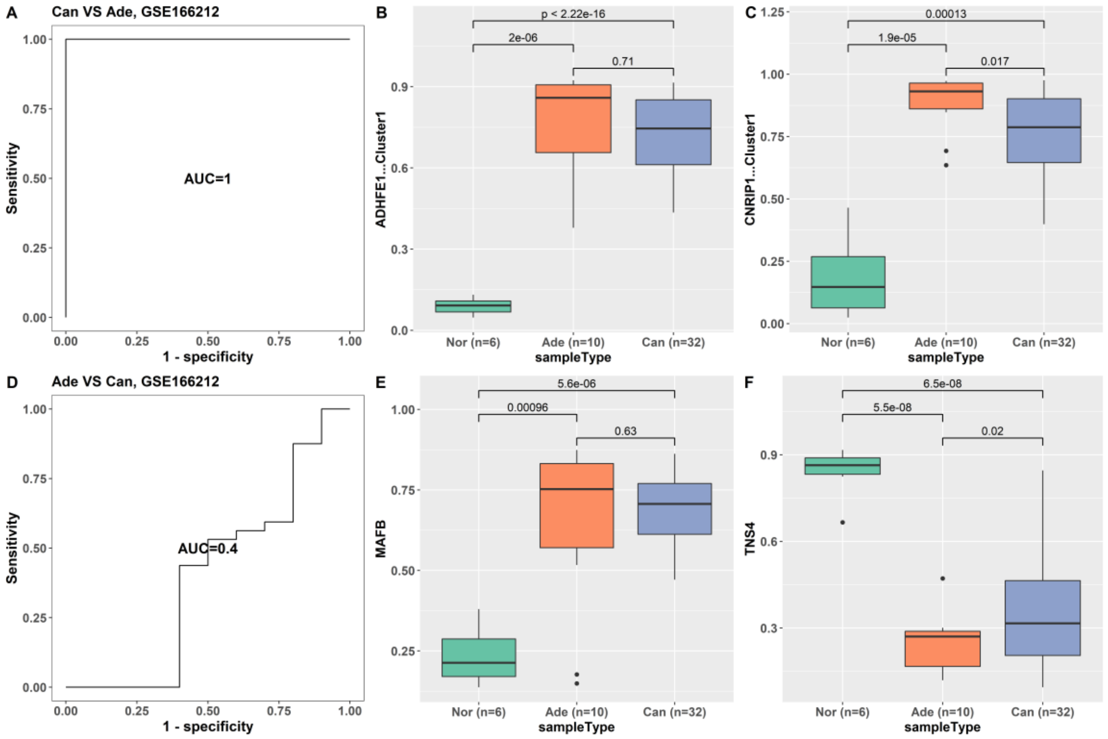

Figure S5. The classification ability and methylation levels of the 4 iRFE-MDGCs in GSE166212. **(A)** The classification performance of the LDA model for normal and adenoma samples; **(D)** The classification performance of the LDA model for adenoma and cancer tissues; **(B, C, E, F)** The methylation levels of the 4 iRFE-MDGs. **(B)** ADHFE1-Cluster1; **(C)** CNRIP1-Cluster1; **(E)** MAFB; **(F)** TNS4. Nor, healthy normal tissues; Ade, adenoma tissues; Can, cancer tissues.

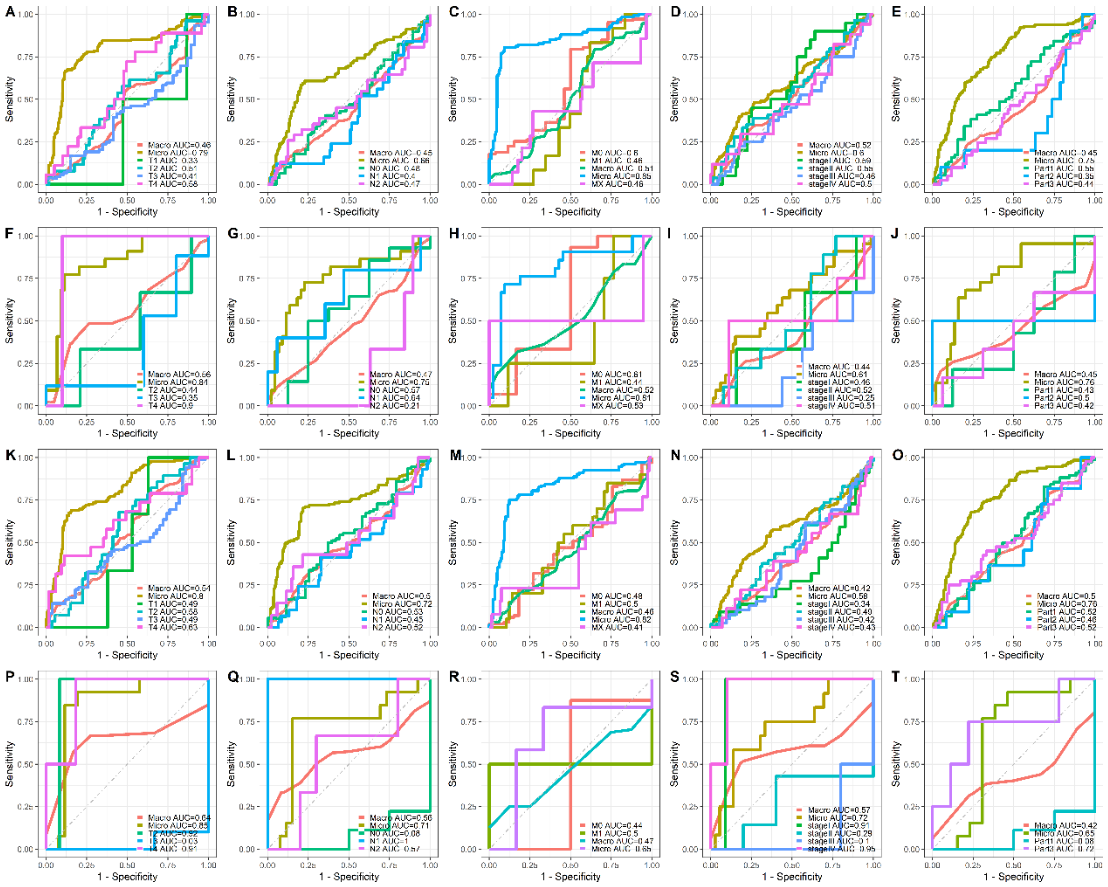

Figure S6. Classification of clinical phenotypes with LDA models based on 4 iRFE MDGs. (A-E) methylation data in cancer; **(F-J)** methylation data in normal; **(K-O)** gene expression data in cancer; **(P-T)** gene expression data in normal; **(A, F, K, P)** T stage; **(B, G, L, Q)** N stage; **(C, H, M, R)** M stage; **(D, I, N, S)** TNM stage; **(E, J, O, T)** number of lymph nodes positive by HE.

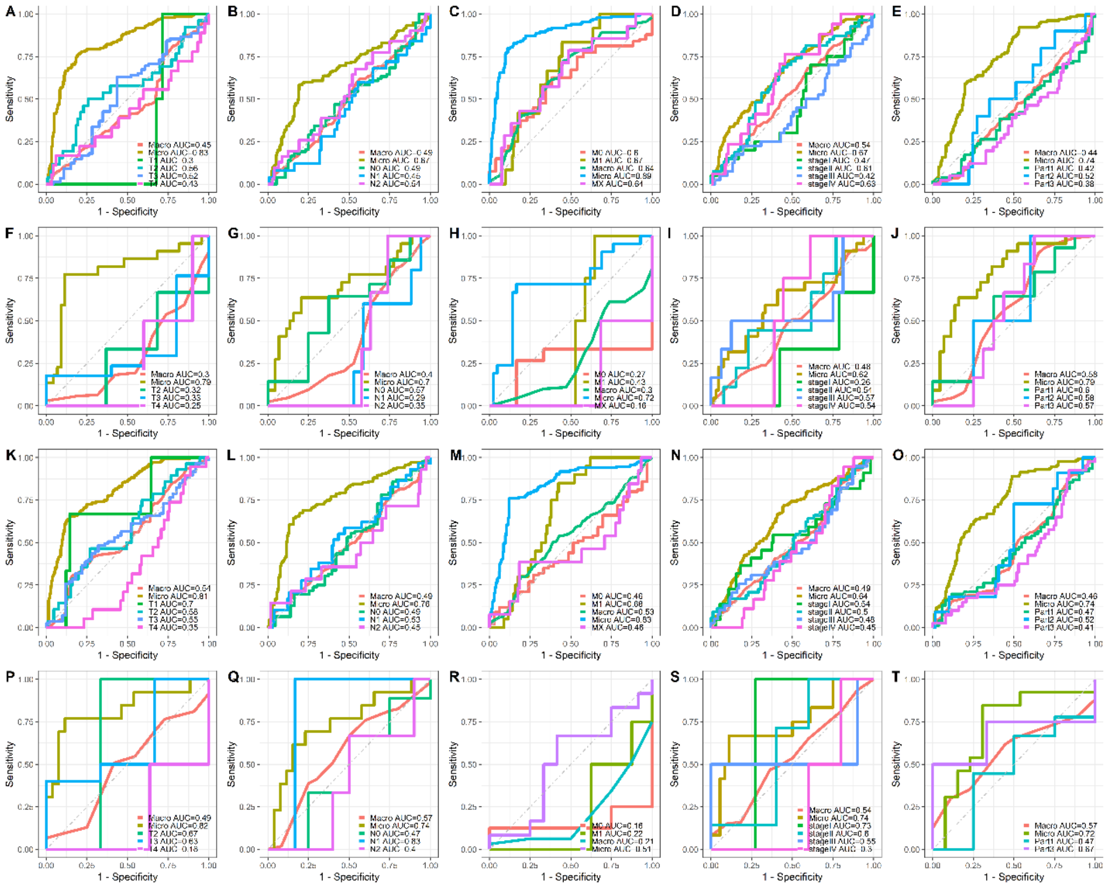

Figure S7. Classification of clinical phenotypes with KNN models based on 4 iRFE MDGs. (A-E) methylation data in cancer; **(F-J)** methylation data in normal; **(K-O)** gene expression data in cancer; **(P-T)** gene expression data in normal; **(A, F, K, P)** T stage; **(B, G, L, Q)** N stage; **(C, H, M, R)** M stage; **(D, I, N, S)** TNM stage; **(E, J, O, T)** number of lymph nodes positive by HE.

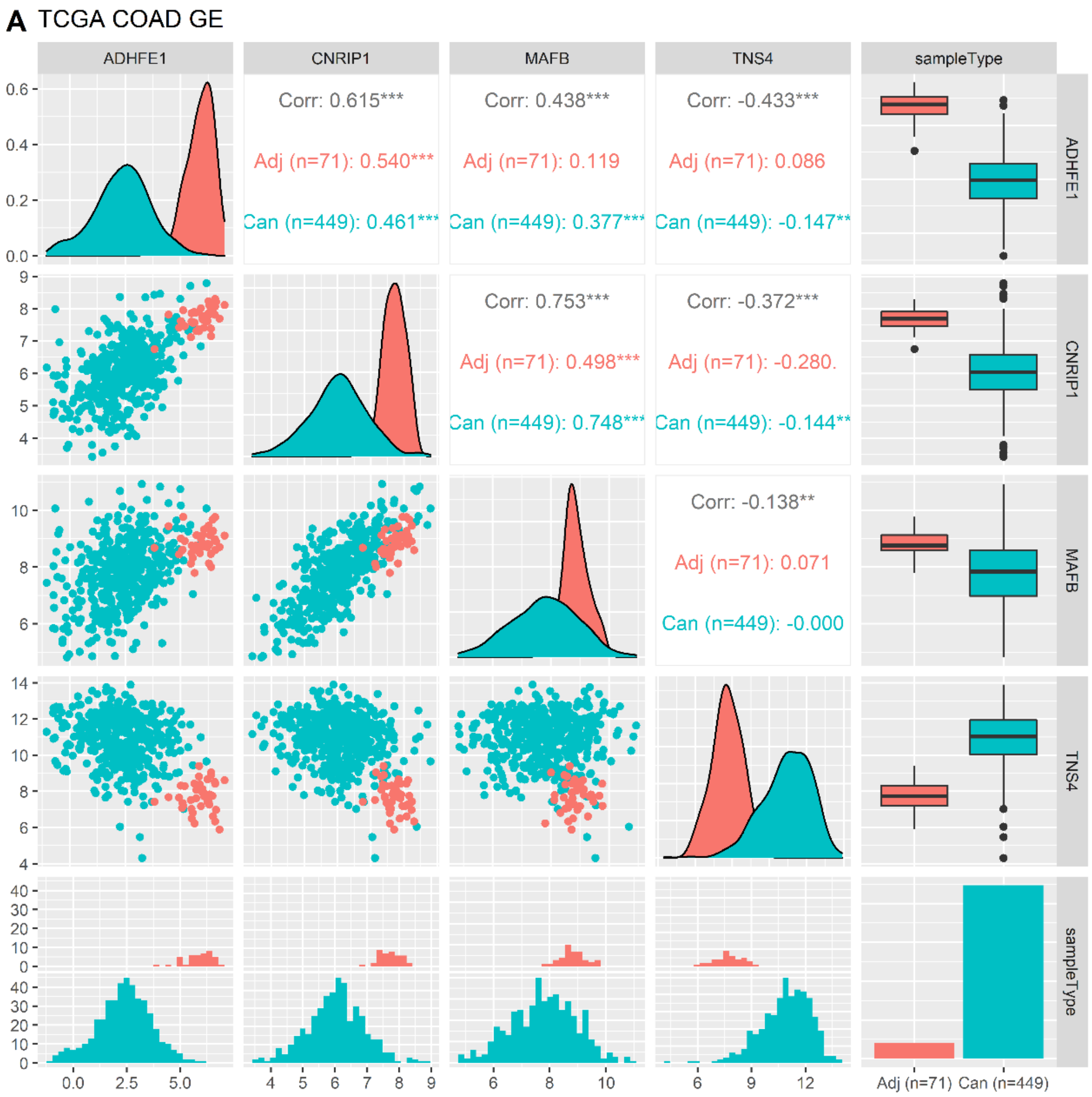

Figure S8. Correlation analysis of gene expression levels of the 4 iRFE MDGs in TCGA-COAD dataset. **: P < 0.01; ***: P < 0.001.

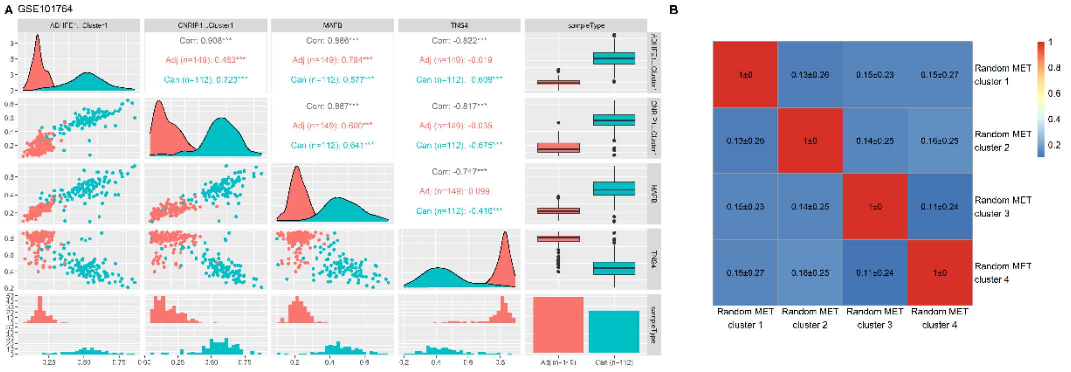

Figure S9. Correlation analysis of the methylation levels of the 4 iRFE MDGCs and randomly selected MDGCs in GSE101764. **(A)** Correlation plots and distribution of the methylation levels of the 4 iRFE MDGs; **(B)** Averaged correlation coefficients of methylation levels of randomly selected MDGCs (100 repeats). ***: P < 0.001.

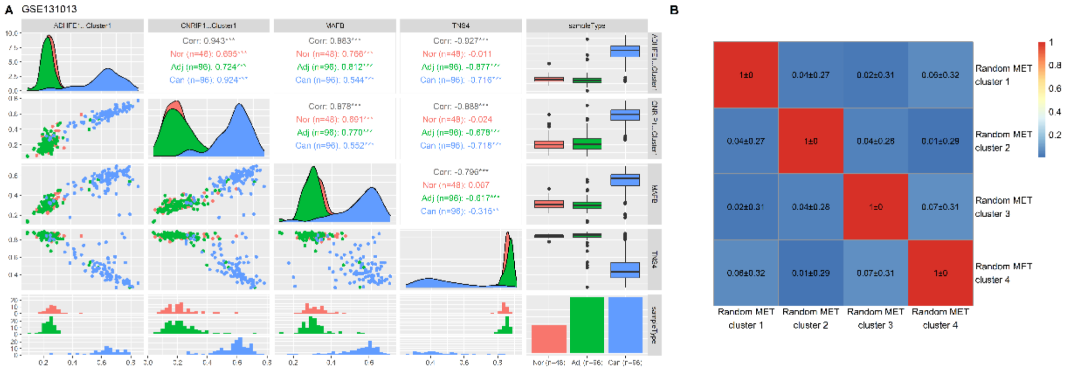

Figure S10. Correlation analysis of the methylation levels of the 4 iRFE MDGCs and randomly selected MDGCs in GSE131013. **(A)** Correlation plots and distribution of the methylation levels of the 4 iRFE MDGs; **(B)** Averaged correlation coefficients of methylation levels of randomly selected MDGCs (100 repeats). **: P < 0.01; ***: P < 0.001.

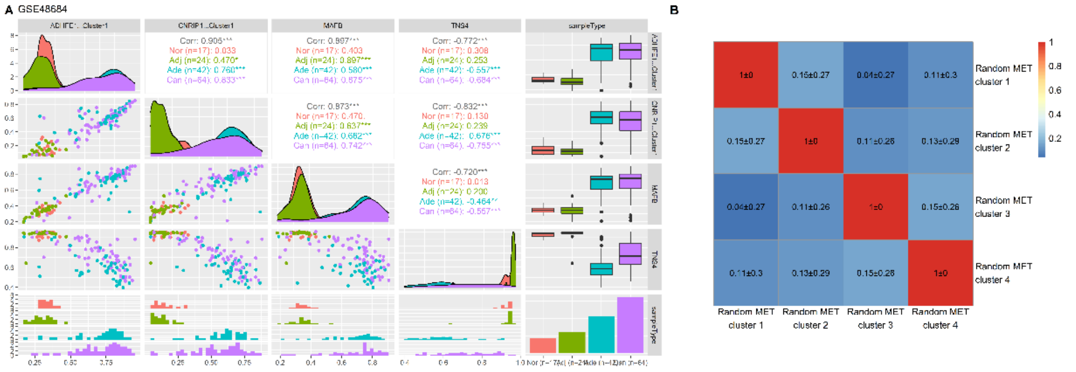

Figure S11. Correlation analysis of the methylation levels of the 4 iRFE MDGCs and randomly selected MDGCs in GSE48684. **(A)** Correlation plots and distribution of the methylation levels of the 4 iRFE MDGs; **(B)** Averaged correlation coefficients of methylation levels of randomly selected MDGCs (100 repeats). **: $P < 0.01$; ***: $P < 0.001$.

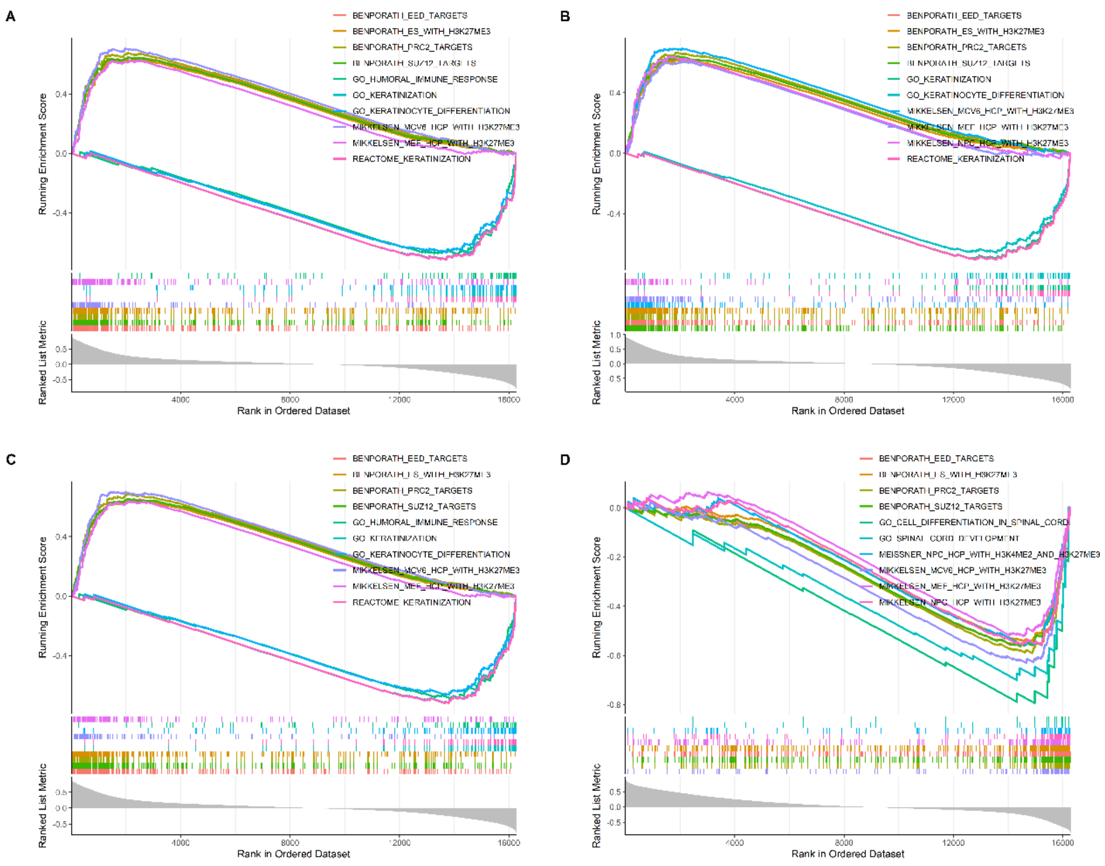

Figure S12. Single gene GSEA results based on the correlation coefficients between the methylation levels of the 4 iRFE MDGs and that of other genes in GSE101764. The gene sets with the top 10 NES values were shown. **(A)** *ADHFE1*; **(B)** *CNRIP1*; **(C)** *MAFB*; **(D)** *TNS4*. NES, normalized enrichment score.

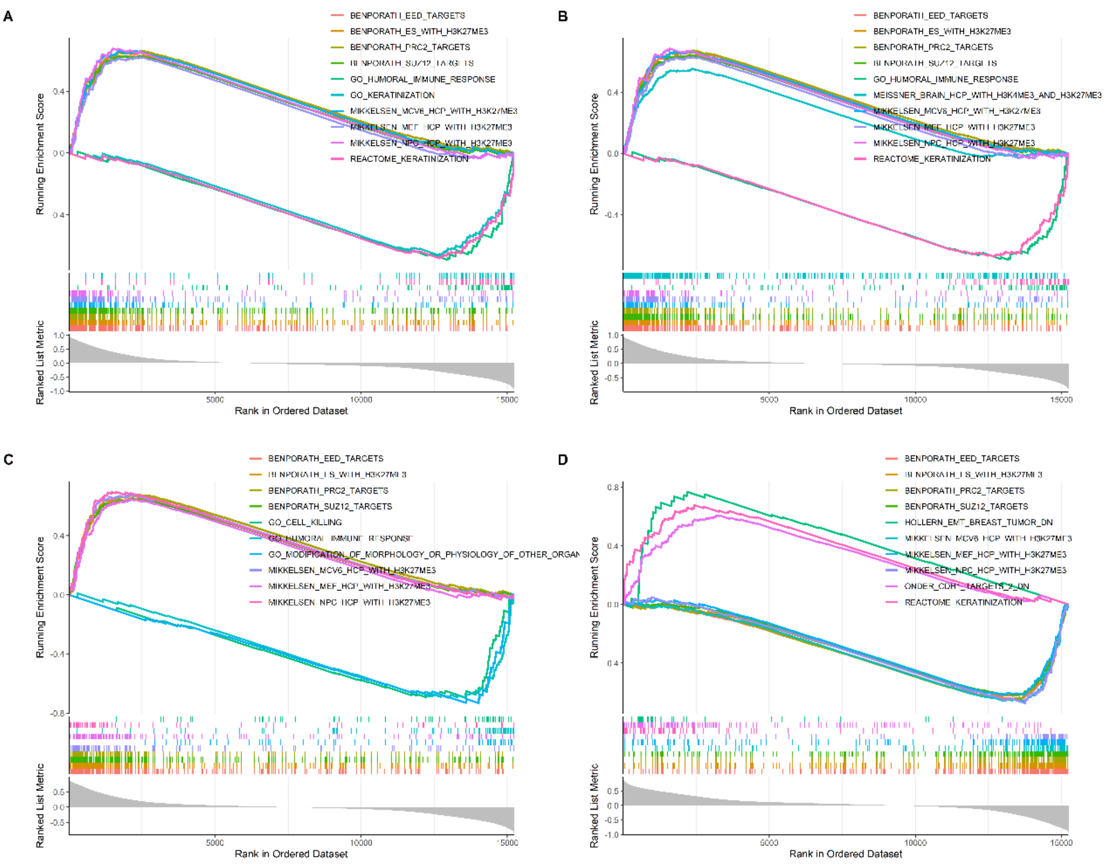

Figure S13. Single gene GSEA results based on the correlation coefficients between the methylation levels of the 4 iRFE MDGs and that of other genes in GSE131013. The gene sets with the top 10 NES values were shown. **(A)** *ADHFE1*; **(B)** *CNRIP1*; **(C)** *MAFB*; **(D)** *TNS4*. NES, normalized enrichment score.

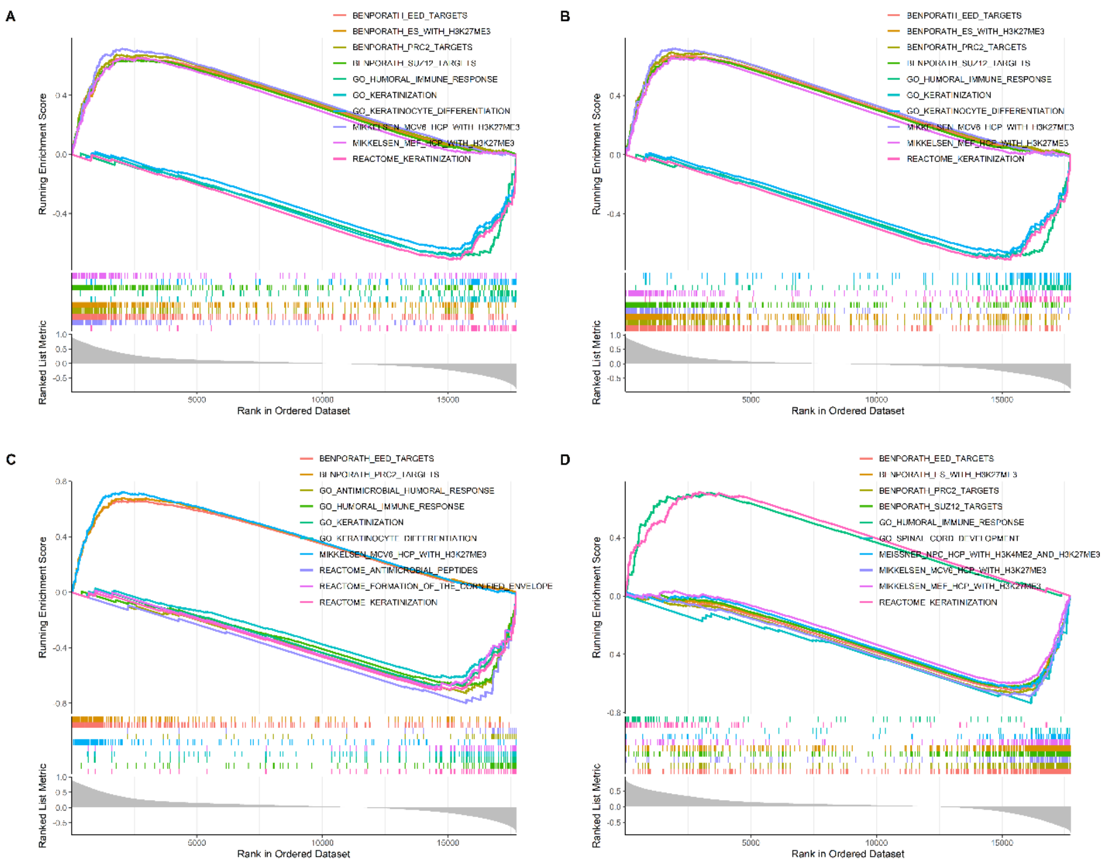

Figure S14. Single gene GSEA results based on the correlation coefficients between the methylation levels of the 4 iRFE MDGs and that of other genes in GSE48684. The gene sets with the top 10 NES values were shown. **(A)** *ADHFE1*; **(B)** *CNRIP1*; **(C)** *MAFB*; **(D)** *TNS4*. NES, normalized enrichment score.

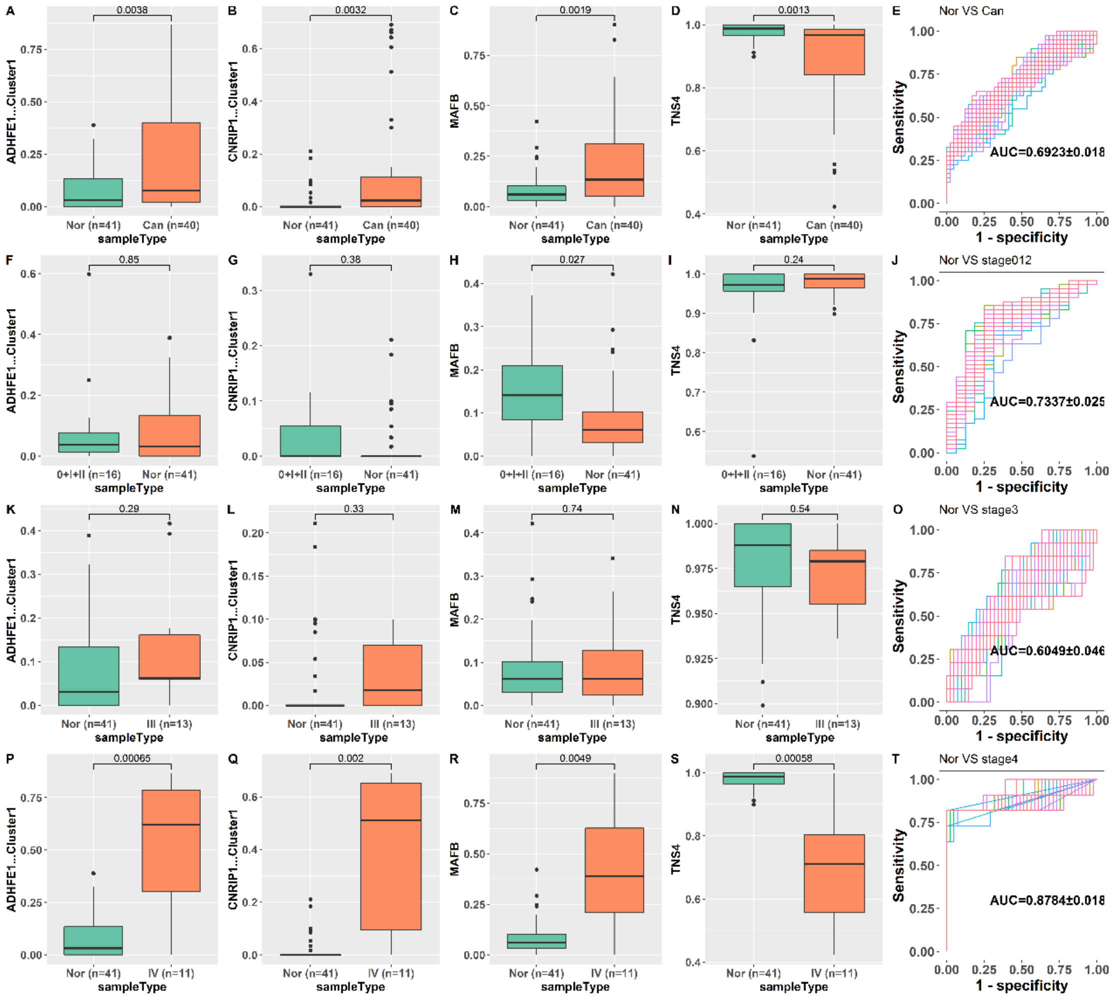

Figure S15. Methylation levels and classification performance of iRFE MDGs in cfDNA samples. (A-E) Normal people VS. all cancer patients; (F-J) Normal people VS. stage 0, I, II patients; (K-O) Normal people VS. stage III patients; (P-T) Normal people VS. stage IV patients.

Table S3 Prognostic analysis with methylation data in cancer

	coef	exp (coef)	se (coef)	Z	p
**ADHFE1---Cluster1**	0.74	2.10	1.35	0.55	0.58
**CNRIP1---Cluster1**	0.19	1.21	1.22	0.16	0.88
**MAFB**	0.66	1.94	1.23	0.54	0.59
**TNS4**	0.99	2.68	0.94	1.05	0.29

Likelihood ratio test=1.57 on 4 df, p=0.8139, n= 425, number of events= 92.

Table S4 Prognostic analysis with methylation data in normal

	coef	exp (coef)	se (coef)	z	p
**ADHFE1---Cluster1**	6.63	755.50	10.92	0.61	0.54
**CNRIP1---Cluster1**	5.89	360.40	3.52	1.67	0.09
**MAFB**	5.57	0.00	8.21	0.68	0.50
**TNS4**	13.34	624400.00	13.32	1.00	0.32

Likelihood ratio test=3.24 on 4 df, p=0.518, n= 63, number of events= 14.

Table S5 Prognostic analysis with gene expression data in cancer

	coef	exp (coef)	se (coef)	z	p
**ADHFE1**	0.07	1.08	0.09	0.78	0.44
**CNRIP1**	0.25	0.78	0.19	1.31	0.19
**MAFB**	0.26	1.29	0.14	1.84	0.07
**TNS4**	0.05	0.96	0.08	0.59	0.55

Likelihood ratio test=4.25 on 4 df, p=0.3738, n= 427, number of events= 93.

Table S6 Prognostic analysis with gene expression data in normal

	coef	exp (coef)	se (coef)	z	p
**ADHFE1**	0.37	1.45	0.63	0.60	0.55
**CNRIP1**	0.23	1.26	1.76	0.13	0.90
**MAFB**	0.31	1.37	0.90	0.35	0.73
**TNS4**	0.07	0.93	0.47	0.15	0.88

Likelihood ratio test=1 on 4 df, p=0.9098, n= 39, number of events= 12.
