# Supplementary material for: Four methylation-driven genes detected by linear discriminant analysis model from early-stage colorectal cancer and their methylation levels in cell-free DNA
Source: Front Oncol. 2022 Sep 5;12:949244. doi: 10.3389/fonc.2022.949244 (PMC9491101; doi:10.3389/fonc.2022.949244)
Supplement: Supplementary file 10 [file Table_1.docx]

**Table S1 the Information of MDGCs**

| **Probe ID** | **Methylation Cluster** | **Gene Symbol** |  |
| --- | --- | --- | --- |
| cg17044311 | ABCC2---Cluster2 | *ABCC2* |  |
| cg08145177 | ABHD8---Cluster1 | ***ABHD8*** |  |
| cg22734480 | ABHD8---Cluster2 | ***ABHD8*** |  |
| cg08559914 | ACE2 | *ACE2* |  |
| cg26780333 | ACOT4 | *ACOT4* |  |
| cg25683185 | ACRBP | *ACRBP* |  |
| cg13849691 | ACSL5 | *ACSL5* |  |
| cg19986872 | ACSL6---Cluster2 | *ACSL6* |  |
| cg00354572 | ***ACSS3---Cluster1*** | ***ACSS3*** |  |
| cg01283289 | ***ACSS3---Cluster1*** | ***ACSS3*** |  |
| cg23640701 | ACVRL1 | *ACVRL1* |  |
| cg26918442 | ***ADAL---Cluster1*** | ***ADAL*** |  |
| cg27015174 | ***ADAL---Cluster1*** | ***ADAL*** |  |
| cg02564733 | ADAMTS15---Cluster1 | *ADAMTS15* |  |
| cg08016383 | ***ADAMTS17---Cluster1*** | ***ADAMTS17*** |  |
| cg10135717 | ***ADAMTS17---Cluster1*** | ***ADAMTS17*** |  |
| cg07651242 | ***ADCY1---Cluster1*** | ***ADCY1*** |  |
| cg13523557 | ***ADCY1---Cluster1*** | ***ADCY1*** |  |
| cg07376535 | ADCYAP1 | *ADCYAP1* |  |
| cg01988129 | ***ADHFE1---Cluster1*** | ***ADHFE1*** |  |
| cg08090772 | ***ADHFE1---Cluster1*** | ***ADHFE1*** |  |
| cg26926521 | ADRA2A---Cluster2 | *ADRA2A* |  |
| cg24113449 | ADRBK2 | *ADRBK2* |  |
| cg07640820 | AGK | *AGK* |  |
| cg07331806 | AIFM3 | *AIFM3* |  |
| cg11003133 | AIM2 | *AIM2* |  |
| cg17525406 | ***AJAP1---Cluster1*** | ***AJAP1*** |  |
| cg20959866 | ***AJAP1---Cluster1*** | ***AJAP1*** |  |
| cg10482024 | ***AK3L1---Cluster1*** | ***AK3L1*** |  |
| cg20802051 | ***AK3L1---Cluster1*** | ***AK3L1*** |  |
| cg13801416 | ***AKR1B1---Cluster1*** | ***AKR1B1*** |  |
| cg18416881 | ***AKR1B1---Cluster1*** | ***AKR1B1*** |  |
| cg09272256 | AKR1C4 | *AKR1C4* |  |
| cg11376198 | ***AKR7L---Cluster1*** | ***AKR7L*** |  |
| cg23625458 | ***AKR7L---Cluster1*** | ***AKR7L*** |  |
| cg07800320 | ***ALDH1L1---Cluster1*** | ***ALDH1L1*** |  |
| cg16312163 | ***ALDH1L1---Cluster1*** | ***ALDH1L1*** |  |
| cg17838765 | ***ALKBH3---Cluster1*** | ***ALKBH3*** |  |
| cg22637507 | ***ALKBH3---Cluster1*** | ***ALKBH3*** |  |
| cg18374517 | ALPPL2---Cluster2 | *ALPPL2* |  |
| cg06630567 | ***AMBP---Cluster1*** | ***AMBP*** |  |
| cg25420952 | ***AMBP---Cluster1*** | ***AMBP*** |  |
| cg26782833 | AMMECR1L---Cluster2 | *AMMECR1L* |  |
| cg20191453 | ***AMT---Cluster1*** | ***AMT*** |  |
| cg25021247 | ***AMT---Cluster1*** | ***AMT*** |  |
| cg05985767 | ANPEP---Cluster1 | *ANPEP* |  |
| cg22710329 | ANUBL1 | *ANUBL1* |  |
| cg00283535 | ANXA13 | *ANXA13* |  |
| cg02144933 | ***AOX1---Cluster1*** | ***AOX1*** |  |
| cg12627583 | ***AOX1---Cluster1*** | ***AOX1*** |  |
| cg19264571 | ***APCDD1---Cluster1*** | ***APCDD1*** |  |
| cg26328002 | ***APCDD1---Cluster1*** | ***APCDD1*** |  |
| cg17207590 | APH1B---Cluster2 | *APH1B* |  |
| cg07186138 | APOBEC3C | *APOBEC3C* |  |
| cg13119609 | APOC2---Cluster1 | *APOC2* |  |
| cg02813863 | ***APOLD1---Cluster1*** | ***APOLD1*** |  |
| cg26244225 | ***APOLD1---Cluster1*** | ***APOLD1*** |  |
| cg04551925 | AQP1---Cluster1 | *AQP1* |  |
| cg23855989 | AQP5---Cluster2 | *AQP5* |  |
| cg11136562 | ARAP3 | *ARAP3* |  |
| cg10925082 | ***ARHGDIB---Cluster1*** | ***ARHGDIB*** |  |
| cg19826026 | ***ARHGDIB---Cluster1*** | ***ARHGDIB*** |  |
| cg14681767 | ARHGEF10 | *ARHGEF10* |  |
| cg08725962 | ***ARL10---Cluster1*** | ***ARL10*** |  |
| cg27092035 | ***ARL10---Cluster1*** | ***ARL10*** |  |
| cg22343001 | ARMCX2 | *ARMCX2* |  |
| cg08141989 | ASGR1---Cluster1 | *ASGR1* |  |
| cg20401945 | ASPHD1 | *ASPHD1* |  |
| cg12640109 | ASRGL1---Cluster1 | *ASRGL1* |  |
| cg10734665 | ***ATP10A---Cluster2*** | ***ATP10A*** |  |
| cg17793621 | ***ATP10A---Cluster2*** | ***ATP10A*** |  |
| cg19326876 | ***ATP10A---Cluster2*** | ***ATP10A*** |  |
| cg09580336 | ATP1A1 | *ATP1A1* |  |
| cg18085435 | ATP8B1---Cluster1 | *ATP8B1* |  |
| cg04240200 | ***ATRN---Cluster1*** | ***ATRN*** |  |
| cg17294081 | ***ATRN---Cluster1*** | ***ATRN*** |  |
| cg12019109 | ***AZGP1---Cluster1*** | ***AZGP1*** |  |
| cg19465374 | ***AZGP1---Cluster1*** | ***AZGP1*** |  |
| cg20557104 | B3GNT8 | *B3GNT8* |  |
| cg16004226 | ***B4GALNT4---Cluster1*** | ***B4GALNT4*** |  |
| cg24194132 | ***B4GALNT4---Cluster1*** | ***B4GALNT4*** |  |
| cg25023994 | BANK1 | *BANK1* |  |
| cg09937039 | ***BATF---Cluster1*** | ***BATF*** |  |
| cg15645309 | ***BATF---Cluster1*** | ***BATF*** |  |
| cg10027114 | ***BBS10---Cluster1*** | ***BBS10*** |  |
| cg26248486 | ***BBS10---Cluster1*** | ***BBS10*** |  |
| cg05847778 | ***BBS5---Cluster1*** | ***BBS5*** |  |
| cg10006582 | ***BBS5---Cluster1*** | ***BBS5*** |  |
| cg10764357 | BCAT1 | *BCAT1* |  |
| cg12549513 | ***BEX2---Cluster1*** | ***BEX2*** |  |
| cg24765005 | ***BEX2---Cluster1*** | ***BEX2*** |  |
| cg00968475 | ***BHLHB9---Cluster1*** | ***BHLHB9*** |  |
| cg15309236 | ***BHLHB9---Cluster1*** | ***BHLHB9*** |  |
| cg24526899 | BMP4---Cluster2 | *BMP4* |  |
| cg26516759 | BMP7 | *BMP7* |  |
| cg22473973 | BNIP3 | *BNIP3* |  |
| cg06605933 | ***BOLA1---Cluster1*** | ***BOLA1*** |  |
| cg09980622 | ***BOLA1---Cluster1*** | ***BOLA1*** |  |
| cg01254505 | ***BST2---Cluster1*** | ***BST2*** |  |
| cg16363586 | ***BST2---Cluster1*** | ***BST2*** |  |
| cg12251804 | C10orf125---Cluster1 | ***C10orf125*** |  |
| cg14607011 | C10orf125---Cluster2 | ***C10orf125*** |  |
| cg03411288 | ***C10orf47---Cluster1*** | ***C10orf47*** |  |
| cg13904771 | ***C10orf47---Cluster1*** | ***C10orf47*** |  |
| cg07621046 | ***C10orf82---Cluster1*** | ***C10orf82*** |  |
| cg17349199 | ***C10orf82---Cluster1*** | ***C10orf82*** |  |
| cg01558777 | C10orf99 | *C10orf99* |  |
| cg04237003 | ***C11orf9---Cluster1*** | ***C11orf9*** |  |
| cg22627427 | ***C11orf9---Cluster1*** | ***C11orf9*** |  |
| cg04513422 | C13orf29---Cluster1 | *C13orf29* |  |
| cg08085267 | C17orf57---Cluster1 | *C17orf57* |  |
| cg03016571 | C17orf73 | *C17orf73* |  |
| cg01543654 | ***C17orf93---Cluster1*** | ***C17orf93*** |  |
| cg12374721 | ***C17orf93---Cluster1*** | ***C17orf93*** |  |
| cg18542098 | ***C19orf46---Cluster1*** | ***C19orf46*** |  |
| cg21550442 | ***C19orf46---Cluster1*** | ***C19orf46*** |  |
| cg01471713 | C1orf115 | *C1orf115* |  |
| cg09563216 | C1orf51---Cluster1 | ***C1orf51*** |  |
| cg21715963 | C1orf51---Cluster2 | ***C1orf51*** |  |
| cg00328227 | ***C1orf59---Cluster1*** | ***C1orf59*** |  |
| cg08460435 | ***C1orf59---Cluster1*** | ***C1orf59*** |  |
| cg04763192 | ***C1orf88---Cluster1*** | ***C1orf88*** |  |
| cg21926138 | ***C1orf88---Cluster1*** | ***C1orf88*** |  |
| cg14301635 | C2---Cluster1 | *C2* |  |
| cg02537838 | C20orf151---Cluster1 | *C20orf151* |  |
| cg05486551 | C20orf196 | *C20orf196* |  |
| cg22751696 | C3orf14---Cluster2 | *C3orf14* |  |
| cg01663469 | C3orf15 | *C3orf15* |  |
| cg00393585 | C4orf39 | *C4orf39* |  |
| cg20203395 | C5orf35 | *C5orf35* |  |
| cg14178895 | C6orf105 | *C6orf105* |  |
| cg25211525 | C6orf145 | *C6orf145* |  |
| cg16150435 | C6orf15 | *C6orf15* |  |
| cg00463577 | ***C6orf150---Cluster1*** | ***C6orf150*** |  |
| cg09527362 | ***C6orf150---Cluster1*** | ***C6orf150*** |  |
| cg04329454 | ***C6orf227---Cluster1*** | ***C6orf227*** |  |
| cg08301503 | ***C6orf227---Cluster1*** | ***C6orf227*** |  |
| cg22284975 | C7orf10---Cluster2 | *C7orf10* |  |
| cg01758870 | ***C7orf46---Cluster1*** | ***C7orf46*** |  |
| cg08707078 | ***C7orf46---Cluster1*** | ***C7orf46*** |  |
| cg07717632 | C9orf167---Cluster1 | ***C9orf167*** |  |
| cg13085976 | C9orf167---Cluster2 | ***C9orf167*** |  |
| cg13666340 | C9orf68 | *C9orf68* |  |
| cg15207619 | ***CAB39L---Cluster1*** | ***CAB39L*** |  |
| cg17777592 | ***CAB39L---Cluster1*** | ***CAB39L*** |  |
| cg01942127 | CACNA1D---Cluster1 | *CACNA1D* |  |
| cg01805540 | ***CACNB2---Cluster1*** | ***CACNB2*** |  |
| cg18408326 | ***CACNB2---Cluster1*** | ***CACNB2*** |  |
| cg09892203 | ***CACNG4---Cluster1*** | ***CACNG4*** |  |
| cg26743024 | ***CACNG4---Cluster1*** | ***CACNG4*** |  |
| cg21458907 | ***CADPS---Cluster1*** | ***CADPS*** |  |
| cg24505341 | ***CADPS---Cluster1*** | ***CADPS*** |  |
| cg02831294 | CALML3---Cluster1 | *CALML3* |  |
| cg16992787 | CAPS | *CAPS* |  |
| cg26937500 | CARD11---Cluster2 | *CARD11* |  |
| cg14893129 | CARD14 | *CARD14* |  |
| cg02516189 | CARD9---Cluster1 | *CARD9* |  |
| cg00695416 | ***CBR1---Cluster1*** | ***CBR1*** |  |
| cg01962969 | ***CBR1---Cluster1*** | ***CBR1*** |  |
| cg09622447 | ***CBS---Cluster1*** | ***CBS*** |  |
| cg22633722 | ***CBS---Cluster1*** | ***CBS*** |  |
| cg04126816 | ***CCDC106---Cluster1*** | ***CCDC106*** |  |
| cg12582959 | ***CCDC106---Cluster1*** | ***CCDC106*** |  |
| cg00056767 | CCDC122 | *CCDC122* |  |
| cg11263296 | CCDC64---Cluster1 | *CCDC64* |  |
| cg00022866 | CCDC88B | *CCDC88B* |  |
| cg14137939 | ***CCDC89---Cluster1*** | ***CCDC89*** |  |
| cg24939733 | ***CCDC89---Cluster1*** | ***CCDC89*** |  |
| cg02647265 | CCDC92---Cluster1 | *CCDC92* |  |
| cg23743114 | CCL15 | *CCL15* |  |
| cg00350296 | CD248 | *CD248* |  |
| cg03583857 | ***CD34---Cluster1*** | ***CD34*** |  |
| cg19591881 | ***CD34---Cluster1*** | ***CD34*** |  |
| cg07728874 | ***CD3D---Cluster1*** | ***CD3D*** |  |
| cg24841244 | ***CD3D---Cluster1*** | ***CD3D*** |  |
| cg21601405 | CD40 | *CD40* |  |
| cg08519905 | CD9---Cluster1 | *CD9* |  |
| cg24860534 | CDC42BPA | *CDC42BPA* |  |
| cg03620376 | ***CDC42EP5---Cluster1*** | ***CDC42EP5*** |  |
| cg09227563 | ***CDC42EP5---Cluster1*** | ***CDC42EP5*** |  |
| cg12038710 | CDH17---Cluster1 | *CDH17* |  |
| cg06948937 | ***CDK18---Cluster1*** | ***CDK18*** |  |
| cg07713493 | ***CDK18---Cluster1*** | ***CDK18*** |  |
| cg07644368 | ***CDO1---Cluster1*** | ***CDO1*** |  |
| cg12880658 | ***CDO1---Cluster1*** | ***CDO1*** |  |
| cg21505334 | CEACAM5 | *CEACAM5* |  |
| cg21126943 | ***CEACAM6---Cluster1*** | ***CEACAM6*** |  |
| cg26813458 | ***CEACAM6---Cluster1*** | ***CEACAM6*** |  |
| cg03693099 | ***CEL---Cluster1*** | ***CEL*** |  |
| cg17961200 | ***CEL---Cluster1*** | ***CEL*** |  |
| cg09736162 | CELSR3---Cluster2 | *CELSR3* |  |
| cg13969584 | CENPBD1 | *CENPBD1* |  |
| cg05698090 | CERK | *CERK* |  |
| cg09407859 | ***CES3---Cluster1*** | ***CES3*** |  |
| cg26538442 | ***CES3---Cluster1*** | ***CES3*** |  |
| cg25509184 | CFTR | *CFTR* |  |
| cg22740783 | CGREF1 | *CGREF1* |  |
| cg14318370 | ***CHCHD5---Cluster1*** | ***CHCHD5*** |  |
| cg24651706 | ***CHCHD5---Cluster1*** | ***CHCHD5*** |  |
| cg00338702 | ***CHFR---Cluster1*** | ***CHFR*** |  |
| cg04044684 | ***CHFR---Cluster1*** | ***CHFR*** |  |
| cg19027571 | ***CHFR---Cluster1*** | ***CHFR*** |  |
| cg27040423 | ***CHFR---Cluster1*** | ***CHFR*** |  |
| cg21472642 | CHN2---Cluster2 | *CHN2* |  |
| cg15309006 | CHP2---Cluster1 | *CHP2* |  |
| cg03853987 | ***CHST10---Cluster1*** | ***CHST10*** |  |
| cg14372394 | ***CHST10---Cluster1*** | ***CHST10*** |  |
| cg00840403 | ***CHST4---Cluster1*** | ***CHST4*** |  |
| cg05535113 | ***CHST4---Cluster1*** | ***CHST4*** |  |
| cg10313673 | ***CILP2---Cluster1*** | ***CILP2*** |  |
| cg10669058 | ***CILP2---Cluster1*** | ***CILP2*** |  |
| cg02512860 | ***CLDN15---Cluster1*** | ***CLDN15*** |  |
| cg07651914 | ***CLDN15---Cluster1*** | ***CLDN15*** |  |
| cg10784090 | ***CLDN18---Cluster1*** | ***CLDN18*** |  |
| cg17298704 | ***CLDN18---Cluster1*** | ***CLDN18*** |  |
| cg10722799 | CLIC6 | *CLIC6* |  |
| cg01777397 | ***CLIP4---Cluster1*** | ***CLIP4*** |  |
| cg21972382 | ***CLIP4---Cluster1*** | ***CLIP4*** |  |
| cg23817637 | CLRN3 | *CLRN3* |  |
| cg08853659 | ***CLSTN2---Cluster1*** | ***CLSTN2*** |  |
| cg14498423 | ***CLSTN2---Cluster1*** | ***CLSTN2*** |  |
| cg11882252 | CMBL---Cluster1 | *CMBL* |  |
| cg16335762 | ***CMTM3---Cluster1*** | ***CMTM3*** |  |
| cg23297477 | ***CMTM3---Cluster1*** | ***CMTM3*** |  |
| cg07080358 | CNRIP1---Cluster1 | *CNRIP1* |  |
| cg22325715 | CNTD1---Cluster2 | *CNTD1* |  |
| cg09944147 | COL27A1 | *COL27A1* |  |
| cg21750887 | CPA6 | *CPA6* |  |
| cg02886284 | ***CPE---Cluster1*** | ***CPE*** |  |
| cg26812418 | ***CPE---Cluster1*** | ***CPE*** |  |
| cg07690018 | CPNE7 | *CPNE7* |  |
| cg20857455 | ***CPNE8---Cluster1*** | ***CPNE8*** |  |
| cg23495733 | ***CPNE8---Cluster1*** | ***CPNE8*** |  |
| cg15350036 | CROT---Cluster2 | *CROT* |  |
| cg26928972 | CSTA | *CSTA* |  |
| cg17800442 | CTF1 | *CTF1* |  |
| cg17974185 | CTNNBL1 | *CTNNBL1* |  |
| cg21478437 | CTSE | *CTSE* |  |
| cg06817264 | CTSF | *CTSF* |  |
| cg21880328 | ***CTTNBP2---Cluster1*** | ***CTTNBP2*** |  |
| cg27603796 | ***CTTNBP2---Cluster1*** | ***CTTNBP2*** |  |
| cg04005707 | ***CWH43---Cluster1*** | ***CWH43*** |  |
| cg25484904 | ***CWH43---Cluster1*** | ***CWH43*** |  |
| cg19790294 | CYBA | *CYBA* |  |
| cg02604290 | ***CYP24A1---Cluster1*** | ***CYP24A1*** |  |
| cg18956481 | ***CYP24A1---Cluster1*** | ***CYP24A1*** |  |
| cg03460682 | CYP27A1 | *CYP27A1* |  |
| cg15914863 | ***CYP2W1---Cluster1*** | ***CYP2W1*** |  |
| cg22025233 | ***CYP2W1---Cluster1*** | ***CYP2W1*** |  |
| cg13656062 | CYP4F2---Cluster2 | *CYP4F2* |  |
| cg15746719 | ***DAPK1---Cluster3*** | ***DAPK1*** |  |
| cg19734228 | ***DAPK1---Cluster3*** | ***DAPK1*** |  |
| cg24754277 | ***DAPK1---Cluster3*** | ***DAPK1*** |  |
| cg21614638 | DAPP1 | *DAPP1* |  |
| cg07435592 | ***DCBLD2---Cluster1*** | ***DCBLD2*** |  |
| cg09615786 | ***DCBLD2---Cluster1*** | ***DCBLD2*** |  |
| cg07903860 | DCLRE1C | *DCLRE1C* |  |
| cg18055007 | DDAH2---Cluster2 | *DDAH2* |  |
| cg08124399 | DDX43 | *DDX43* |  |
| cg25568243 | ***DEM1---Cluster1*** | ***DEM1*** |  |
| cg26776069 | ***DEM1---Cluster1*** | ***DEM1*** |  |
| cg13634319 | DGKA---Cluster1 | *DGKA* |  |
| cg05600717 | DHRS12 | *DHRS12* |  |
| cg04623955 | DIO3 | *DIO3* |  |
| cg06537230 | ***DLX5---Cluster4*** | ***DLX5*** |  |
| cg06911084 | ***DLX5---Cluster4*** | ***DLX5*** |  |
| cg09150117 | ***DLX5---Cluster4*** | ***DLX5*** |  |
| cg16924616 | ***DLX5---Cluster4*** | ***DLX5*** |  |
| cg20120491 | DMC1---Cluster2 | *DMC1* |  |
| cg01017147 | DNM3 | *DNM3* |  |
| cg21629895 | DNMT3A---Cluster2 | *DNMT3A* |  |
| cg21233722 | DOCK2 | *DOCK2* |  |
| cg02034222 | DQX1 | *DQX1* |  |
| cg17307280 | DRD1 | *DRD1* |  |
| cg23756219 | DRP2---Cluster2 | *DRP2* |  |
| cg11832722 | ***DSC3---Cluster1*** | ***DSC3*** |  |
| cg15439862 | ***DSC3---Cluster1*** | ***DSC3*** |  |
| cg13460409 | DSCR6---Cluster2 | *DSCR6* |  |
| cg04836428 | ***DTNA---Cluster1*** | ***DTNA*** |  |
| cg22646528 | ***DTNA---Cluster1*** | ***DTNA*** |  |
| cg00877887 | ***DUOXA2---Cluster1*** | ***DUOXA2*** |  |
| cg04001842 | ***DUOXA2---Cluster1*** | ***DUOXA2*** |  |
| cg11104347 | DUSP23 | *DUSP23* |  |
| cg10294836 | DYRK1B | *DYRK1B* |  |
| cg23960723 | ***ECHDC3---Cluster1*** | ***ECHDC3*** |  |
| cg24428042 | ***ECHDC3---Cluster1*** | ***ECHDC3*** |  |
| cg23582408 | EEF1A2 | *EEF1A2* |  |
| cg14759043 | EFCAB4B | *EFCAB4B* |  |
| cg20786074 | ***EFEMP1---Cluster1*** | ***EFEMP1*** |  |
| cg25711779 | ***EFEMP1---Cluster1*** | ***EFEMP1*** |  |
| cg03919781 | ***EFHA2---Cluster1*** | ***EFHA2*** |  |
| cg26831415 | ***EFHA2---Cluster1*** | ***EFHA2*** |  |
| cg13379236 | ***EGF---Cluster1*** | ***EGF*** |  |
| cg24818418 | ***EGF---Cluster1*** | ***EGF*** |  |
| cg07623294 | ELAVL2 | *ELAVL2* |  |
| cg00024396 | ***ELOVL5---Cluster1*** | ***ELOVL5*** |  |
| cg21195414 | ***ELOVL5---Cluster1*** | ***ELOVL5*** |  |
| cg20988616 | ENPP3 | *ENPP3* |  |
| cg04499325 | EPDR1---Cluster1 | *EPDR1* |  |
| cg18150721 | EPHB1 | *EPHB1* |  |
| cg15156367 | EPHX4---Cluster2 | *EPHX4* |  |
| cg00893636 | EPM2AIP1---Cluster1 | ***EPM2AIP1*** |  |
| cg10990993 | ***EPM2AIP1---Cluster2*** | ***EPM2AIP1*** |  |
| cg24607398 | ***EPM2AIP1---Cluster2*** | ***EPM2AIP1*** |  |
| cg04941721 | EREG---Cluster1 | ***EREG*** |  |
| cg19308222 | EREG---Cluster2 | ***EREG*** |  |
| cg09549827 | ***ESPN---Cluster1*** | ***ESPN*** |  |
| cg13066963 | ***ESPN---Cluster1*** | ***ESPN*** |  |
| cg12616277 | ESYT3 | *ESYT3* |  |
| cg04289385 | ETV7---Cluster1 | *ETV7* |  |
| cg18621299 | EVL---Cluster2 | *EVL* |  |
| cg13043862 | ***EYA1---Cluster1*** | ***EYA1*** |  |
| cg16793061 | ***EYA1---Cluster1*** | ***EYA1*** |  |
| cg09306675 | ***EYA2---Cluster1*** | ***EYA2*** |  |
| cg23214267 | ***EYA2---Cluster1*** | ***EYA2*** |  |
| cg00371195 | ***F2---Cluster1*** | ***F2*** |  |
| cg20199333 | ***F2---Cluster1*** | ***F2*** |  |
| cg19910382 | FABP1 | *FABP1* |  |
| cg14407437 | FABP3 | *FABP3* |  |
| cg06781209 | ***FADS2---Cluster1*** | ***FADS2*** |  |
| cg07005513 | ***FADS2---Cluster1*** | ***FADS2*** |  |
| cg24238205 | FAM111A | *FAM111A* |  |
| cg20484352 | FAM114A1---Cluster2 | *FAM114A1* |  |
| cg14147105 | FAM125A---Cluster2 | *FAM125A* |  |
| cg04922020 | ***FAM127A---Cluster1*** | ***FAM127A*** |  |
| cg27198071 | ***FAM127A---Cluster1*** | ***FAM127A*** |  |
| cg17162024 | ***FAM150A---Cluster1*** | ***FAM150A*** |  |
| cg26021627 | ***FAM150A---Cluster1*** | ***FAM150A*** |  |
| cg02194211 | ***FAM3D---Cluster1*** | ***FAM3D*** |  |
| cg21770617 | ***FAM3D---Cluster1*** | ***FAM3D*** |  |
| cg01570885 | FAM50B | *FAM50B* |  |
| cg27227156 | FAM55A | *FAM55A* |  |
| cg23067535 | FAM83A | *FAM83A* |  |
| cg00221494 | FARP1---Cluster1 | *FARP1* |  |
| cg01420388 | FBXO44---Cluster1 | *FBXO44* |  |
| cg24030627 | FCGBP---Cluster2 | *FCGBP* |  |
| cg14654385 | ***FERMT3---Cluster2*** | ***FERMT3*** |  |
| cg24088438 | ***FERMT3---Cluster2*** | ***FERMT3*** |  |
| cg18661868 | FES | *FES* |  |
| cg10261191 | FEZ1---Cluster2 | *FEZ1* |  |
| cg17214107 | FGF2---Cluster1 | *FGF2* |  |
| cg17410236 | ***FLRT2---Cluster1*** | ***FLRT2*** |  |
| cg26651233 | ***FLRT2---Cluster1*** | ***FLRT2*** |  |
| cg00489401 | FLT4---Cluster1 | *FLT4* |  |
| cg04587829 | ***FN3K---Cluster1*** | ***FN3K*** |  |
| cg21697134 | ***FN3K---Cluster1*** | ***FN3K*** |  |
| cg03699566 | FOLR1 | *FOLR1* |  |
| cg05113558 | FOXH1---Cluster2 | *FOXH1* |  |
| cg05475277 | FOXRED2 | *FOXRED2* |  |
| cg24964368 | FPR2 | *FPR2* |  |
| cg02784848 | ***FUZ---Cluster1*** | ***FUZ*** |  |
| cg10971790 | ***FUZ---Cluster1*** | ***FUZ*** |  |
| cg23054883 | FZD10 | *FZD10* |  |
| cg04464446 | GAL---Cluster1 | ***GAL*** |  |
| cg08785534 | GAL---Cluster2 | ***GAL*** |  |
| cg24058132 | GALC | *GALC* |  |
| cg13991233 | GAS7 | *GAS7* |  |
| cg01169778 | ***GBGT1---Cluster1*** | ***GBGT1*** |  |
| cg18089000 | ***GBGT1---Cluster1*** | ***GBGT1*** |  |
| cg08586737 | GCC1---Cluster1 | *GCC1* |  |
| cg07773116 | GDF10 | *GDF10* |  |
| cg25685838 | GDPD2 | *GDPD2* |  |
| cg09350274 | GFRA3 | *GFRA3* |  |
| cg07148914 | ***GGT7---Cluster1*** | ***GGT7*** |  |
| cg13514050 | ***GGT7---Cluster1*** | ***GGT7*** |  |
| cg01333788 | GJB5 | *GJB5* |  |
| cg20848130 | ***GJC2---Cluster1*** | ***GJC2*** |  |
| cg22844623 | ***GJC2---Cluster1*** | ***GJC2*** |  |
| cg11747771 | ***GLDC---Cluster1*** | ***GLDC*** |  |
| cg17009433 | ***GLDC---Cluster1*** | ***GLDC*** |  |
| cg02314308 | ***GLT25D2---Cluster1*** | ***GLT25D2*** |  |
| cg14313310 | ***GLT25D2---Cluster1*** | ***GLT25D2*** |  |
| cg17483510 | GNB4---Cluster2 | *GNB4* |  |
| cg02780849 | GNG4 | *GNG4* |  |
| cg21264055 | GNL1 | *GNL1* |  |
| cg12554476 | GP2---Cluster1 | *GP2* |  |
| cg06665322 | GPA33 | *GPA33* |  |
| cg21229055 | GPM6B---Cluster2 | *GPM6B* |  |
| cg13702536 | ***GPR81---Cluster1*** | ***GPR81*** |  |
| cg22534509 | ***GPR81---Cluster1*** | ***GPR81*** |  |
| cg11617144 | ***GPT---Cluster1*** | ***GPT*** |  |
| cg15983520 | ***GPT---Cluster1*** | ***GPT*** |  |
| cg20764656 | GPX2---Cluster2 | *GPX2* |  |
| cg16557944 | ***GPX7---Cluster1*** | ***GPX7*** |  |
| cg22129364 | ***GPX7---Cluster1*** | ***GPX7*** |  |
| cg10057218 | ***GSDMB---Cluster1*** | ***GSDMB*** |  |
| cg12360886 | ***GSDMB---Cluster1*** | ***GSDMB*** |  |
| cg22601215 | GSPT2---Cluster2 | *GSPT2* |  |
| cg23472215 | ***GSTM3---Cluster1*** | ***GSTM3*** |  |
| cg25027501 | ***GSTM3---Cluster1*** | ***GSTM3*** |  |
| cg05244766 | GSTP1---Cluster2 | *GSTP1* |  |
| cg03506684 | ***GYG2---Cluster1*** | ***GYG2*** |  |
| cg05113908 | ***GYG2---Cluster1*** | ***GYG2*** |  |
| cg17105014 | GYPC | *GYPC* |  |
| cg11716026 | ***H19---Cluster3*** | ***H19*** |  |
| cg23977670 | ***H19---Cluster3*** | ***H19*** |  |
| cg07141002 | H1F0 | *H1F0* |  |
| cg14617642 | ***H2AFY2---Cluster1*** | ***H2AFY2*** |  |
| cg17163751 | ***H2AFY2---Cluster1*** | ***H2AFY2*** |  |
| cg01561916 | ***HAAO---Cluster1*** | ***HAAO*** |  |
| cg20289949 | ***HAAO---Cluster1*** | ***HAAO*** |  |
| cg18833140 | ***HABP2---Cluster1*** | ***HABP2*** |  |
| cg26656452 | ***HABP2---Cluster1*** | ***HABP2*** |  |
| cg01580681 | ***HAND2---Cluster1*** | ***HAND2*** |  |
| cg02774439 | ***HAND2---Cluster1*** | ***HAND2*** |  |
| cg07971188 | ***HCP5---Cluster1*** | ***HCP5*** |  |
| cg13784557 | ***HCP5---Cluster1*** | ***HCP5*** |  |
| cg05446471 | HDAC11---Cluster2 | *HDAC11* |  |
| cg06466797 | ***HERPUD2---Cluster1*** | ***HERPUD2*** |  |
| cg08703595 | ***HERPUD2---Cluster1*** | ***HERPUD2*** |  |
| cg24127874 | HES6---Cluster2 | *HES6* |  |
| cg14776962 | ***HIST1H2BH---Cluster1*** | ***HIST1H2BH*** |  |
| cg21663122 | ***HIST1H2BH---Cluster1*** | ***HIST1H2BH*** |  |
| cg11639651 | HKDC1 | *HKDC1* |  |
| cg12024906 | ***HKR1---Cluster1*** | ***HKR1*** |  |
| cg14166009 | ***HKR1---Cluster1*** | ***HKR1*** |  |
| cg04836786 | ***HLTF---Cluster2*** | ***HLTF*** |  |
| cg26151310 | ***HLTF---Cluster2*** | ***HLTF*** |  |
| cg19717150 | ***HNF4A---Cluster1*** | ***HNF4A*** |  |
| cg23834593 | ***HNF4A---Cluster1*** | ***HNF4A*** |  |
| cg15760840 | ***HOXA11AS---Cluster1*** | ***HOXA11AS*** |  |
| cg17950095 | ***HOXA11AS---Cluster1*** | ***HOXA11AS*** |  |
| cg23432345 | ***HOXA7---Cluster1*** | ***HOXA7*** |  |
| cg26511321 | ***HOXA7---Cluster1*** | ***HOXA7*** |  |
| cg01354473 | ***HOXA9---Cluster1*** | ***HOXA9*** |  |
| cg01381846 | ***HOXA9---Cluster1*** | ***HOXA9*** |  |
| cg27009703 | ***HOXA9---Cluster1*** | ***HOXA9*** |  |
| cg09313705 | ***HOXB2---Cluster1*** | ***HOXB2*** |  |
| cg25882366 | ***HOXB2---Cluster1*** | ***HOXB2*** |  |
| cg12910797 | HOXB3 | *HOXB3* |  |
| cg02422694 | ***HOXB4---Cluster1*** | ***HOXB4*** |  |
| cg04609859 | ***HOXB4---Cluster1*** | ***HOXB4*** |  |
| cg08089301 | ***HOXB4---Cluster1*** | ***HOXB4*** |  |
| cg21460081 | ***HOXB4---Cluster1*** | ***HOXB4*** |  |
| cg15539420 | ***HOXB8---Cluster1*** | ***HOXB8*** |  |
| cg25928579 | ***HOXB8---Cluster1*** | ***HOXB8*** |  |
| cg10164640 | ***HPDL---Cluster1*** | ***HPDL*** |  |
| cg26781150 | ***HPDL---Cluster1*** | ***HPDL*** |  |
| cg26045434 | HR---Cluster2 | *HR* |  |
| cg09231514 | HSD11B1L---Cluster1 | *HSD11B1L* |  |
| cg03665457 | ***HSD17B7P2---Cluster1*** | ***HSD17B7P2*** |  |
| cg18963800 | ***HSD17B7P2---Cluster1*** | ***HSD17B7P2*** |  |
| cg16319578 | ***HSPA2---Cluster3*** | ***HSPA2*** |  |
| cg24642523 | ***HSPA2---Cluster3*** | ***HSPA2*** |  |
| cg24673765 | HSPB6 | *HSPB6* |  |
| cg05346140 | HSPB9 | *HSPB9* |  |
| cg18788940 | HTATIP2---Cluster2 | *HTATIP2* |  |
| cg12535715 | ***HTRA4---Cluster1*** | ***HTRA4*** |  |
| cg27258399 | ***HTRA4---Cluster1*** | ***HTRA4*** |  |
| cg25048564 | HUNK | *HUNK* |  |
| cg09794131 | ***HYDIN---Cluster1*** | ***HYDIN*** |  |
| cg20977864 | ***HYDIN---Cluster1*** | ***HYDIN*** |  |
| cg00468146 | ***ID4---Cluster1*** | ***ID4*** |  |
| cg17252960 | ***ID4---Cluster1*** | ***ID4*** |  |
| cg01493517 | IFFO1 | *IFFO1* |  |
| cg08843492 | IFITM2 | *IFITM2* |  |
| cg01305421 | IGF1 | *IGF1* |  |
| cg01747665 | IGFALS---Cluster1 | *IGFALS* |  |
| cg25854162 | ***IGFBP2---Cluster1*** | ***IGFBP2*** |  |
| cg26187237 | ***IGFBP2---Cluster1*** | ***IGFBP2*** |  |
| cg15077070 | ***IL1RL2---Cluster1*** | ***IL1RL2*** |  |
| cg22797169 | ***IL1RL2---Cluster1*** | ***IL1RL2*** |  |
| cg06392589 | IL20RB | *IL20RB* |  |
| cg00294382 | IL23A | *IL23A* |  |
| cg07509155 | ***IL28RA---Cluster1*** | ***IL28RA*** |  |
| cg08700651 | ***IL28RA---Cluster1*** | ***IL28RA*** |  |
| cg01361446 | ***IL2RG---Cluster1*** | ***IL2RG*** |  |
| cg15201909 | ***IL2RG---Cluster1*** | ***IL2RG*** |  |
| cg23642747 | INA | *INA* |  |
| cg22630748 | INHBE---Cluster2 | *INHBE* |  |
| cg07914866 | ***IRAK3---Cluster1*** | ***IRAK3*** |  |
| cg20395892 | ***IRAK3---Cluster1*** | ***IRAK3*** |  |
| cg26504021 | IRX2 | *IRX2* |  |
| cg21410991 | ISL1---Cluster1 | *ISL1* |  |
| cg06469542 | ***ISM2---Cluster1*** | ***ISM2*** |  |
| cg07965823 | ***ISM2---Cluster1*** | ***ISM2*** |  |
| cg13882267 | ITGA9 | *ITGA9* |  |
| cg15337006 | ITGAM | *ITGAM* |  |
| cg24625128 | JAM3 | *JAM3* |  |
| cg24924779 | KCNG1---Cluster2 | *KCNG1* |  |
| cg20406482 | ***KCNH4---Cluster1*** | ***KCNH4*** |  |
| cg23559331 | ***KCNH4---Cluster1*** | ***KCNH4*** |  |
| cg10778619 | ***KCNMB4---Cluster1*** | ***KCNMB4*** |  |
| cg23096553 | ***KCNMB4---Cluster1*** | ***KCNMB4*** |  |
| cg20673481 | ***KCNS3---Cluster1*** | ***KCNS3*** |  |
| cg20940661 | ***KCNS3---Cluster1*** | ***KCNS3*** |  |
| cg14323109 | KDR | *KDR* |  |
| cg19531130 | KIAA1377 | *KIAA1377* |  |
| cg02838492 | KIF12 | *KIF12* |  |
| cg17001430 | KIF25 | *KIF25* |  |
| cg18292711 | KIF5B---Cluster2 | *KIF5B* |  |
| cg02983451 | KLF11 | *KLF11* |  |
| cg03770147 | ***KLF7---Cluster1*** | ***KLF7*** |  |
| cg08478189 | ***KLF7---Cluster1*** | ***KLF7*** |  |
| cg06494770 | KLHL13 | *KLHL13* |  |
| cg19884658 | KLHL21---Cluster2 | *KLHL21* |  |
| cg13847070 | KLHL3 | *KLHL3* |  |
| cg21655480 | KLHL34---Cluster2 | *KLHL34* |  |
| cg16547529 | KLHL35---Cluster2 | *KLHL35* |  |
| cg00091693 | ***KRT20---Cluster1*** | ***KRT20*** |  |
| cg25124433 | ***KRT20---Cluster1*** | ***KRT20*** |  |
| cg06378617 | ***KRT23---Cluster1*** | ***KRT23*** |  |
| cg22392708 | ***KRT23---Cluster1*** | ***KRT23*** |  |
| cg07007400 | KRT7 | *KRT7* |  |
| cg17255302 | KRT75---Cluster1 | *KRT75* |  |
| cg14850026 | L1TD1 | *L1TD1* |  |
| cg14155416 | ***L3MBTL4---Cluster1*** | ***L3MBTL4*** |  |
| cg17688525 | ***L3MBTL4---Cluster1*** | ***L3MBTL4*** |  |
| cg12424817 | LAPTM4B | *LAPTM4B* |  |
| cg12732155 | LAPTM5 | *LAPTM5* |  |
| cg00351011 | ***LASS4---Cluster1*** | ***LASS4*** |  |
| cg05346899 | ***LASS4---Cluster1*** | ***LASS4*** |  |
| cg23663476 | ***LAT---Cluster1*** | ***LAT*** |  |
| cg23797100 | ***LAT---Cluster1*** | ***LAT*** |  |
| cg03243946 | ***LDHB---Cluster1*** | ***LDHB*** |  |
| cg06437004 | ***LDHB---Cluster1*** | ***LDHB*** |  |
| cg14562990 | ***LDOC1---Cluster1*** | ***LDOC1*** |  |
| cg20104776 | ***LDOC1---Cluster1*** | ***LDOC1*** |  |
| cg12319004 | LEFTY1 | *LEFTY1* |  |
| cg18626709 | ***LEPREL1---Cluster1*** | ***LEPREL1*** |  |
| cg20270599 | ***LEPREL1---Cluster1*** | ***LEPREL1*** |  |
| cg11081833 | LGALS2 | *LGALS2* |  |
| cg16731016 | LGALS4 | *LGALS4* |  |
| cg02988947 | LIMD2---Cluster1 | *LIMD2* |  |
| cg05647859 | LIN7A | *LIN7A* |  |
| cg01733599 | LIPC---Cluster1 | *LIPC* |  |
| cg23131007 | LOC145783 | *LOC145783* |  |
| cg15052335 | LPIN2---Cluster1 | *LPIN2* |  |
| cg08918749 | ***LPL---Cluster1*** | ***LPL*** |  |
| cg22108175 | ***LPL---Cluster1*** | ***LPL*** |  |
| cg20654468 | LPXN | *LPXN* |  |
| cg13526007 | LRFN5 | *LRFN5* |  |
| cg24926276 | LRG1 | *LRG1* |  |
| cg09531892 | ***LRP12---Cluster1*** | ***LRP12*** |  |
| cg24098951 | ***LRP12---Cluster1*** | ***LRP12*** |  |
| cg10031651 | LRRC2---Cluster1 | *LRRC2* |  |
| cg04698187 | LRRN2---Cluster1 | *LRRN2* |  |
| cg08965235 | ***LTBP3---Cluster1*** | ***LTBP3*** |  |
| cg16632280 | ***LTBP3---Cluster1*** | ***LTBP3*** |  |
| cg07654934 | LXN---Cluster1 | *LXN* |  |
| cg04678793 | ***LY6E---Cluster1*** | ***LY6E*** |  |
| cg06597861 | ***LY6E---Cluster1*** | ***LY6E*** |  |
| cg02212836 | LY86---Cluster1 | *LY86* |  |
| cg23732024 | LY96 | *LY96* |  |
| cg16097772 | LYZ | *LYZ* |  |
| cg02497758 | MAFB | *MAFB* |  |
| cg04544498 | ***MAGEE1---Cluster1*** | ***MAGEE1*** |  |
| cg12431196 | ***MAGEE1---Cluster1*** | ***MAGEE1*** |  |
| cg01172484 | ***MAGEH1---Cluster1*** | ***MAGEH1*** |  |
| cg18869368 | ***MAGEH1---Cluster1*** | ***MAGEH1*** |  |
| cg21245652 | MAL---Cluster2 | *MAL* |  |
| cg11920519 | MAP1LC3A---Cluster1 | *MAP1LC3A* |  |
| cg04898797 | MAP4K2 | *MAP4K2* |  |
| cg03616357 | MAP9 | *MAP9* |  |
| cg17192247 | MAPRE3 | *MAPRE3* |  |
| cg16202564 | MATN2---Cluster2 | *MATN2* |  |
| cg16019620 | ***MEP1A---Cluster1*** | ***MEP1A*** |  |
| cg20980592 | ***MEP1A---Cluster1*** | ***MEP1A*** |  |
| cg11201532 | MFNG---Cluster1 | *MFNG* |  |
| cg17952262 | ***MFSD7---Cluster1*** | ***MFSD7*** |  |
| cg24693053 | ***MFSD7---Cluster1*** | ***MFSD7*** |  |
| cg02941816 | ***MGMT---Cluster3*** | ***MGMT*** |  |
| cg12434587 | ***MGMT---Cluster3*** | ***MGMT*** |  |
| cg12981137 | ***MGMT---Cluster3*** | ***MGMT*** |  |
| cg26201213 | ***MGMT---Cluster3*** | ***MGMT*** |  |
| cg11203041 | MGST1 | *MGST1* |  |
| cg25152942 | MIA | *MIA* |  |
| cg10644361 | ***MIPOL1---Cluster1*** | ***MIPOL1*** |  |
| cg18909638 | ***MIPOL1---Cluster1*** | ***MIPOL1*** |  |
| cg05743054 | ***MLF1---Cluster1*** | ***MLF1*** |  |
| cg20182358 | ***MLF1---Cluster1*** | ***MLF1*** |  |
| cg13846866 | MLH1---Cluster2 | *MLH1* |  |
| cg16426459 | MLPH | *MLPH* |  |
| cg16580737 | ***MME---Cluster1*** | ***MME*** |  |
| cg23273897 | ***MME---Cluster1*** | ***MME*** |  |
| cg23149053 | ***MMP17---Cluster1*** | ***MMP17*** |  |
| cg24493940 | ***MMP17---Cluster1*** | ***MMP17*** |  |
| cg04316754 | MMP24 | *MMP24* |  |
| cg12531542 | MOGAT2 | *MOGAT2* |  |
| cg22722802 | MPV17L | *MPV17L* |  |
| cg18031008 | MRPS21 | *MRPS21* |  |
| cg06269753 | ***MSC---Cluster1*** | ***MSC*** |  |
| cg23710218 | ***MSC---Cluster1*** | ***MSC*** |  |
| cg02883161 | ***MSI1---Cluster1*** | ***MSI1*** |  |
| cg20380069 | ***MSI1---Cluster1*** | ***MSI1*** |  |
| cg06100324 | MSLN | *MSLN* |  |
| cg26796283 | MSX2 | *MSX2* |  |
| cg15134649 | ***MT1E---Cluster1*** | ***MT1E*** |  |
| cg20083730 | ***MT1E---Cluster1*** | ***MT1E*** |  |
| cg16158681 | MT3---Cluster1 | *MT3* |  |
| cg17054360 | MTERF | *MTERF* |  |
| cg09406238 | MTMR8 | *MTMR8* |  |
| cg24512973 | MUC1---Cluster4 | *MUC1* |  |
| cg09081544 | MUC13 | *MUC13* |  |
| cg01584473 | ***MUC17---Cluster1*** | ***MUC17*** |  |
| cg07873488 | ***MUC17---Cluster1*** | ***MUC17*** |  |
| cg09604203 | ***MYBL2---Cluster1*** | ***MYBL2*** |  |
| cg23843505 | ***MYBL2---Cluster1*** | ***MYBL2*** |  |
| cg20603888 | MYEF2---Cluster1 | ***MYEF2*** |  |
| cg21229859 | MYEF2---Cluster2 | ***MYEF2*** |  |
| cg06011292 | MYH10---Cluster2 | *MYH10* |  |
| cg23771603 | MYO3A | *MYO3A* |  |
| cg09296212 | NAIF1---Cluster1 | *NAIF1* |  |
| cg14494313 | NAT2 | *NAT2* |  |
| cg13297960 | NCAM2 | *NCAM2* |  |
| cg19917856 | NCCRP1 | *NCCRP1* |  |
| cg16509569 | ***NCKAP1L---Cluster1*** | ***NCKAP1L*** |  |
| cg17605084 | ***NCKAP1L---Cluster1*** | ***NCKAP1L*** |  |
| cg09347151 | ***NCRNA00086---Cluster1*** | ***NCRNA00086*** |  |
| cg17231524 | ***NCRNA00086---Cluster1*** | ***NCRNA00086*** |  |
| cg12532169 | ***NDN---Cluster2*** | ***NDN*** |  |
| cg13828758 | ***NDN---Cluster2*** | ***NDN*** |  |
| cg18552939 | ***NDN---Cluster2*** | ***NDN*** |  |
| cg02994956 | NEFH---Cluster1 | *NEFH* |  |
| cg23290344 | NEFM | *NEFM* |  |
| cg12978308 | NEIL1 | *NEIL1* |  |
| cg19524009 | NEK3 | *NEK3* |  |
| cg02755525 | NETO2 | *NETO2* |  |
| cg13510327 | ***NEU1---Cluster1*** | ***NEU1*** |  |
| cg14976276 | ***NEU1---Cluster1*** | ***NEU1*** |  |
| cg22571530 | NFASC | *NFASC* |  |
| cg10226546 | NFATC2 | *NFATC2* |  |
| cg00772000 | NHLRC1 | *NHLRC1* |  |
| cg09088834 | ***NINL---Cluster1*** | ***NINL*** |  |
| cg17729667 | ***NINL---Cluster1*** | ***NINL*** |  |
| cg20073553 | NKX3-2---Cluster2 | *NKX3-2* | Cluster2 |
| cg18059223 | NLRP2 | *NLRP2* |  |
| cg00707317 | NMNAT2 | *NMNAT2* |  |
| cg18488855 | NOVA1 | *NOVA1* |  |
| cg14477619 | ***NPC1L1---Cluster1*** | ***NPC1L1*** |  |
| cg17754680 | ***NPC1L1---Cluster1*** | ***NPC1L1*** |  |
| cg10279685 | NPHP1---Cluster2 | *NPHP1* |  |
| cg00548268 | NPTX2 | *NPTX2* |  |
| cg07611334 | ***NRSN2---Cluster1*** | ***NRSN2*** |  |
| cg08713365 | ***NRSN2---Cluster1*** | ***NRSN2*** |  |
| cg11053574 | NT5C3L | *NT5C3L* |  |
| cg23555120 | NUAK1 | *NUAK1* |  |
| cg02976617 | NUDT12 | *NUDT12* |  |
| cg23799276 | NUP210---Cluster1 | *NUP210* |  |
| cg05590982 | NUPR1 | *NUPR1* |  |
| cg16933922 | OAT | *OAT* |  |
| cg11510839 | ***OCA2---Cluster1*** | ***OCA2*** |  |
| cg12902039 | ***OCA2---Cluster1*** | ***OCA2*** |  |
| cg06222851 | OGDHL | *OGDHL* |  |
| cg27286999 | OLFM1---Cluster2 | *OLFM1* |  |
| cg12582008 | OLFM4 | *OLFM4* |  |
| cg17253459 | OLFML3 | *OLFML3* |  |
| cg02564523 | ORAI2 | *ORAI2* |  |
| cg03138091 | ***OSMR---Cluster1*** | ***OSMR*** |  |
| cg26475085 | ***OSMR---Cluster1*** | ***OSMR*** |  |
| cg00158308 | ***OXGR1---Cluster1*** | ***OXGR1*** |  |
| cg23037133 | ***OXGR1---Cluster1*** | ***OXGR1*** |  |
| cg04875162 | PABPC5 | *PABPC5* |  |
| cg01663295 | ***PACS1---Cluster1*** | ***PACS1*** |  |
| cg25034557 | ***PACS1---Cluster1*** | ***PACS1*** |  |
| cg15083233 | PALM2-AKAP2 | *PALM2* |  |
| cg02883230 | PARP6---Cluster1 | *PARP6* |  |
| cg09351859 | PARP8 | *PARP8* |  |
| cg00633969 | PARP9---Cluster1 | *PARP9* |  |
| cg13910855 | PBX2---Cluster2 | *PBX2* |  |
| cg01836044 | ***PCDH20---Cluster1*** | ***PCDH20*** |  |
| cg15182360 | ***PCDH20---Cluster1*** | ***PCDH20*** |  |
| cg09786257 | ***PCSK1---Cluster1*** | ***PCSK1*** |  |
| cg23187653 | ***PCSK1---Cluster1*** | ***PCSK1*** |  |
| cg07374632 | ***PCSK1N---Cluster1*** | ***PCSK1N*** |  |
| cg07526675 | ***PCSK1N---Cluster1*** | ***PCSK1N*** |  |
| cg01431114 | ***PDE10A---Cluster1*** | ***PDE10A*** |  |
| cg24133115 | ***PDE10A---Cluster1*** | ***PDE10A*** |  |
| cg23367478 | ***PDE3A---Cluster1*** | ***PDE3A*** |  |
| cg24975564 | ***PDE3A---Cluster1*** | ***PDE3A*** |  |
| cg10321723 | ***PDZK1---Cluster1*** | ***PDZK1*** |  |
| cg13019092 | ***PDZK1---Cluster1*** | ***PDZK1*** |  |
| cg15187606 | PDZK1IP1 | *PDZK1IP1* |  |
| cg01656470 | ***PEG3---Cluster1*** | ***PEG3*** |  |
| cg02793099 | ***PEG3---Cluster1*** | ***PEG3*** |  |
| cg19771589 | ***PEG3---Cluster1*** | ***PEG3*** |  |
| cg15754084 | PEX5---Cluster2 | *PEX5* |  |
| cg15158783 | ***PF4---Cluster1*** | ***PF4*** |  |
| cg16072462 | ***PF4---Cluster1*** | ***PF4*** |  |
| cg20357628 | ***PHACTR3---Cluster1*** | ***PHACTR3*** |  |
| cg20674577 | ***PHACTR3---Cluster1*** | ***PHACTR3*** |  |
| cg07090813 | ***PHGDH---Cluster1*** | ***PHGDH*** |  |
| cg26791905 | ***PHGDH---Cluster1*** | ***PHGDH*** |  |
| cg13645078 | ***PHLDA3---Cluster1*** | ***PHLDA3*** |  |
| cg20771670 | ***PHLDA3---Cluster1*** | ***PHLDA3*** |  |
| cg13686115 | ***PHYHIPL---Cluster1*** | ***PHYHIPL*** |  |
| cg25946758 | ***PHYHIPL---Cluster1*** | ***PHYHIPL*** |  |
| cg04743872 | PID1---Cluster2 | *PID1* |  |
| cg02105856 | ***PIGR---Cluster1*** | ***PIGR*** |  |
| cg20953047 | ***PIGR---Cluster1*** | ***PIGR*** |  |
| cg06144905 | ***PIPOX---Cluster1*** | ***PIPOX*** |  |
| cg22968401 | ***PIPOX---Cluster1*** | ***PIPOX*** |  |
| cg05522383 | PITX2---Cluster1 | *PITX2* |  |
| cg04689061 | ***PKIA---Cluster1*** | ***PKIA*** |  |
| cg27140220 | ***PKIA---Cluster1*** | ***PKIA*** |  |
| cg02280309 | PKLR | *PKLR* |  |
| cg09009380 | PKP1---Cluster1 | ***PKP1*** |  |
| cg19570545 | PKP1---Cluster2 | ***PKP1*** |  |
| cg02044879 | ***PLA2G12B---Cluster1*** | ***PLA2G12B*** |  |
| cg21820890 | ***PLA2G12B---Cluster1*** | ***PLA2G12B*** |  |
| cg22220722 | PLA2G2A | *PLA2G2A* |  |
| cg24402880 | PLAC8 | *PLAC8* |  |
| cg12371177 | PLEKHG6---Cluster1 | *PLEKHG6* |  |
| cg06797533 | PLIN2 | *PLIN2* |  |
| cg23713520 | PLLP | *PLLP* |  |
| cg08990057 | ***PLS3---Cluster1*** | ***PLS3*** |  |
| cg16221059 | ***PLS3---Cluster1*** | ***PLS3*** |  |
| cg02154186 | PNMA2 | *PNMA2* |  |
| cg04659622 | PNPLA4 | *PNPLA4* |  |
| cg08097882 | ***POU4F1---Cluster1*** | ***POU4F1*** |  |
| cg15604467 | ***POU4F1---Cluster1*** | ***POU4F1*** |  |
| cg14884741 | ***PPP1R14C---Cluster1*** | ***PPP1R14C*** |  |
| cg20134151 | ***PPP1R14C---Cluster1*** | ***PPP1R14C*** |  |
| cg04968426 | ***PPP1R14D---Cluster1*** | ***PPP1R14D*** |  |
| cg23382741 | ***PPP1R14D---Cluster1*** | ***PPP1R14D*** |  |
| cg27377213 | PPP1R16B---Cluster2 | *PPP1R16B* |  |
| cg04951204 | ***PPP1R9A---Cluster1*** | ***PPP1R9A*** |  |
| cg09643313 | ***PPP1R9A---Cluster1*** | ***PPP1R9A*** |  |
| cg11164400 | ***PPP1R9A---Cluster1*** | ***PPP1R9A*** |  |
| cg16872560 | ***PPP1R9A---Cluster1*** | ***PPP1R9A*** |  |
| cg19221959 | ***PPP1R9A---Cluster1*** | ***PPP1R9A*** |  |
| cg14368286 | ***PRAF2---Cluster1*** | ***PRAF2*** |  |
| cg20371650 | ***PRAF2---Cluster1*** | ***PRAF2*** |  |
| cg10742801 | PRAP1 | *PRAP1* |  |
| cg08537652 | PRDM16 | *PRDM16* |  |
| cg06836772 | PRKAA2 | *PRKAA2* |  |
| cg06628693 | ***PRKACB---Cluster1*** | ***PRKACB*** |  |
| cg23698058 | ***PRKACB---Cluster1*** | ***PRKACB*** |  |
| cg01620308 | ***PRNP---Cluster1*** | ***PRNP*** |  |
| cg15993674 | ***PRNP---Cluster1*** | ***PRNP*** |  |
| cg22730830 | PRSS21 | *PRSS21* |  |
| cg20670302 | ***PSTPIP2---Cluster1*** | ***PSTPIP2*** |  |
| cg21949781 | ***PSTPIP2---Cluster1*** | ***PSTPIP2*** |  |
| cg24989962 | PTGDR | *PTGDR* |  |
| cg08747889 | ***PTK7---Cluster1*** | ***PTK7*** |  |
| cg21663580 | ***PTK7---Cluster1*** | ***PTK7*** |  |
| cg12647643 | ***PTPN13---Cluster1*** | ***PTPN13*** |  |
| cg23478284 | ***PTPN13---Cluster1*** | ***PTPN13*** |  |
| cg02222362 | ***PTPN20B---Cluster1*** | ***PTPN20B*** |  |
| cg16192575 | ***PTPN20B---Cluster1*** | ***PTPN20B*** |  |
| cg14385738 | PTPN22 | *PTPN22* |  |
| cg12989642 | PURB | *PURB* |  |
| cg12100791 | ***PYCARD---Cluster3*** | ***PYCARD*** |  |
| cg15468095 | ***PYCARD---Cluster3*** | ***PYCARD*** |  |
| cg19850348 | PYGO1 | *PYGO1* |  |
| cg09339301 | ***QKI---Cluster1*** | ***QKI*** |  |
| cg25157874 | ***QKI---Cluster1*** | ***QKI*** |  |
| cg17982102 | RAB31---Cluster2 | *RAB31* |  |
| cg04113075 | ***RAB32---Cluster1*** | ***RAB32*** |  |
| cg14889768 | ***RAB32---Cluster1*** | ***RAB32*** |  |
| cg20098887 | ***RAB32---Cluster1*** | ***RAB32*** |  |
| cg22030890 | ***RAB32---Cluster1*** | ***RAB32*** |  |
| cg23833452 | ***RAB32---Cluster1*** | ***RAB32*** |  |
| cg05668853 | ***RAB34---Cluster1*** | ***RAB34*** |  |
| cg21237418 | ***RAB34---Cluster1*** | ***RAB34*** |  |
| cg03000846 | ***RAC3---Cluster1*** | ***RAC3*** |  |
| cg17787710 | ***RAC3---Cluster1*** | ***RAC3*** |  |
| cg02499249 | ***RARB---Cluster1*** | ***RARB*** |  |
| cg10712623 | ***RARB---Cluster1*** | ***RARB*** |  |
| cg26124016 | ***RARB---Cluster1*** | ***RARB*** |  |
| cg27486427 | ***RARB---Cluster1*** | ***RARB*** |  |
| cg17279839 | RARRES2---Cluster2 | *RARRES2* |  |
| cg02927346 | ***RASL10B---Cluster1*** | ***RASL10B*** |  |
| cg20496643 | ***RASL10B---Cluster1*** | ***RASL10B*** |  |
| cg06821120 | ***RASSF1---Cluster3*** | ***RASSF1*** |  |
| cg06980053 | ***RASSF1---Cluster3*** | ***RASSF1*** |  |
| cg07346310 | RBM24 | *RBM24* |  |
| cg06543018 | ***RBP1---Cluster1*** | ***RBP1*** |  |
| cg11027570 | ***RBP1---Cluster1*** | ***RBP1*** |  |
| cg12497564 | ***RBP1---Cluster1*** | ***RBP1*** |  |
| cg13099330 | ***RBP1---Cluster1*** | ***RBP1*** |  |
| cg23363832 | ***RBP1---Cluster1*** | ***RBP1*** |  |
| cg24594997 | ***RBP1---Cluster1*** | ***RBP1*** |  |
| cg27457941 | ***RBP1---Cluster1*** | ***RBP1*** |  |
| cg17778120 | RBP2 | *RBP2* |  |
| cg12936747 | RBP4 | *RBP4* |  |
| cg01968178 | ***REEP1---Cluster1*** | ***REEP1*** |  |
| cg02870945 | ***REEP1---Cluster1*** | ***REEP1*** |  |
| cg02674804 | REEP6---Cluster1 | *REEP6* |  |
| cg00808492 | REG4 | *REG4* |  |
| cg10414058 | ***RELL2---Cluster1*** | ***RELL2*** |  |
| cg24385322 | ***RELL2---Cluster1*** | ***RELL2*** |  |
| cg19205533 | RERG---Cluster2 | *RERG* |  |
| cg07654896 | ***RGN---Cluster1*** | ***RGN*** |  |
| cg09625066 | ***RGN---Cluster1*** | ***RGN*** |  |
| cg04041960 | RGS10---Cluster1 | *RGS10* |  |
| cg02026235 | ***RHBDL1---Cluster1*** | ***RHBDL1*** |  |
| cg09864712 | ***RHBDL1---Cluster1*** | ***RHBDL1*** |  |
| cg08383315 | RIC3 | *RIC3* |  |
| cg04049033 | RILP | *RILP* |  |
| cg02875297 | RLN2 | *RLN2* |  |
| cg05270634 | RND2---Cluster1 | *RND2* |  |
| cg04603184 | ***RNF182---Cluster1*** | ***RNF182*** |  |
| cg09754413 | ***RNF182---Cluster1*** | ***RNF182*** |  |
| cg06981182 | RNLS | *RNLS* |  |
| cg10148841 | ROBO4 | *ROBO4* |  |
| cg18149207 | RORC---Cluster1 | *RORC* |  |
| cg27562023 | RPH3AL---Cluster2 | *RPH3AL* |  |
| cg07441272 | ***RPL39L---Cluster1*** | ***RPL39L*** |  |
| cg07693270 | ***RPL39L---Cluster1*** | ***RPL39L*** |  |
| cg05163348 | RPP30 | *RPP30* |  |
| cg03922337 | ***RRAS2---Cluster1*** | ***RRAS2*** |  |
| cg21288099 | ***RRAS2---Cluster1*** | ***RRAS2*** |  |
| cg11492403 | RUNDC1 | *RUNDC1* |  |
| cg13102585 | RUNDC3A---Cluster1 | *RUNDC3A* |  |
| cg15271616 | RUSC2 | *RUSC2* |  |
| cg16139316 | S100A9---Cluster2 | *S100A9* |  |
| cg22266967 | S100P | *S100P* |  |
| cg18738906 | ***SCNN1A---Cluster1*** | ***SCNN1A*** |  |
| cg26215727 | ***SCNN1A---Cluster1*** | ***SCNN1A*** |  |
| cg07640473 | SEMA3F---Cluster2 | *SEMA3F* |  |
| cg09573435 | ***SEMA6A---Cluster1*** | ***SEMA6A*** |  |
| cg12928668 | ***SEMA6A---Cluster1*** | ***SEMA6A*** |  |
| cg08495878 | SERPINA4 | *SERPINA4* |  |
| cg02388150 | ***SFRP1---Cluster1*** | ***SFRP1*** |  |
| cg13398291 | ***SFRP1---Cluster1*** | ***SFRP1*** |  |
| cg15839448 | ***SFRP1---Cluster1*** | ***SFRP1*** |  |
| cg22418909 | ***SFRP1---Cluster1*** | ***SFRP1*** |  |
| cg03077062 | ***SFXN5---Cluster1*** | ***SFXN5*** |  |
| cg03856450 | ***SFXN5---Cluster1*** | ***SFXN5*** |  |
| cg07236943 | ***SGCE---Cluster2*** | ***SGCE*** |  |
| cg23096644 | ***SGCE---Cluster2*** | ***SGCE*** |  |
| cg25524350 | ***SGCE---Cluster2*** | ***SGCE*** |  |
| cg17463527 | SGK2 | *SGK2* |  |
| cg12650780 | SH3BGRL | *SH3BGRL* |  |
| cg13474750 | SH3BP1---Cluster2 | *SH3BP1* |  |
| cg13351583 | SHC3---Cluster2 | *SHC3* |  |
| cg09665351 | SHROOM2 | *SHROOM2* |  |
| cg15057581 | ***SIRPA---Cluster1*** | ***SIRPA*** |  |
| cg19237753 | ***SIRPA---Cluster1*** | ***SIRPA*** |  |
| cg05106502 | SKAP1---Cluster1 | *SKAP1* |  |
| cg02794695 | SLA | *SLA* |  |
| cg18881723 | ***SLAMF1---Cluster1*** | ***SLAMF1*** |  |
| cg20535085 | ***SLAMF1---Cluster1*** | ***SLAMF1*** |  |
| cg10694152 | SLC15A1 | *SLC15A1* |  |
| cg21992250 | SLC15A3---Cluster1 | *SLC15A3* |  |
| cg05844583 | ***SLC19A3---Cluster1*** | ***SLC19A3*** |  |
| cg25713185 | ***SLC19A3---Cluster1*** | ***SLC19A3*** |  |
| cg09326702 | SLC22A11 | *SLC22A11* |  |
| cg24838010 | SLC26A11---Cluster3 | *SLC26A11* |  |
| cg23807646 | SLC26A8 | *SLC26A8* |  |
| cg01789267 | ***SLC28A2---Cluster1*** | ***SLC28A2*** |  |
| cg19305227 | ***SLC28A2---Cluster1*** | ***SLC28A2*** |  |
| cg17550582 | SLC2A10 | *SLC2A10* |  |
| cg05674944 | ***SLC30A2---Cluster1*** | ***SLC30A2*** |  |
| cg07766612 | ***SLC30A2---Cluster1*** | ***SLC30A2*** |  |
| cg02413850 | SLC35C2---Cluster1 | *SLC35C2* |  |
| cg26233253 | SLC35E4---Cluster2 | *SLC35E4* |  |
| cg05245515 | ***SLC39A2---Cluster1*** | ***SLC39A2*** |  |
| cg05654163 | ***SLC39A2---Cluster1*** | ***SLC39A2*** |  |
| cg11800672 | SLC39A4 | *SLC39A4* |  |
| cg18960218 | SLC7A7---Cluster1 | *SLC7A7* |  |
| cg05467458 | SLC7A9 | *SLC7A9* |  |
| cg02748539 | ***SLC9A3---Cluster1*** | ***SLC9A3*** |  |
| cg23061578 | ***SLC9A3---Cluster1*** | ***SLC9A3*** |  |
| cg18108623 | SLFN11 | *SLFN11* |  |
| cg19566405 | SLFN12 | *SLFN12* |  |
| cg18972811 | SLIT2 | *SLIT2* |  |
| cg05801573 | ***SLITRK4---Cluster1*** | ***SLITRK4*** |  |
| cg12842316 | ***SLITRK4---Cluster1*** | ***SLITRK4*** |  |
| cg04478795 | ***SMO---Cluster1*** | ***SMO*** |  |
| cg15447479 | ***SMO---Cluster1*** | ***SMO*** |  |
| cg15239123 | SMOC1 | *SMOC1* |  |
| cg09214254 | ***SMOC2---Cluster1*** | ***SMOC2*** |  |
| cg11612345 | ***SMOC2---Cluster1*** | ***SMOC2*** |  |
| cg15452573 | ***SNCA---Cluster1*** | ***SNCA*** |  |
| cg26578617 | ***SNCA---Cluster1*** | ***SNCA*** |  |
| cg04684516 | ***SNCAIP---Cluster1*** | ***SNCAIP*** |  |
| cg04747322 | ***SNCAIP---Cluster1*** | ***SNCAIP*** |  |
| cg09816471 | SNN---Cluster1 | *SNN* |  |
| cg02125271 | ***SNRPN---Cluster1*** | ***SNRPN*** |  |
| cg18506672 | ***SNRPN---Cluster1*** | ***SNRPN*** |  |
| cg22555495 | ***SNRPN---Cluster1*** | ***SNRPN*** |  |
| cg08598221 | SNTB1---Cluster1 | *SNTB1* |  |
| cg27431150 | SNX20 | *SNX20* |  |
| cg19018097 | SNX32 | *SNX32* |  |
| cg27637521 | SOCS3---Cluster2 | *SOCS3* |  |
| cg06363129 | ***SOSTDC1---Cluster1*** | ***SOSTDC1*** |  |
| cg25533774 | ***SOSTDC1---Cluster1*** | ***SOSTDC1*** |  |
| cg02919422 | SOX17 | *SOX17* |  |
| cg06200339 | SOX30 | *SOX30* |  |
| cg21530890 | SOX8 | *SOX8* |  |
| cg07900766 | ***SPAG16---Cluster1*** | ***SPAG16*** |  |
| cg24664861 | ***SPAG16---Cluster1*** | ***SPAG16*** |  |
| cg18755783 | ***SPG20---Cluster1*** | ***SPG20*** |  |
| cg22609576 | ***SPG20---Cluster1*** | ***SPG20*** |  |
| cg02399455 | SRI | *SRI* |  |
| cg18727700 | ***SRPX2---Cluster1*** | ***SRPX2*** |  |
| cg19481953 | ***SRPX2---Cluster1*** | ***SRPX2*** |  |
| cg01234063 | ***ST3GAL4---Cluster1*** | ***ST3GAL4*** |  |
| cg08203715 | ***ST3GAL4---Cluster1*** | ***ST3GAL4*** |  |
| cg04434339 | ***ST6GAL2---Cluster1*** | ***ST6GAL2*** |  |
| cg25725843 | ***ST6GAL2---Cluster1*** | ***ST6GAL2*** |  |
| cg06201642 | ***ST6GALNAC5---Cluster1*** | ***ST6GALNAC5*** |  |
| cg13823136 | ***ST6GALNAC5---Cluster1*** | ***ST6GALNAC5*** |  |
| cg04380513 | ***ST8SIA4---Cluster1*** | ***ST8SIA4*** |  |
| cg13320626 | ***ST8SIA4---Cluster1*** | ***ST8SIA4*** |  |
| cg03001305 | ***STAT5A---Cluster1*** | ***STAT5A*** |  |
| cg16777510 | ***STAT5A---Cluster1*** | ***STAT5A*** |  |
| cg19728382 | STC2 | *STC2* |  |
| cg27626102 | STEAP2---Cluster2 | *STEAP2* |  |
| cg08788717 | STK33 | *STK33* |  |
| cg23670203 | STK38 | *STK38* |  |
| cg04757428 | ***STMN3---Cluster1*** | ***STMN3*** |  |
| cg08291098 | ***STMN3---Cluster1*** | ***STMN3*** |  |
| cg07190485 | ***STOM---Cluster1*** | ***STOM*** |  |
| cg18676053 | ***STOM---Cluster1*** | ***STOM*** |  |
| cg13968390 | SULT1C2---Cluster1 | *SULT1C2* |  |
| cg05472874 | ***SULT4A1---Cluster1*** | ***SULT4A1*** |  |
| cg27501458 | ***SULT4A1---Cluster1*** | ***SULT4A1*** |  |
| cg04230060 | SUSD1 | *SUSD1* |  |
| cg09160477 | ***SUSD3---Cluster1*** | ***SUSD3*** |  |
| cg26833602 | ***SUSD3---Cluster1*** | ***SUSD3*** |  |
| cg05260966 | ***SYN3---Cluster1*** | ***SYN3*** |  |
| cg05288803 | ***SYN3---Cluster1*** | ***SYN3*** |  |
| cg02104644 | SYT7---Cluster1 | *SYT7* |  |
| cg07550362 | TAC1 | *TAC1* |  |
| cg06284322 | ***TAF7---Cluster1*** | ***TAF7*** |  |
| cg19311812 | ***TAF7---Cluster1*** | ***TAF7*** |  |
| cg13282837 | TCL1A---Cluster1 | *TCL1A* |  |
| cg00187686 | ***TCN1---Cluster1*** | ***TCN1*** |  |
| cg20018806 | ***TCN1---Cluster1*** | ***TCN1*** |  |
| cg18729973 | TFF1---Cluster2 | *TFF1* |  |
| cg00386408 | ***TGFBI---Cluster1*** | ***TGFBI*** |  |
| cg21034676 | ***TGFBI---Cluster1*** | ***TGFBI*** |  |
| cg12556134 | TGIF2---Cluster2 | *TGIF2* |  |
| cg07952391 | ***THNSL2---Cluster1*** | ***THNSL2*** |  |
| cg24977027 | ***THNSL2---Cluster1*** | ***THNSL2*** |  |
| cg09805010 | ***THRB---Cluster1*** | ***THRB*** |  |
| cg13518327 | ***THRB---Cluster1*** | ***THRB*** |  |
| cg15385623 | ***THRB---Cluster1*** | ***THRB*** |  |
| cg21303011 | ***THRB---Cluster1*** | ***THRB*** |  |
| cg24120841 | ***THRB---Cluster1*** | ***THRB*** |  |
| cg15853125 | TIAM1 | *TIAM1* |  |
| cg20683151 | TM4SF20 | *TM4SF20* |  |
| cg15913671 | ***TMEM105---Cluster1*** | ***TMEM105*** |  |
| cg21591452 | ***TMEM105---Cluster1*** | ***TMEM105*** |  |
| cg04482110 | TMEM106A | *TMEM106A* |  |
| cg08261841 | TMEM139---Cluster1 | *TMEM139* |  |
| cg15518950 | TMEM171---Cluster1 | *TMEM171* |  |
| cg02244695 | ***TMEM176B---Cluster1*** | ***TMEM176B*** |  |
| cg08359956 | ***TMEM176B---Cluster1*** | ***TMEM176B*** |  |
| cg26385222 | ***TMEM176B---Cluster1*** | ***TMEM176B*** |  |
| cg02672493 | TMEM22 | *TMEM22* |  |
| cg20001829 | TMEM25---Cluster1 | *TMEM25* |  |
| cg13340269 | ***TMEM35---Cluster1*** | ***TMEM35*** |  |
| cg16510657 | ***TMEM35---Cluster1*** | ***TMEM35*** |  |
| cg18913951 | TMEM45B---Cluster2 | *TMEM45B* |  |
| cg06688396 | ***TMEM55A---Cluster1*** | ***TMEM55A*** |  |
| cg15170424 | ***TMEM55A---Cluster1*** | ***TMEM55A*** |  |
| cg16098981 | TMEM90B---Cluster1 | *TMEM90B* |  |
| cg25608949 | TMPRSS3 | *TMPRSS3* |  |
| cg26189983 | TNFRSF1B | *TNFRSF1B* |  |
| cg26738080 | TNNC1---Cluster3 | *TNNC1* |  |
| cg08696192 | TNS4 | *TNS4* |  |
| cg14494812 | TOB1---Cluster2 | *TOB1* |  |
| cg12864853 | TPBG | *TPBG* |  |
| cg00520135 | TPM1 | *TPM1* |  |
| cg05807991 | ***TRAM1L1---Cluster1*** | ***TRAM1L1*** |  |
| cg08145625 | ***TRAM1L1---Cluster1*** | ***TRAM1L1*** |  |
| cg19839588 | TRERF1---Cluster2 | *TRERF1* |  |
| cg12461141 | TRIM22 | *TRIM22* |  |
| cg13625403 | TRIM29---Cluster2 | *TRIM29* |  |
| cg00679556 | ***TRIM31---Cluster1*** | ***TRIM31*** |  |
| cg15238224 | ***TRIM31---Cluster1*** | ***TRIM31*** |  |
| cg07533148 | ***TRIM58---Cluster1*** | ***TRIM58*** |  |
| cg20855565 | ***TRIM58---Cluster1*** | ***TRIM58*** |  |
| cg03361068 | ***TRIM9---Cluster1*** | ***TRIM9*** |  |
| cg17347389 | ***TRIM9---Cluster1*** | ***TRIM9*** |  |
| cg22853986 | ***TRMT12---Cluster1*** | ***TRMT12*** |  |
| cg25375711 | ***TRMT12---Cluster1*** | ***TRMT12*** |  |
| cg07013734 | ***TRPC6---Cluster1*** | ***TRPC6*** |  |
| cg11016563 | ***TRPC6---Cluster1*** | ***TRPC6*** |  |
| cg13628514 | TRPV4 | *TRPV4* |  |
| cg13975369 | TSGA14---Cluster2 | *TSGA14* |  |
| cg00186701 | ***TSPYL5---Cluster1*** | ***TSPYL5*** |  |
| cg15747595 | ***TSPYL5---Cluster1*** | ***TSPYL5*** |  |
| cg00818693 | ***TTC14---Cluster1*** | ***TTC14*** |  |
| cg20047732 | ***TTC14---Cluster1*** | ***TTC14*** |  |
| cg12089439 | TUSC3 | *TUSC3* |  |
| cg03167763 | ***UBXN10---Cluster1*** | ***UBXN10*** |  |
| cg06911113 | ***UBXN10---Cluster1*** | ***UBXN10*** |  |
| cg08319991 | ***UCHL1---Cluster1*** | ***UCHL1*** |  |
| cg24715245 | ***UCHL1---Cluster1*** | ***UCHL1*** |  |
| cg19147390 | UHRF1 | *UHRF1* |  |
| cg22826936 | ULK2---Cluster2 | *ULK2* |  |
| cg11523020 | UPK3A | *UPK3A* |  |
| cg11108890 | VAMP5 | *VAMP5* |  |
| cg00819310 | ***VANGL1---Cluster1*** | ***VANGL1*** |  |
| cg08157638 | ***VANGL1---Cluster1*** | ***VANGL1*** |  |
| cg01485998 | VASH2---Cluster1 | *VASH2* |  |
| cg04582843 | VAV2---Cluster1 | *VAV2* |  |
| cg03591594 | ***VLDLR---Cluster1*** | ***VLDLR*** |  |
| cg05523047 | ***VLDLR---Cluster1*** | ***VLDLR*** |  |
| cg14667273 | VWA1---Cluster1 | *VWA1* |  |
| cg07744166 | ***WASF3---Cluster1*** | ***WASF3*** |  |
| cg09185773 | ***WASF3---Cluster1*** | ***WASF3*** |  |
| cg11263497 | ***WDR35---Cluster1*** | ***WDR35*** |  |
| cg24297976 | ***WDR35---Cluster1*** | ***WDR35*** |  |
| cg12955127 | WDR77 | *WDR77* |  |
| cg19427610 | WIF1---Cluster1 | *WIF1* |  |
| cg10947146 | ***XKR6---Cluster1*** | ***XKR6*** |  |
| cg11434468 | ***XKR6---Cluster1*** | ***XKR6*** |  |
| cg11049305 | ***ZC4H2---Cluster1*** | ***ZC4H2*** |  |
| cg23926715 | ***ZC4H2---Cluster1*** | ***ZC4H2*** |  |
| cg23850212 | ZFP28---Cluster2 | *ZFP28* |  |
| cg04862249 | ZFP3 | *ZFP3* |  |
| cg03454353 | ***ZFP37---Cluster1*** | ***ZFP37*** |  |
| cg17749443 | ***ZFP37---Cluster1*** | ***ZFP37*** |  |
| cg25886284 | ZFP82 | *ZFP82* |  |
| cg20880234 | ZMYM2---Cluster1 | *ZMYM2* |  |
| cg08849574 | ZNF134 | *ZNF134* |  |
| cg16638540 | ZNF135 | *ZNF135* |  |
| cg13714039 | ***ZNF14---Cluster1*** | ***ZNF14*** |  |
| cg20638426 | ***ZNF14---Cluster1*** | ***ZNF14*** |  |
| cg13108181 | ZNF141 | *ZNF141* |  |
| cg08668790 | ***ZNF154---Cluster1*** | ***ZNF154*** |  |
| cg21790626 | ***ZNF154---Cluster1*** | ***ZNF154*** |  |
| cg03712038 | ***ZNF160---Cluster1*** | ***ZNF160*** |  |
| cg12586262 | ***ZNF160---Cluster1*** | ***ZNF160*** |  |
| cg05908775 | ***ZNF211---Cluster1*** | ***ZNF211*** |  |
| cg27143938 | ***ZNF211---Cluster1*** | ***ZNF211*** |  |
| cg02497700 | ***ZNF238---Cluster1*** | ***ZNF238*** |  |
| cg23829949 | ***ZNF238---Cluster1*** | ***ZNF238*** |  |
| cg02286642 | ZNF254 | *ZNF254* |  |
| cg02959669 | ZNF256 | *ZNF256* |  |
| cg08504049 | ***ZNF264---Cluster1*** | ***ZNF264*** |  |
| cg11140785 | ***ZNF264---Cluster1*** | ***ZNF264*** |  |
| cg23257840 | ***ZNF264---Cluster1*** | ***ZNF264*** |  |
| cg16184943 | ZNF280B | *ZNF280B* |  |
| cg03565323 | ***ZNF287---Cluster1*** | ***ZNF287*** |  |
| cg23423382 | ***ZNF287---Cluster1*** | ***ZNF287*** |  |
| cg19014419 | ZNF300---Cluster2 | *ZNF300* |  |
| cg07494047 | ZNF304 | *ZNF304* |  |
| cg14218343 | ***ZNF32---Cluster1*** | ***ZNF32*** |  |
| cg26649005 | ***ZNF32---Cluster1*** | ***ZNF32*** |  |
| cg12792011 | ZNF329---Cluster1 | *ZNF329* |  |
| cg16969623 | ZNF331 | *ZNF331* |  |
| cg03100040 | ***ZNF345---Cluster1*** | ***ZNF345*** |  |
| cg27327588 | ***ZNF345---Cluster1*** | ***ZNF345*** |  |
| cg25835225 | ZNF350 | *ZNF350* |  |
| cg04488521 | ***ZNF354C---Cluster1*** | ***ZNF354C*** |  |
| cg11538128 | ***ZNF354C---Cluster1*** | ***ZNF354C*** |  |
| cg18267381 | ZNF385D---Cluster2 | *ZNF385D* |  |
| cg10332700 | ZNF415 | *ZNF415* |  |
| cg10861017 | ZNF420 | *ZNF420* |  |
| cg12104707 | ZNF426 | *ZNF426* |  |
| cg13858139 | ZNF43 | *ZNF43* |  |
| cg03355526 | ***ZNF454---Cluster1*** | ***ZNF454*** |  |
| cg23037403 | ***ZNF454---Cluster1*** | ***ZNF454*** |  |
| cg24713204 | ZNF471 | *ZNF471* |  |
| cg27480700 | ZNF480 | *ZNF480* |  |
| cg04389838 | ***ZNF501---Cluster1*** | ***ZNF501*** |  |
| cg06186861 | ***ZNF501---Cluster1*** | ***ZNF501*** |  |
| cg21672276 | ZNF502 | *ZNF502* |  |
| cg02247582 | ***ZNF512---Cluster2*** | ***ZNF512*** |  |
| cg15609321 | ***ZNF512---Cluster2*** | ***ZNF512*** |  |
| cg18611281 | ***ZNF512---Cluster2*** | ***ZNF512*** |  |
| cg22584335 | ***ZNF512---Cluster2*** | ***ZNF512*** |  |
| cg11177693 | ZNF513---Cluster1 | *ZNF513* |  |
| cg23854009 | ZNF530 | *ZNF530* |  |
| cg26309134 | ZNF542---Cluster2 | *ZNF542* |  |
| cg14386312 | ZNF544 | *ZNF544* |  |
| cg06458239 | ***ZNF549---Cluster1*** | ***ZNF549*** |  |
| cg07054095 | ***ZNF549---Cluster1*** | ***ZNF549*** |  |
| cg01644850 | ***ZNF551---Cluster1*** | ***ZNF551*** |  |
| cg18870712 | ***ZNF551---Cluster1*** | ***ZNF551*** |  |
| cg06717231 | ZNF559 | *ZNF559* |  |
| cg03884783 | ***ZNF569---Cluster1*** | ***ZNF569*** |  |
| cg18244915 | ***ZNF569---Cluster1*** | ***ZNF569*** |  |
| cg02385791 | ***ZNF570---Cluster1*** | ***ZNF570*** |  |
| cg21850254 | ***ZNF570---Cluster1*** | ***ZNF570*** |  |
| cg22472290 | ZNF577 | *ZNF577* |  |
| cg03524308 | ***ZNF585A---Cluster1*** | ***ZNF585A*** |  |
| cg10911660 | ***ZNF585A---Cluster1*** | ***ZNF585A*** |  |
| cg03147990 | ***ZNF585B---Cluster1*** | ***ZNF585B*** |  |
| cg03751813 | ***ZNF585B---Cluster1*** | ***ZNF585B*** |  |
| cg06491924 | ***ZNF613---Cluster1*** | ***ZNF613*** |  |
| cg14066280 | ***ZNF613---Cluster1*** | ***ZNF613*** |  |
| cg03293882 | ***ZNF614---Cluster1*** | ***ZNF614*** |  |
| cg25776555 | ***ZNF614---Cluster1*** | ***ZNF614*** |  |
| cg08998501 | ***ZNF615---Cluster1*** | ***ZNF615*** |  |
| cg15979098 | ***ZNF615---Cluster1*** | ***ZNF615*** |  |
| cg17456704 | ***ZNF625---Cluster1*** | ***ZNF625*** |  |
| cg17892556 | ***ZNF625---Cluster1*** | ***ZNF625*** |  |
| cg01930621 | ***ZNF649---Cluster1*** | ***ZNF649*** |  |
| cg15534084 | ***ZNF649---Cluster1*** | ***ZNF649*** |  |
| cg13636404 | ZNF655 | *ZNF655* |  |
| cg22598028 | ZNF660---Cluster2 | *ZNF660* |  |
| cg26523005 | ZNF662 | *ZNF662* |  |
| cg03289872 | ***ZNF667---Cluster1*** | ***ZNF667*** |  |
| cg05508084 | ***ZNF667---Cluster1*** | ***ZNF667*** |  |
| cg19246110 | ZNF671 | *ZNF671* |  |
| cg16708981 | ***ZNF677---Cluster1*** | ***ZNF677*** |  |
| cg18335068 | ***ZNF677---Cluster1*** | ***ZNF677*** |  |
| cg02440177 | ZNF702P | *ZNF702P* |  |
| cg15705469 | ***ZNF71---Cluster1*** | ***ZNF71*** |  |
| cg17136126 | ***ZNF71---Cluster1*** | ***ZNF71*** |  |
| cg12717203 | ***ZNF718---Cluster1*** | ***ZNF718*** |  |
| cg15792688 | ***ZNF718---Cluster1*** | ***ZNF718*** |  |
| cg04386405 | ZNF83 | *ZNF83* |  |
| cg09263755 | ZNF830 | *ZNF830* |  |
| cg15690721 | ***ZNF85---Cluster1*** | ***ZNF85*** |  |
| cg22665276 | ***ZNF85---Cluster1*** | ***ZNF85*** |  |
| cg02622316 | ***ZSCAN12---Cluster1*** | ***ZSCAN12*** |  |
| cg07660236 | ***ZSCAN12---Cluster1*** | ***ZSCAN12*** |  |
| cg06243556 | ZSCAN18 | *ZSCAN18* |  |
