# Supplementary material for: Four methylation-driven genes detected by linear discriminant analysis model from early-stage colorectal cancer and their methylation levels in cell-free DNA
Source: Front Oncol. 2022 Sep 5;12:949244. doi: 10.3389/fonc.2022.949244 (PMC9491101; doi:10.3389/fonc.2022.949244)
Supplement: Supplementary file 11 [file Table_2.docx]

| **Table S2: the Dataset Involved in This Study** | | |  |  |
| --- | --- | --- | --- | --- |
| **Dataset** | **Sample No.** | **Technology Platform** | **Comparison and usage** | **Race** |
| TCGA-COAD | Cancer: 449; Cancer-adjacent: 71 | Illumina HumanMethylation450 BeadChip; Illumina HumanMethylation27 BeadChip; | **Cancer VS cancer-adjacent**----Get LDA model based on methylation data of colon cancer:  -Randomly selected 70% of samples: train set;  -The other 30% samples: test set. | White; Black or African American; Asian; *et al* |
| GSE101764 | Cancer: 112; Cancer-adjacent: 149 | Illumina HumanMethylation450 BeadChip | **Cancer VS cancer-adjacent**----Validate the LDA model. | Europeans in Germany |
| GSE131013 | Cancer: 96; Cancer-adjacent: 96; Healthy normal: 48 | Illumina HumanMethylation450 BeadChip | **Cancer VS cancer-adjacent**----Validate LDA model; **Cancer-adjacent VS healthy normal**----Verify that the cancer-adjacent and healthy normal are homogeneous. | Europeans in Spain |
| GSE48684 | Cancer: 64; Adenomas (without CRC): 42; Cancer-adjacent:24; Healthy normal: 17 | Illumina HumanMethylation450 BeadChip | **Cancer VS cancer-adjacent**----Validate LDA model; **Cancer-adjacent VS healthy normal**----Verify that the cancer-adjacent and healthy normal are homogeneous; **Cancer VS adenoma**----LDA model cannot distinguish between cancer and adenoma; **Adenoma VS adjacent**----LDA model can distinguish adenoma and adjacent normal. | Americans around Washington |
| GSE166212 | Cancer: 32; Adenoma (with CRC): 10; Healthy normal: 6 | Infinium MethylationEPIC | **Cancer VS adenoma**----LDA model can not distinguish between cancer and adenoma; **Adenoma VS healthy normal**----LDA model can distinguish between adenoma and healthy normal. | Americans around California |
| GSE77954 | Cancer: 13; Adenoma: 12; Cancer-adjacent: 4; Adenoma-adjacent: 7; Metastasis: 9 (discarded); Metastasis-adjacent: 3 (discarded) | Illumina HumanMethylation450 BeadChip | **Cancer VS adenoma**----LDA model can not distinguish between cancer and adenoma; **Adenoma VS (cancer-adjacent + adenoma-adjacent)**---- The LDA model can distinguish adenoma from adjacent normal. | Americans around California |
| GSE139404 | High-grade adenoma (without CRC): 22; Low-grade adenoma (without CRC): 18; Healthy normal: 20 | Illumina HumanMethylation450 BeadChip | **High-grade adenoma VS low-grade adenoma**----LDA model cannot distinguish between high- and low-grade adenoma; **Low-grade adenoma VS healthy normal**----LDA model can distinguish between low-grade adenoma and healthy normal. | Chinese around Beijing |
| GSE149438 | cfDNA from: healthy people: 41 stage 0 patients: 2; stage I patients: 4; stage II patients: 10; stage III patients: 13; stage IV patients: 11 | Illumina NovaSeq 6000 | **Normal VS cancer; Normal VS stage0-2; Normal VS stage3; Normal VS stage4**----the methylation level of iRFE MDCs did not change until stage IV. | Unknown |
| GSE124600 | cfDNA from:  healthy people: 129;  stage I patients: 30;  stage II patients: 63;  stage III patients: 41;  stage IV patients: 5.  Tissue from:  Cancer-adjacent: 33  stage I: 6;  stage II: 18;  stage III: 8;  stage IV: 1. | HiSeq X Ten | **Each other;** | Chinese in southwest |
| CFEA | cfDNA from:  healthy people: 176;  CRC patients: 38 | RRBS  WGBS  MCTA-seq  MeDIP-seq  MethylCap-seq  HumanMethylation450 | **Normal VS cancer.** | Many |
